# Evaluation of the impact of the NICE head injury guidelines on inpatient mortality from traumatic brain injury: an interrupted time series analysis

**DOI:** 10.1136/bmjopen-2019-028912

**Published:** 2019-06-04

**Authors:** Carl Marincowitz, Fiona Lecky, Victoria Allgar, Trevor Sheldon

**Affiliations:** 1 Hull York Medical School, University of Hull, Hull, UK; 2 School of Health and Related Research, University of Sheffield, Sheffield, UK; 3 HYMS/Health Sciences, York University, York, UK; 4 Health Sciences, University of York, York, UK

**Keywords:** health policy, traumatic brain injury, head injury

## Abstract

**Objective:**

To evaluate the impact of National Institute for Health and Care Excellence (NICE) head injury guidelines on deaths and hospital admissions caused by traumatic brain injury (TBI).

**Setting:**

All hospitals in England between 1998 and 2017.

**Participants:**

Patients admitted to hospital or who died up to 30 days following hospital admission with International Classification of Diseases (ICD) coding indicating the reason for admission or death was TBI.

**Intervention:**

An interrupted time series analysis was conducted with intervention points when each of the three guidelines was introduced. Analysis was stratified by guideline recommendation specific age groups (0–15, 16–64 and 65+).

**Outcome measures:**

The monthly population mortality and admission rates for TBI.

**Study design:**

An interrupted time series analysis using complete Office of National Statistics cause of death data linked to hospital episode statistics for inpatient admissions in England.

**Results:**

The monthly TBI mortality and admission rates in the 65+ age group increased from 0.5 to 1.5 and 10 to 30 per 100 000 population, respectively. The increasing mortality rate was unaffected by the introduction of any of the guidelines.

The introduction of the second NICE head injury guideline was associated with a significant reduction in the monthly TBI mortality rate in the 16–64 age group (-0.005; 95% CI: −0.002 to −0.007).

In the 0–15 age group the TBI mortality rate fell from around 0.05 to 0.01 per 100 000 population and this trend was unaffected by any guideline.

**Conclusion:**

The introduction of NICE head injury guidelines was associated with a reduced admitted TBI mortality rate after specialist care was recommended for severe TBI. The improvement was solely observed in patients aged 16–64 years.

The cause of the observed increased admission and mortality rates in those 65+ and potential treatments for TBI in this age group require further investigation.

**Strengths and limitations of this study**

- This study is the first to use complete national data and the robust quasi-experimental method of interrupted time series analysis to evaluate the impact of the National Institute for Health and Care Excellence head injury guidelines.
- We adjusted our analysis for seasonality, autocorrelation and demographic changes using standard statistical techniques.
- Inpatient mortality was assessed at a population level as national data on emergency department attendance for traumatic brain injury (TBI) was unavailable and the guidelines acted to change the admission threshold for TBI identified by CT imaging.

## Background

There are approximately 2.5 million cases of traumatic brain injury (TBI) (injury to the brain/functional impairment due to external force) annually in the European Union and TBI is a leading cause of death and disability.^1^ In higher income countries the epidemiology of TBI has changed from a condition predominantly of younger males resulting from high energy trauma, to older people caused by falls.^2^

One of the important health service challenges is identifying the small proportion of patients with life-threatening TBI among the large number of patients who attend emergency departments (EDs) following head injury (blunt trauma to the head) and then ensure they receive specialist care, including neurosurgery, within a time critical period.^3^ Previous research demonstrated correctly configured emergency healthcare systems are required to deliver optimal outcomes for patients with severe TBI.^1 4^

In England, since 2003, three National Institute for Health and Care Excellence (NICE) head injury guidelines have been introduced in order to improve the ED identification and subsequent management of TBI (online supplementary material 1).^3 5–7^ These would be expected to reduce TBI deaths and unnecessary hospital admissions. All three guidelines advocated increased CT imaging of head injured patients that present with a minimally impaired conscious level equivalent to a Glasgow Coma Scale of 13–15. Increased costs from imaging were intended to be offset through reduced hospital admissions.^8^ The 2007 guideline additionally recommended that patients with severe TBI should be managed in specialist neuroscience centres. At the time of implementation, concerns were raised that guideline recommendations were based on studies in subgroups and lacked supporting level 1 evidence.^4 9 10^ Evaluation of the impact of these guidelines on national rates of TBI admissions and patient outcomes is therefore needed.

10.1136/bmjopen-2019-028912.supp1 Supplementary data

We describe the first study to use complete national data and interrupted time series analysis to evaluate the impact of early TBI management guidelines on patient outcomes and admission rates for all severities of TBI.

## Methods

### Data set

Hospital episode statistics (HES) are collected on all inpatients in England. The Office for National Statistics (ONS) has computerised International Classification of Diseases (ICD) coding of cause of death information recorded on death certificates.

We used individual patient level HES data provided by NHS Digital on all emergency inpatient hospital admissions in England from April 1998 to April 2017. Reason for admission is recorded using ICD10 coding. For patients with ICD10 diagnostic codes: S00–S09 (indicating TBI) or T04.0 and T06.0 (crushing injury to the head) who died up to 30 days from discharge ONS cause of death was also provided.^11^ ONS coding changed from ICD9 to ICD10 in 2001.

### Deaths attributable to TBI

Online supplementary material 2 summarises how deaths attributable to TBI over the study period were identified. A total of 852 646 deaths linked to admissions for head injury were identified by NHS Digital. We searched all cause of death fields for ICD9 and ICD10 codes defined by the Centers for Disease Control and Prevention (CDC) as indicating a death attributable to TBI (table 1).^12^ When any of these ICD codes were present the death was coded as attributable to TBI. A total of 34 659 deaths attributable to TBI were identified, and these were linked to their last recorded admission date as a proxy for when the injury and death occurred. This was not possible for 2862 patients. Neonatal deaths were excluded from analysis due to differences in cause of death coding.

**Table 1:** Annual numbers of deaths and admissions from traumatic brain injury in England (estimated from data set provided by NHS Digital)

Year	Admissions all age groups	Admissions 0–15	Admissions 16–64	Admissions 65+	Death all age groups	Deaths 0–15	Deaths 16–64	Deaths 65+
*1998	47 820	17 739	22 348	7631	677	45	307	331
1999	63 599	23 848	29 088	10 553	964	71	446	453
2000	60 001	21 774	27 793	10 280	1076	69	492	525
2001	58 497	21 065	26 553	10 774	1105	62	519	532
2002	55 941	19 579	25 808	10 424	1178	46	508	634
2003	60 336	19 630	28 405	12 239	1294	51	521	729
2004	68 662	20 361	33 298	14 937	1342	49	568	734
2005	75 391	20 417	36 832	18 093	1484	43	606	840
2006	77 333	19 696	38 005	19 566	1570	49	610	917
2007	75 219	18 128	36 473	20 566	1665	39	624	1012
2008	74 158	17 481	34 657	21 938	1621	26	564	1036
2009	81 218	18 111	37 178	25 848	1739	35	603	1105
2010	81 032	18 008	35 064	27 856	1817	29	530	1260
2011	82 093	18 604	33 989	29 390	1879	35	500	1354
2012	76 925	16 453	30 475	29 901	2025	27	525	1474
2013	76 429	15 966	28 983	31 379	2204	27	497	1687
2014	79 372	15 535	28 833	34 890	2361	15	462	1886
2015	76 648	13 630	27 517	35 357	2610	18	493	2102
2016	74 242	13 120	25 228	35 488	2682	30	511	2145
*2017	16 247	2619	5483	8037	504		79	420

*Data are from April 1998 to March 2017, so 1998 and 2017 are part years and small number have been suppressed in accordance with NHS digital guidance.

ICD9 definition TBI: 800, 801, 803, 804, 850, 851, 852, 853, 854, 905.0, 907.0 and 873 ICD10 definition TBI: S01.0−S01.9, S02.0, S02.1, S02.3, S02.7-S02.9, S04.0, S06.0−S06.9, S07.0, S07.1, S07.8, S07.9, S09.7−S09.9, T01.0, T02.0, T04.0, T06.0, T90.1, T90.2, T90.4, T90.5, T90.8 and T90.

### Admissions attributable to TBI

The same ICD10 codes were used to identify patients admitted with TBI (table 1).^12^ We searched the primary diagnostic field in the inpatient HES data set for these codes and when present the reason for admission was coded as due to TBI. Data were cleaned and continuous inpatient spells (CIPS) were created for patients admitted with TBI using the approach outlined by Castelli, Laudicella and Street as this includes transfers within CIPS.^13^ 1361537 CIPS for TBI were identified for 1245720 patients. Following cleaning, 402 CIPs were found to have admission dates prior to April 1998 and were excluded. Demographic and comorbidity information was calculated from the first consultant episode of a CIP. This included the monthly proportion of TBI admissions for males, monthly median age of admissions and mean monthly admission Charlson comorbidity index score (using ICD10 code definitions and weights used to calculate the summary hospital-level mortality indicator).^14^ This was compared with adjustment using a modified Charlson comorbidity index derived from the national (Trauma Audit and Research Network; TARN) trauma registry.^15^

### Outcomes

The monthly number of patients with deaths and admissions attributable to TBI between April 1998 and March 2017 was calculated. These were stratified into guideline specific age groups: 0–15, 16–64 and 65+. Monthly mortality and admission rates were calculated per 100 000 population using Nomis ONS mid-year population estimates for England for each age group.^16^

### Statistical analysis

A monthly time series of the mortality rate for TBI was plotted for the study period. Interrupted times series (ITS) analysis was conducted assessing the impact of the NICE guidelines using the ITSA package in STATA V.14.^17^ ITS analysis is a robust and increasingly used quasi-experimental method for the evaluation of health policies and allows causality to be attributed to an intervention introduced at a specific time point.^18^

The ITS model included three intervention time points corresponding to the introduction of each guidelines in: June 2003, September 2007 and January 2014. Analysis was conducted separately for the 0–15, 16–64 and 65+ age groups. A segmented regression model predicting the mortality rate and hospital admission rate for TBI per 100 000 population in each age group per month was estimated.^18^ A discontinuity in the gradient (trend) or intercept (level) of the fitted model was tested for at the time point when each guideline was introduced, and discontinuities in the model were measured in the monthly rate of the outcome per 100 000 population.

To adjust for potential changes in the composition of the TBI population that could possibly affect the risk of mortality a further ITS model predicting the TBI mortality rate adjusted for % male, median age and mean Charlson comorbidity index score of patients admitted with TBI was fitted. Stratification by age group and intervention points were identical to the previous analysis.

In all analyses, autocorrelation of the residuals was assessed using the Durbin-Watson and Rho statistic. Throughout we used the Prais-Winsten transformation adjustment for auto-correlation due to improved fit of the model, deviation from a Durbin-Watson statistic of 2 and a non-statistically significant Rho statistic.^18^ Seasonality was assessed by introducing a dummy variable to the model in which winter months (December, January and February) were coded 1 and was included in the model when statistically significant.^19^ To assess for possible implementation lags a sensitivity analysis was performed for all models in which the 12 months immediately following the introduction of a guideline were removed.^18^

### Patient and public involvement

The Hull and East Yorkshire NHS Trust Trans-Humber Consumer Research Panel and Hull branch of the Headway charity were consulted in the initial stages of developing the research questions addressed in this study. These patient groups highlighted that although national head injury guidelines seemed evidence based, there appeared to be little evidence to show they had achieved their aims.

## Results

### Mortality rate

**Figure 1:**
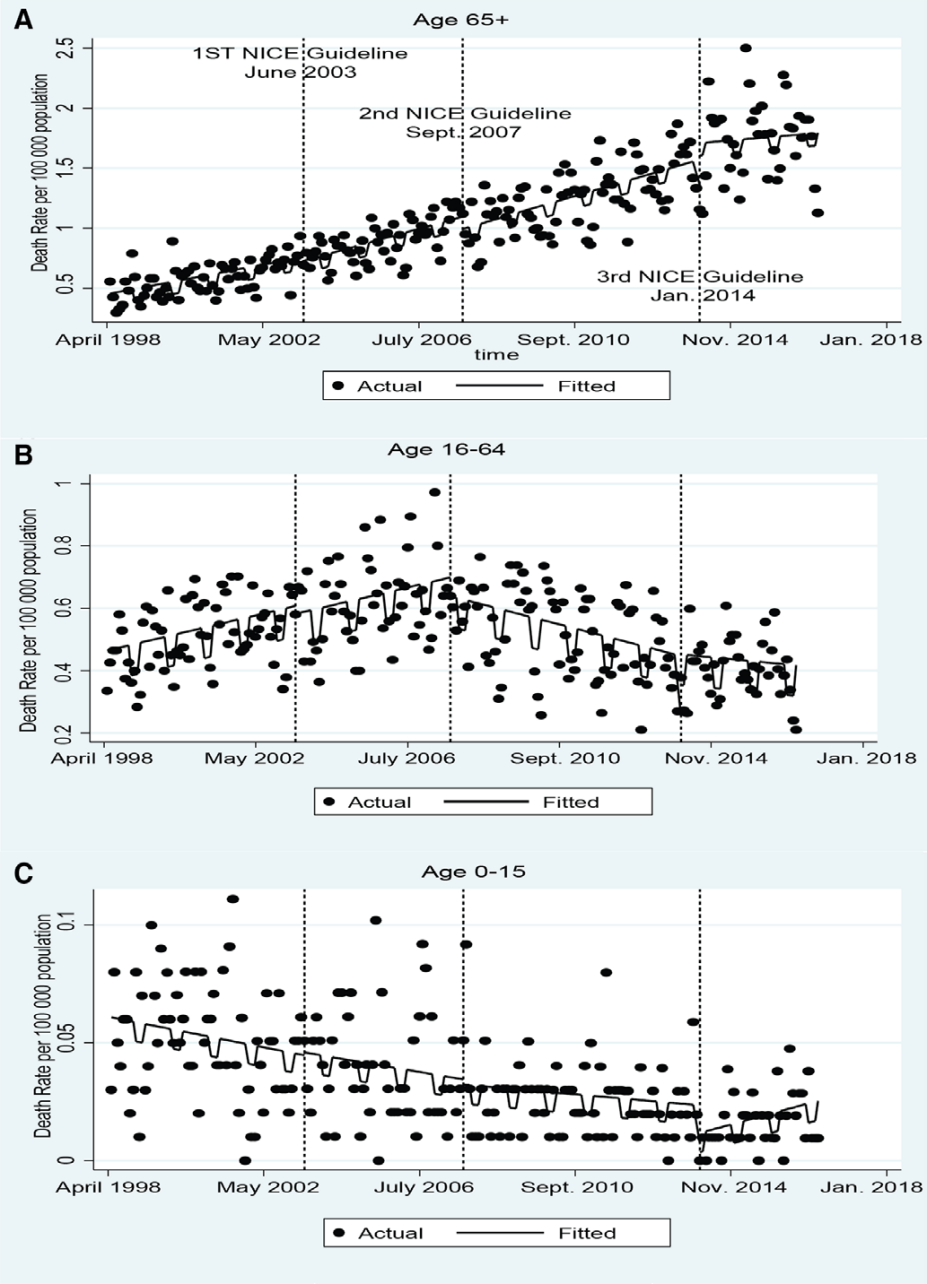
The impact of the National Institute for Health and Care Excellence head injury guidelines on monthly traumatic brain injury mortality rate per 100 000 population.

Table 1 shows the annual number and online supplementary material 3 shows the annual rates of deaths and hospital admissions for TBI. The proportion of all TBI annual admissions for patients 65+ increased from 17% in 1998 to 48% in 2016 and the proportion of all TBI deaths in this age group increased from 49% to 78% over the same period. Figure 1 shows the monthly mortality rate per 100 000 population in each age group. Table 2 shows the results of the unadjusted interrupted time series assessing the impact of the NICE head injury guidelines. Deaths were more likely to occur in non-winter months in all age groups and so the figures are seasonally adjusted.

**Table 2:** The impact of the National Institute for Health and Care Excellence head injury guidelines on monthly traumatic brain injury mortality rate per 100 000 population

Age band	Winter effect	Initial trend	First NICE guideline	Second NICE guideline	Third NICE guideline	Durbin-Watson statistic
65+	−0.1 (95% CI:−0.16 to −0.04) p<0.01	0.005 (95% CI:0.002 to 0.008) p<0.01	Change level: −0.034 (95% CI:−0.21 to 0.14) p=0.71 Change trend: 0.002 (95% CI:−0.003 to 0.008) p=0.43	Change level: −0.1 (95% CI: −0.27 to 0.07) p=0.24 Change trend: 0.0004 (95% CI: −0.005 to 0.006) p=0.89	Change level: 0.13 (95% CI:−0.04 to 0.32) p=0.14 Change trend: −0.005 (95% CI:−0.01 to 0.002) p=0.14	Untransformed 1.57 Prais-Winsten 1.86
16–64	−0.1 (95% CI: −0.13 to −0.06) p<0.01	0.002 (95% CI:0.001 to 0.004) p<0.01	Change level: −0.03 (95% CI: −0.11 to 0.06) p=0.57 Change trend: −0.00002 (95% CI: −0.003 to 0.003) p=0.99	Change level: −0.06 (95% CI:−0.15 to 0.003) p=0.17 Change trend: −0.005 (95% CI:−0.007 to −0.002) p<0.01	Change level: 0.005 (95% CI:−0.087 to 0.096) p=0.92 Change trend: 0.002 (95% CI:−0.002 to 0.005) p=0.38	Untransformed 1.79 Prais-Winsten 1.95
0–15	−0.01 (95% CI:−0.01 to −0.003) p<0.01	−0.0003 (95% CI: −0.0005 to −0.00001) p=0.04	Change level: 0.001 (95% CI: −0.01 to 0.01) p=0.18 Change trend: 0.00004 (95% CI:−0.0004 to 0.0004) p=0.17	Change level: −0.0021 (95% CI: −0.01 to 0.01) p=0.74 Change trend 0.0001 (95% CI:−0.0003 to 0.0005) p=0.58	Change level: −0.01 (95% CI:−0.03 to 0.002) p=0.09 Change trend: 0.0005 (95% CI: −0.00005 to 0.001) p=0.08	Untransformed 2.12 Prais-Winsten 1.99

The trends in mortality rate and impact of the guidelines varied between age groups. In the 65+ age group the monthly TBI mortality rate increased from around 0.5 to over 1.5 per 100 000 population over the time period (figure 1A). This was accompanied by an increase in the Charlson score of patients 65+ admitted with TBI (online supplementary material 4). The NICE head injury guidelines were not associated with statistically significant changes in the level or trend in the mortality rate (table 2). Subgroup analysis of patients aged 65–84 and 85+ showed that the increase in the mortality rate was greater in those 85+, from around 1 to over 6 per 100 000 population but similar changes were associated with the introduction of the guidelines to the whole 65+ population (online supplementary material 5).

The second guideline was found to be associated with a large reduction in mortality in the 16–64 age group (figure 1B). Before the guideline, the monthly mortality rate was increasing but the introduction of the second NICE guideline is associated with a reversal of this trend (−0.005; 95% CI:−0.002 to −0.007) (table 2). The reduction in mortality appears to slow at the time of the introduction of the third NICE guideline but this was not statistically significant. There was an increase in age of patients in the 16–64 age group admitted with TBI but no change in the Charlson comorbidity score over the period (online supplementary material 4).

In the 0–15 age group the mortality rate fell continuously over the time period from around 0.05 to 0.01 per 100 000 population (figure 1C). There were fewer monthly numbers of deaths and so more random variability in rates. None of the guidelines were associated with a statistically significant change in the level or trend in the mortality rate (table 2), though the high random variability meant we had lower statistical power to detect such changes as statistically significant.

Adjustment for the monthly median age, mean Charlson Score and proportion of male admissions for TBI did not materially alter the estimates associated with the introduction of guidelines in any of the age groups (online supplementary material 6). In the 16–64 age group the estimate of the reversal in trend in mortality rate associated with the second guideline, −0.006 (95% CI:−0.008 to −0.003), was similar to the unadjusted analysis. The levelling off in the rate of reduction in mortality in the 16–64 age group associated with the third NICE guideline became marginally statistically significant, although the estimate is similar, 0.003 (95% CI: 0.00005 to 0.007). No adjustment was made for the standard Charlson score in the paediatric and 16–64 age groups as it did not change over time. The monthly mean trauma modified Charlson score in the 16–64 age group increased slightly from 0 to 1 and adjustment for this increased the estimated size of reversal in mortality trend associated with the second NICE guideline, −0.008 (95% CI: −0.01 to −0.005), (online supplementary material 4). The sensitivity analysis for the effect of implementation lags did not affect the estimates associated with the introduction of any guideline (online supplementary material 7).

### Admission rate

**Figure 2:**
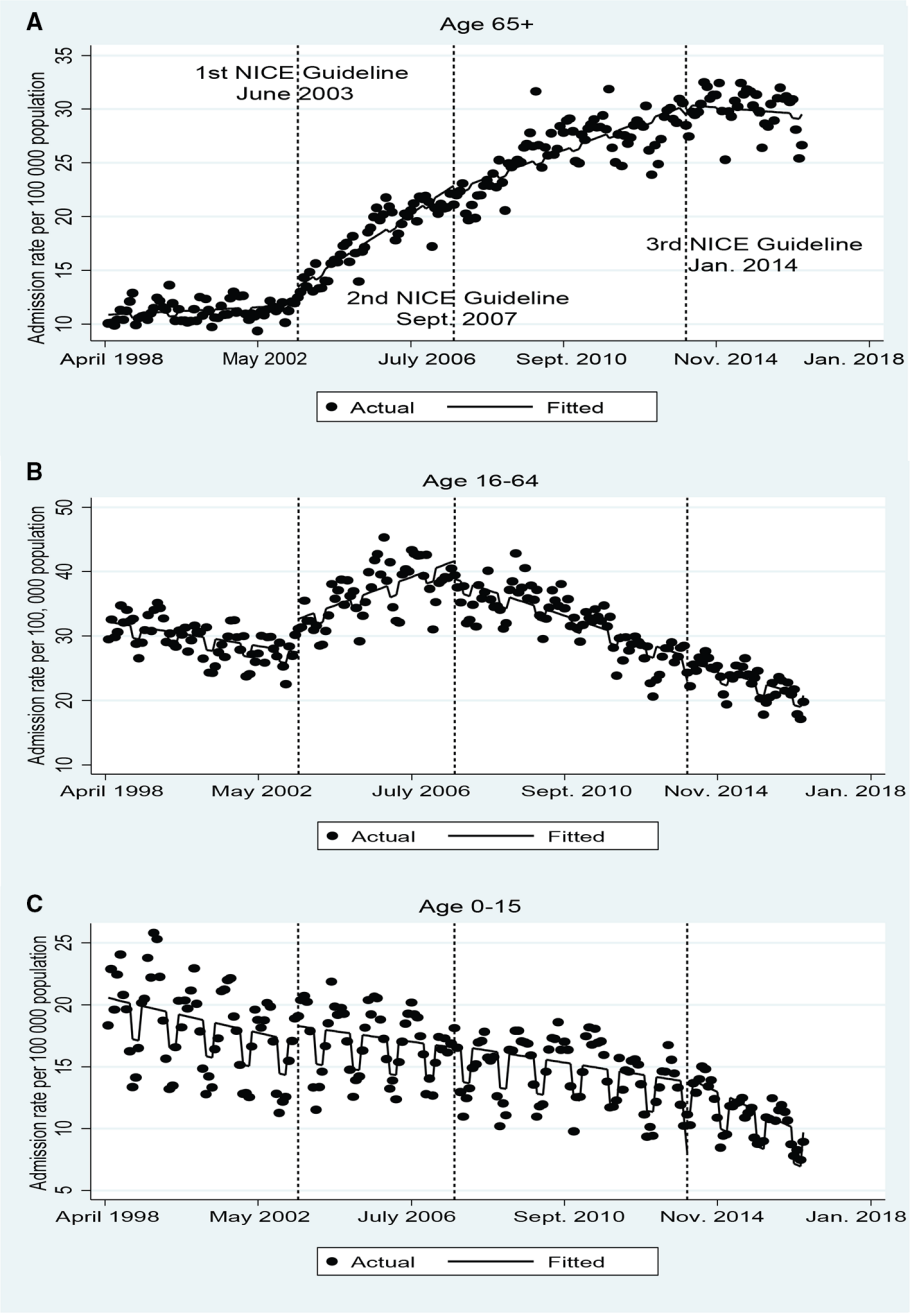
The impact of the National Institute for Health and Care Excellence head injury guidelines on monthly traumatic brain injury hospital admissions per 100 000 population.

Figure 2 shows the trends in monthly TBI admissions stratified by age group and table 3 presents estimates of the change in admission rate associated with the introduction of each head injury guideline iteration. The admission rate increased threefold (from around 10 per 100 000 to 30 per 100 000) in the 65+ age group. The introduction of the first NICE guideline is associated with large increasing trends in monthly TBI admissions per 100 000 population in both the 65+ age group (0.17: 95% CI: 0.11 to 0.22) and the 16–64 age group (0.25: 95% CI: 0.16 to 0.34) (table 3).^20^ The subsequent two guidelines are associated with significant reductions in this trend and admission rates level off following the third guideline in the 65+ age group (table 3 and figure 2A). In the 16–64 age group, the TBI admissions trend reverses and declines after the second NICE guideline (−0.33: 95% CI: −0.42 to −0.25) (table 3 and figure 2B).

**Table 3:** The impact of the National Institute for Health and Care Excellence head injury guidelines on monthly traumatic brain injury hospital admission rate per 100 000 population

Age band	Winter effect	Initial trend	First NICE guideline	Second NICE guideline	Third NICE guideline	Durbin-Watson statistic
65+	−0.44 (95% CI: −0.94 to 0.06) p=0.08	0.01 (95% CI: −0.02 to 0.05) p=0.42	Change level: 1.71 (95% CI:−0.01 to 3.44) p=0.05 Change trend: 0.17 (95% CI: 0.11 to 0.23) p<0.01	Change level: −0.4 (95% CI: −2.08 to 1.27) p=0.64 Change trend: −0.08 (95% CI: −0.13 to −0.03) p<0.01	Change level: 0.04 (95% CI:−1.73 to 1.82) p=0.96 Change trend: −0.13 (95% CI:−0.2 to −0.05) p<0.01	Untransformed 1.1 Prais-Winsten 2.09
16–64	−1.92 (95% CI: −2.77 to −1.07) p<0.01	−0.08 (95% CI: −0.13 to −0.02) p<0.01	Change level: 5.21 (95% CI: 2.53 to 7.89) p<0.01 Change trend: 0.25 (95% CI: 0.16 to 0.34) p<0.01	Change level: −2.76 (95% CI:−5.35 to −0.16) p=0.04 Change trend: −0.33 (95% CI: −0.42 to −0.25) p<0.01	Change level: −0.72 (95% CI: −3.49 to 2.03) p=0.61 Change trend: 0.02 (95% CI:−0.09 to 0.13) p=0.73	Untransformed 1.35 Prais-Winsten 2.11
0–15	−2.87 (95% CI: −3.40 to −2.34) p<0.01	−0.06 (95% CI:−0.11 to −0.01) p=0.03	Change level: 1.3 (95% CI: −1.03 to 3.63) p=0.27 Change trend: 0.02 (95% CI: −0.07 to 0.11) p=0.61	Change level: 0.19 (95% CI: −2.09 to 2.47) p=0.87 Change trend −0.005 (95% CI: −0.08 to 0.08) p=0.91	Change level: 0.34 (95% CI:−2.03 to 2.72) p=0.78 Change trend: −0.08 (95% CI: −0.19 to 0.03) p=0.17	Untransformed 1.07 Prais-Winsten 1.70

In the 0–15 age group TBI admissions steadily fall over the study period from around 20 per 100 000 to 10 per 100 000 (figure 2C), and is unaffected by the introduction of the guidelines (table 3).

A sensitivity analysis for implementation lags in which the 12 months following the introduction of a guideline were removed from the analysis did not materially change the estimates associated with the introduction of the guidelines in any age group (online supplementary material 8).

## Discussion

### Summary

To our knowledge this is the first study to use national population based data and interrupted time series analysis to evaluate the impact of the NICE head injury guidelines in England. The second NICE guideline was associated with a reduction in the admitted TBI mortality rate in the 16–64 age group at a population level (table 2). We found no other impact on mortality associated with the three guideline iterations.

There was a continual and significant increase in TBI mortality and admission rates in the 65+ age group and a contrasting falling trend in mortality and admission rates in children (figure 1 and figure 2). Both trends began before the introduction of the NICE guidelines and were not significantly affected by any of the three iterations. In both the 16–64 and 65+ age groups there was a large increase in hospital admissions for TBI at the time the first NICE guideline was introduced (figure 2).

Increased imaging was intended to reduce hospital admissions by reducing diagnostic uncertainty but the first NICE guideline coincided with the introduction of the 4 hour target.^8 20^ We have shown, using Scottish data assessing the impact of similar Scottish Intercollegiate Guidelines Network (SIGN) guidelines (introduced at a different time to the 4-hour target), that the 4-hour target acted to undermine this reduction and cause a large increase in hospital admissions.^21^ No mortality benefit was found at the time of the introduction of the 4-hour target in England.

Later guidelines were associated with a reduction in hospital admissions rates in both adult populations assessed (figure 2). Further increases in CT imaging may have reduced hospital admissions, as intended, by reducing diagnostic uncertainty in the ED, without the distorting effect of the 4-hour target introduction.

### Strengths

We used complete national data for England to assess the impact of the NICE head injury guidelines on mortality after admission for TBI at a population level. We have used individual level patient data to define TBI deaths and admissions. We controlled for seasonal factors and auto-correlation using established techniques.^18^ We used mid-year population estimates to adjust for changes in the demography of England’s population.

### Weaknesses

Ideally, we would have estimated the impact of the guidelines on case fatality, as this better measures the impact on the population at risk. The impact on case fatality of those attending ED with TBI could not be estimated because ED data were not collected until 2007. The impact on case fatality of those admitted with TBI could be estimated but because the guidelines resulted in changes in admissions policies and rates, the rate of deaths per admission is difficult to interpret. Instead we analysed the impact on the population TBI mortality rate, as this represents the best available unbiased measure of the guidelines’ impact. This outcome may be affected by changes in the underlying population TBI rate that we are unable to account for, although annual attendances to the ED for head injury gradually smoothly increased over the study period (online supplementary material 9). We were unable to assess possible impact on disability or other patient reported outcomes, as they are not routinely collected.

ONS linked HES data is based on routinely collected administrative data; these can suffer from poor accuracy of injury coding.^22^ This is particularly likely in older patients with multimorbidity (TARN, personal communication 2018). Random poor coding, as opposed to a discrete and systematic change in coding practice, however, is unlikely to account for discontinuities observed at the specific time points of interest but may make a discontinuity harder to detect. ONS changed from ICD9 to ICD10 coding of cause of death in 2001. A sensitivity analysis excluding the period that used ICD9 coding did not materially alter the estimate of the reversal in mortality trend associated with the second guideline in the 16–64 age group. We are unaware of other significant changes to coding practice in the HES or ONS data during the study period. The limitations of HES data mean that mortality rates could not be adjusted for anatomical severity of brain injury and presenting physiology. However, adjustment for other known predictors of TBI mortality did not materially change estimates associated with the introduction of the guidelines and we are unaware of evidence that the prevalence of these factors changed at the point individual guidelines were introduced.

The impact of guidelines is limited by how well they are implemented. The NICE head injury guidelines have been found to be well implemented,^23^ although with less compliance to CT imaging recommendations in the paediatric population.^23 24^ There is evidence that each guideline caused step increases in CT head scanning in other age groups, particularly in those 65+.^10 25^

The reconfiguration of the trauma network in England in 2012 is a co-intervention which could affect the TBI mortality rate.^26^ However, we found no impact on mortality associated with the 2014 NICE guideline introduced around this time. Apart from the introduction of the 4-hour ED admissions target in 2004, we are unaware of any other co-interventions that occurred around the time the NICE guidelines were introduced which could account for the observed discontinuities in mortality and hospital admissions.

### Comparison to previous literature

Few previous studies assess the impact of the NICE head injury guidelines (see table 4).^9^ A cohort study using TARN national registry data suggested the increased rate of transfer of severe TBI patients to neuroscience centres between 2003 and 2009 was associated with a halving of severe TBI case fatality.^4^ TARN data were only collected at approximately half of hospitals in England until 2012 and on a TBI patient subset. Our study, using complete national data and interrupted time series analysis, found that guideline recommended management of patients with severe injuries in specialist centres only reduced the mortality rate in the 16–64 age group.

**Table 4:** Comparison to previous literature

Previous study			Current study
	Study population	Findings	Findings
Fuller *et al,* 2009^4^	TARN eligible patients at TARN submitting hospitals (approx. 50% England) between 2003 and 2009.	From the period 2004 onwards as the proportion of patients with TBI transferred and managed in neuroscience centres increased and the risk adjusted mortality rate for TBI fell.	Complete national data for all hospital in England. A reversal in trend in the mortality rate in the 16–64 age group when the second NICE guideline recommending management of patients with severe injuries in specialist centres was introduced.
Marlow *et al* 24	Patients aged <16 with ICD10 codes indicating head injury admitted to hospitals in England between 2000 and 2011.	Assessed the annual rate of inpatient deaths (all-cause mortality) for patients admitted with ICD10 codes indicating head injury. Found the death rate fell across the time period, but there was only a statistically significant reduction in the death rate after the 2007 NICE head injury guideline.	The inpatient TBI mortality rate (as indicated by coding of death certificates) for patients aged <16 fell from 1998 to 2017 and was unaffected by the introduction of the NICE guidelines.
The Trauma Audit and Research network report: major trauma in older people^25^	TARN eligible patients at TARN submitting hospitals between 2005 and 2014 (all hospitals in England by 2014)	A large increase in major trauma, including TBI, in patients 65+, disproportionate to UK population demographic changes. Hypothesised due to increased case ascertainment due to more liberal CT imaging.	We found a large increase in the admission rate for TBI in those 65+ from 10 per 100 000 population to 30 per 100 000 population between 2002 and the point the third NICE guideline was introduced in 2014.

A paediatric study analysing English HES data from 2000 to 2011 found a reduction in annual mortality during admissions for head injury after the introduction of 2007 NICE guideline.^24^ We found a fall in the mortality rate over the study period in the 0–16 age group which was unaffected by any guideline. This may reflect the greater number of data points we used to estimate the time-dependent model and use of interrupted time series analysis to assess for discontinuities. We also used ONS linked HES data to identify deaths directly attributable to TBI up to 30 days following discharge. The observed decreasing mortality and admission rates may reflect improving clinical management or a reduction in TBI in this age group due to improving road traffic safety during the study period.^24^

An economic evaluation of the NICE guidelines found them to be cost effective due to a reduction in hospital admissions predicted from early single centre studies and improved outcomes.^8 10^ A subsequent study using HES data found hospital admissions for head injury increased after the introduction of the first NICE guideline.^11^ The similar increase in adult TBI admissions we found associated with the first NICE guideline probably is due the 4-hour target.^21^ We found subsequent NICE guidelines improved outcomes and reduced hospital admissions in the 16–64 but not the 65+ age group, implying the guidelines were less cost effective in older patients.

Other studies using TARN data have found increases in TBI in patients 65+ disproportionate to population changes and it has been suggested that better case ascertainment due to increased CT imaging in older patients may account for this.^2 25^ The large increase in admissions for TBI for those 65+ we found at the point the first guideline was introduced, although boosted by the 4-hour target, supports this (figure 2A and table 3). The lack of improvement in admitted TBI mortality in older patients following the second NICE guideline could either result from unequal access to treatment in specialist centres or such treatment appearing to be less effective in this group. The TARN older persons audit found patients aged over 60 to be less likely to be managed in major trauma centres (where neurosurgical units are located in England) and more likely to experience delays in investigation and be treated by junior staff.^25^ However, other studies have found age to be an independent predictor of mortality that is unaffected by early treatment in neuroscience centres.^27 28^

We are unaware of comparable national evaluations of the impact of head injury guidelines. Evaluations of International Brain Trauma Foundation guidelines, particularly in the USA, have utilised evidence from single centre studies or subsets of patients.^23 29 30^ Evaluation of their national impact has not been possible due to their variable implementation.^23 30^

### Implications

We found evidence that only the second NICE head injury guideline was associated with a change in population-based TBI mortality. This guideline contained a recommendation for increased management of severe TBI in specialist centres. Much research has focused on determining which head injured patients require CT imaging.^3 31^ Increased diagnosis by itself, however, without a change in subsequent patient management was not associated with improved outcomes in our analysis. Even if apparent increases in TBI rates in older patients reflect the identification of previously unmet need, this still represents a significant health service challenge. Routine ICD coding of TBI is particularly problematic in this group and robust evaluation of treatment in specialist neuroscience centres and other interventions may be required to improve outcomes in older TBI patients. The UK, however, has one of the lowest numbers of ICU beds per population in Europe and when the 2007 guideline recommendation was made concerns were raised about the system meeting demand.^9 32^ Research needs to focus on how to best configure and ration specialist services for TBI in a transparent and evidence-based way.

## Conclusion

This first national evaluation suggests that the introduction of the second NICE head injury guideline was associated with a reduction in the admitted TBI mortality rate in the 16–64 age group and a reduction in TBI admissions in England. The guidelines were not associated with significant changes in the secular trend for TBI admissions and subsequent mortality in children and those aged 65+. Research is needed to identify clinically and cost-effective management approaches for TBI in older patients.

## Supplementary Material

Reviewer comments

Author's manuscript

## References

[1] Maas AIR, Menon DK, Adelson PD, et al Traumatic brain injury: integrated approaches to improve prevention, clinical care, and research. Lancet Neurol 2017;16:987–1048. 10.1016/S1474-4422(17)30371-X 29122524

[2] Lawrence T, Helmy A, Bouamra O, et al Traumatic brain injury in England and Wales: prospective audit of epidemiology, complications and standardised mortality. BMJ Open 2016;6:e012197 10.1136/bmjopen-2016-012197 PMC5168492 27884843

[3] NICE. National Clinical Guidance Centre. (2014). CG 176 Head Injury Triage, assessment, investigation and early management of head injury in children, young people and adults. UK: National Institute for Health and Care Excellence, 2014. 25340248

[4] Fuller G, Bouamra O, Woodford M, et al Temporal trends in head injury outcomes from 2003 to 2009 in England and Wales. Br J Neurosurg 2011;25:414–21. 10.3109/02688697.2011.570882 21513451

[5] NICE. National Clinical Guidance Centre. (2007). Guideline CG56: Head injury: Triage, assessment, investigation and early management of head injury in infants, children and adults. UK: National Institute for Health and Care Excellence, 2007.

[6] NICE. National Clinical Guidance Centre. (2003). Clinical guideline 4: Head injury: triage, assessment, investigation and early management of head injury in infants, children and adults. UK: National Institute for Health and Care Excellence, 2003.

[7] Hodgkinson S, Pollit V, Sharpin C, et al Early management of head injury: summary of updated NICE guidance. BMJ 2014;348:g104 10.1136/bmj.g104 24452622

[8] Pandor A, Goodacre S, Harnan S, et al Diagnostic management strategies for adults and children with minor head injury: a systematic review and an economic evaluation. Health Technol Assess 2011;15:1–202. 10.3310/hta15270 PMC4781048 21806873

[9] Barratt H, Wilson M, Moore F, et al The implications of the NICE guidelines on neurosurgical management for all severe head injuries: systematic review. Emerg Med J 2010;27:173–8. 10.1136/emj.2009.075382 20304874

[10] Hassan Z, Smith M, Littlewood S, et al Head injuries: a study evaluating the impact of the NICE head injury guidelines. Emerg Med J 2005;22:845–9. 10.1136/emj.2004.021717 16299190 PMC1726640

[11] Goodacre S Hospital admissions with head injury following publication of NICE guidance. Emerg Med J 2008;25:556–7. 10.1136/emj.2007.055723 18723700

[12] Coronado VG, Xu L, Basavaraju SV, et al Surveillance for traumatic brain injury-related deaths--United States, 1997-2007. MMWR Surveill Summ 2011;60:1–32. 21544045

[13] Castelli A, Laudicella M, Street A Measuring NHS Output growth. CHE research paper 2008: University of York, 2008:43.

[14] Campbell MJ, Jacques RM, Fotheringham J, et al Developing a summary hospital mortality index: retrospective analysis in English hospitals over five years. BMJ 2012;344:e1001 10.1136/bmj.e1001 22381521 PMC3291118

[15] Bouamra O, Jacques R, Edwards A, et al Prediction modelling for trauma using comorbidity and ’true' 30-day outcome. Emerg Med J 2015;32:933–8. 10.1136/emermed-2015-205176 26493123

[16] Office of National Statistics. https://www.nomisweb.co.uk/articles/924.aspx.

[17] Linden A Conducting interrupted time-series analysis for single- and multiple-group comparisons. Stata J 2015;15:480–500. 10.1177/1536867X1501500208

[18] Bernal JL, Cummins S, Gasparrini A Interrupted time series regression for the evaluation of public health interventions: a tutorial. Int J Epidemiol 2017;46:dyw098 10.1093/ije/dyw098 PMC5407170 27283160

[19] Hamilton I, Lloyd C, Hewitt C, et al Effect of reclassification of cannabis on hospital admissions for cannabis psychosis: a time series analysis. Int J Drug Policy 2014;25:151–6. 10.1016/j.drugpo.2013.05.016 23867051

[20] Comptroller General. Improving Emergency Care in England. National Audit Office 2004; HC 1075 Session 2003-2004. 2004 https://www.nao.org.uk/wp-content/uploads/2004/10/03041075.pdf.

[21] Marincowitz C, Lecky FE, Morris E, et al Impact of the SIGN head injury guidelines and NHS 4-hour emergency target on hospital admissions for head injury in Scotland: an interrupted times series. BMJ Open 2018;8:e022279 10.1136/bmjopen-2018-022279 PMC6318526 30580260

[22] Hand DJ Statistical challenges of administrative and transaction data. J R Statist Soc A 2018:1–24.

[23] Cnossen MC, Scholten AC, Lingsma HF, et al Adherence to Guidelines in Adult Patients with Traumatic Brain Injury: a living systematic review. J Neurotrauma 2016 (Published 4 Dec 2015). 10.1089/neu.2015.4121 PMC8054518 26431625

[24] Marlow R, Mytton J, Maconochie IK, et al Trends in admission and death rates due to paediatric head injury in England, 2000-2011. Arch Dis Child 2015;100:1136–40. 10.1136/archdischild-2015-308615 26272910

[25] The Trauma and Audit Research Network Report 2017. Major Trauma in Older People. 2017 https://www.tarn.ac.uk/content/downloads/3793/Major%20Trauma%20in%20Older%20People%202017.pdf.

[26] Yiannoullou P, Hall C, Newton K, et al A review of the management of blunt splenic trauma in England and Wales: have regional trauma networks influenced management strategies and outcomes? Ann R Coll Surg Engl 2017;99:63–9. 10.1308/rcsann.2016.0325 27791418 PMC5392813

[27] Fountain DM, Kolias AG, Lecky FE, et al Survival trends after surgery for Acute Subdural Hematoma in adults over a 20-year period. Ann Surg 2017;265:590–6. 10.1097/SLA.0000000000001682 27172128 PMC5300032

[28] Utomo WK, Gabbe BJ, Simpson PM, et al Predictors of in-hospital mortality and 6-month functional outcomes in older adults after moderate to severe traumatic brain injury. Injury 2009;40:973–7. 10.1016/j.injury.2009.05.034 19540490

[29] Faul M, Wald MM, Rutland-Brown W, et al Using a cost-benefit analysis to estimate outcomes of a clinical treatment guideline: testing theBrain Trauma Foundation guidelines for the treatment of severe traumatic brain injury. J Trauma 2007;63:1271–8. 10.1097/TA.0b013e3181493080 18212649

[30] Lee JC, Rittenhouse K, Bupp K, et al An analysis of Brain Trauma Foundation traumatic brain injury guideline compliance and patient outcome. Injury 2015;46:854–8. 10.1016/j.injury.2014.12.023 25661105

[31] Foks KA, van den Brand CL, Lingsma HF, et al External validation of computed tomography decision rules for minor head injury: prospective, multicentre cohort study in the Netherlands. BMJ 2018;362:k3527 10.1136/bmj.k3527 30143521 PMC6108278

[32] Murthy S, Wunsch H Clinical review: International comparisons in critical care - lessons learned. Crit Care 2012;16:218 10.1186/cc11140 22546146 PMC3568952

