# Supplementary material for: Evaluation of the impact of the NICE head injury guidelines on inpatient mortality from traumatic brain injury: an interrupted time series analysis
Source: BMJ Open. 2019 Jun 4;9(6):e028912. doi: 10.1136/bmjopen-2019-028912 (PMC6561604; doi:10.1136/bmjopen-2019-028912)
Supplement: Supplementary data [file bmjopen-2019-028912supp001.pdf]

Supplementary Material 1: Key Features of the NICE Head Injury Guidelines

Policy	Time of Introduction	Key Features
1st NICE Head Injury Guideline	June 2003	Indication for CT imaging (referenced directly from 2003 Guideline):<br><br>- GCS less than 13 on initial assessment in the emergency department.<br>- GCS equal to 13 or 14 at 2 hours after the injury on assessment in the emergency department.<br>- Suspected open or depressed skull fracture.<br>- Any sign of basal skull fracture (haemotympanum, 'panda' eyes, cerebrospinal fluid otorrhoea, Battle's sign).<br>- Post-traumatic seizure.<br>- Focal neurological deficit.<br>- More than one episode of vomiting.<br>- Amnesia for greater than 30 minutes of events before impact.<br><br>CT should also recommended in patients with any of the following risk factors, provided they have experienced some loss of consciousness or amnesia since the injury:<br>-Age greater than or equal to 65 years.<br>-Coagulopathy (history of bleeding, clotting disorder, current treatment with warfarin).<br>-Dangerous mechanism of injury (a pedestrian struck by a motor vehicle, an occupant ejected from a motor vehicle or a fall from a height of greater than 1 metre or five stairs).
2nd NICE Head Injury Guideline	September 2007	Specialist management (referenced directly from 2007 Guideline):<br><br>Local guidelines on the transfer of patients with head injuries should be drawn up between the referring hospital trusts, the neuroscience unit and the local ambulance service, and should recognise that:<br>-Transfer would benefit all patients with serious head injuries (GCS<9), irrespective of the need for neurosurgery

		-If transfer of those who do not require neurosurgery is not possible, ongoing liaison with the neuroscience unit over clinical management is essential.<br><br>Indications Immediate CT scanning (adult):<br>- Glasgow coma score <13 on initial assessment in the emergency department<br>- Glasgow coma score <15 two hours after the injury on assessment in the emergency department<br>- Suspected open or depressed skull fracture<br>- Any sign of basal skull fracture<br>- Post-traumatic seizure<br>- Focal neurological deficit<br>- One or more episodes of vomiting<br>- Amnesia for events more than 30 minutes before impact.<br><br>Indications Immediate CT scanning (<16 years):<br>- Age over 1 year: Glasgow coma score <14 on assessment in the emergency department<br>- Age under 1 year: Glasgow coma score paediatric <15 on assessment in the emergency department<br>- Age under 1 year and presence of bruise, swelling, or laceration (>5 cm) on the head<br>- Clinical suspicion of non-accidental injury<br>- Post-traumatic seizure but no history of epilepsy<br>- Abnormal drowsiness<br>- Suspected open or depressed skull injury, or tense fontanelle<br>- Any sign of basal skull fracture<br>- Focal neurological deficit<br>- Three or more discrete episodes of vomiting<br>- Amnesia (antegrade or retrograde) lasting more than five minutes.
3rd NICE Head Injury Guideline	January 2014	Referenced directly from 3nd NICE Guidelines<br><br>Indications CT scanning < 1 hour (adult):<br>- GCS<13/15<br>- GCS <15 after2 hours from injury<br>- Suspected open or depressed skull fracture<br>- Any sign of basal skull fracture

		- Post-traumatic seizure<br>- Focal neurological deficit<br>- One or more episodes of vomiting<br>Indications CT scanning < 8 hours (adult):<br>- Patient taking warfarin<br>- LOC or amnesia + dangerous mechanism/age 65+/history of bleeding/clotting disorder<br>- Amnesia for events more than 30 minutes before impact.<br><br><br>Indications CT scanning < 1 hour (<16 years) if 1 of:<br>- Suspicion of non-accidental injury<br>- Post-traumatic seizure but no history of epilepsy.<br>- On initial emergency department assessment, GCS less than 14, or for children under 1 year GCS (paediatric) less than 15.<br>- At 2 hours after the injury, GCS less than 15.<br>- Suspected open or depressed skull fracture or tense fontanelle.<br>- Any sign of basal skull fracture (haemotympanum, 'panda' eyes, cerebrospinal fluid leakage from the ear or nose, Battle's sign).<br>- Focal neurological deficit.<br>- For children under 1 year, presence of bruise, swelling or laceration of more than 5 cm on the head.<br>Indications CT scanning < 1 hour (<16 years) if 2 or more of:<br>- Loss of consciousness lasting more than 5 minutes (witnessed).<br>- Abnormal drowsiness.<br>- Three or more discrete episodes of vomiting.<br>- Dangerous mechanism of injury<br>- Amnesia (antegrade or retrograde) lasting more than 5 minutes[4].<br>If only 1 above risk factor observe for 4 hours post injury if during observation develop any risk factor below for CT within 1 hour<br>- GCS less than 15.<br>- Further vomiting.<br>- A further episode of abnormal drowsiness.<br>If taking warfarin for CT within 8 hours.

Supplementary Material 2: Flow diagram of identification of deaths attributable to TBI used in analysis

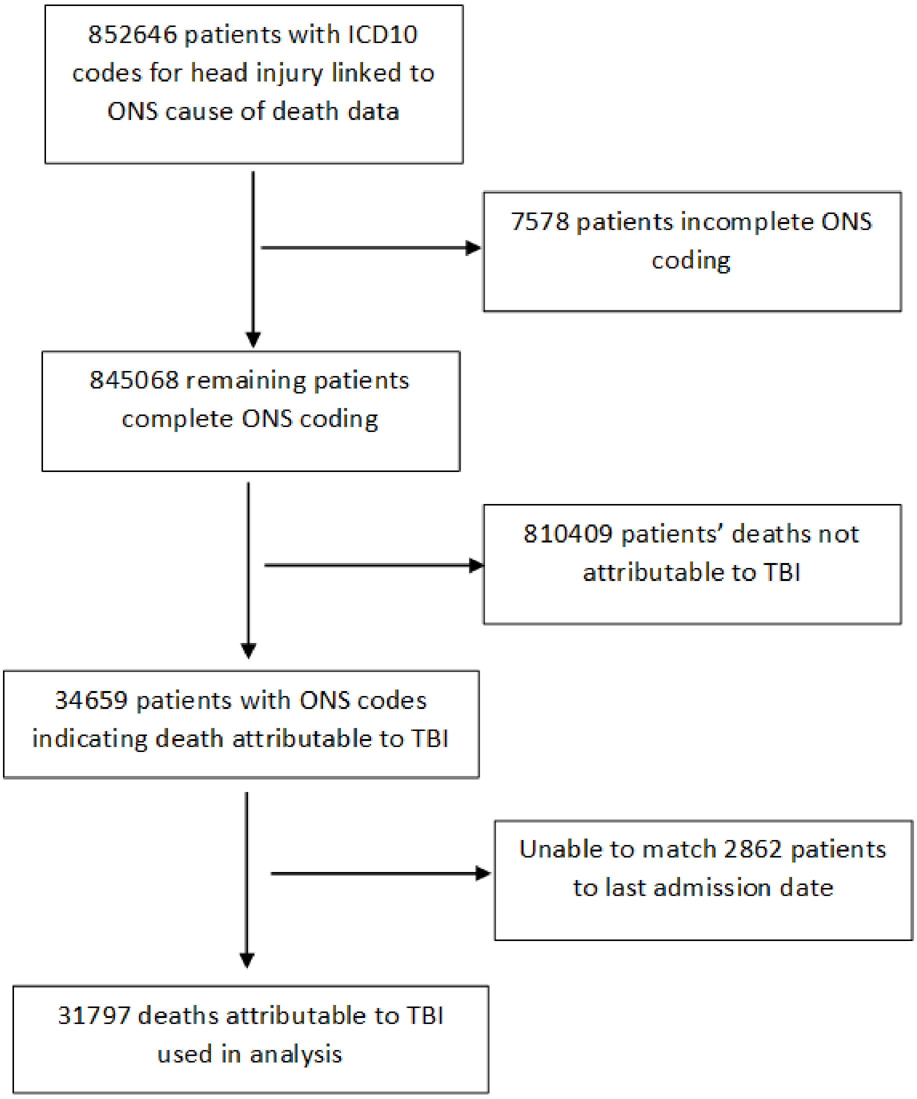

Supplementary Material 3: Annual rate of deaths and admissions for TBI per 100 000 population in England (source NHS digital)

Year	Admissions 0-15	Admissions 16-64	Admissions 65+	Deaths 0-15	Deaths 16-64	Deaths 65+
*1998	177	288	98	0.45	3.96	4.27
1999	238	375	136	0.71	5.75	5.84
2000	218	357	132	0.69	6.32	6.75
2001	213	339	137	0.63	6.62	6.79
2002	198	327	132	0.47	6.44	8.04
2003	199	358	154	0.52	6.57	9.19
2004	207	417	187	0.50	7.12	9.20
2005	208	459	225	0.44	7.55	10.46
2006	201	472	242	0.50	7.57	11.38
2007	185	449	253	0.40	7.68	12.46
2008	177	420	266	0.26	6.84	12.56
2009	183	443	308	0.35	7.18	13.15
2010	181	409	325	0.29	6.19	14.71
2011	185	389	337	0.35	5.73	15.51
2012	162	336	330	0.27	5.80	16.28
2013	156	311	337	0.26	5.34	18.13
2014	151	302	366	0.15	4.84	19.77
2015	131	283	364	0.17	5.08	21.64
2016	125	255	359	0.28	5.17	21.70

*Available data are from April 1998-March 2017, so 1998 is a part year and 2017 is not reported

Supplementary Material 4: Monthly admission characteristics of patients with TBI:

Median age (65 and over)

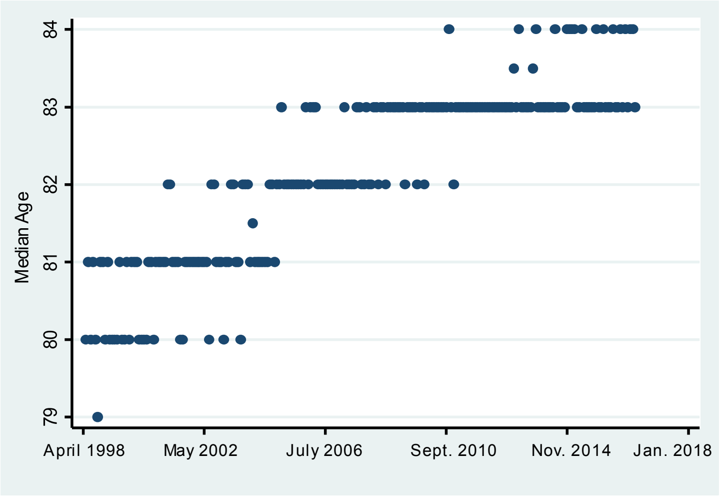

Proportion Male (65 and over)

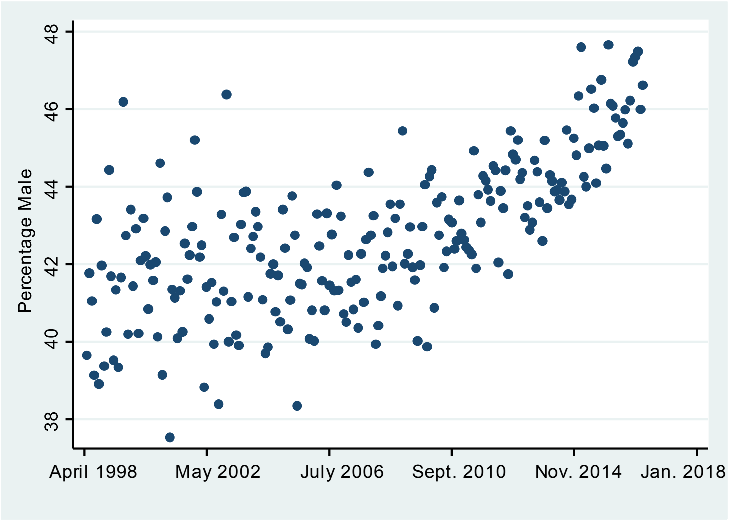

Mean Monthly Charlson Score (65 and over)

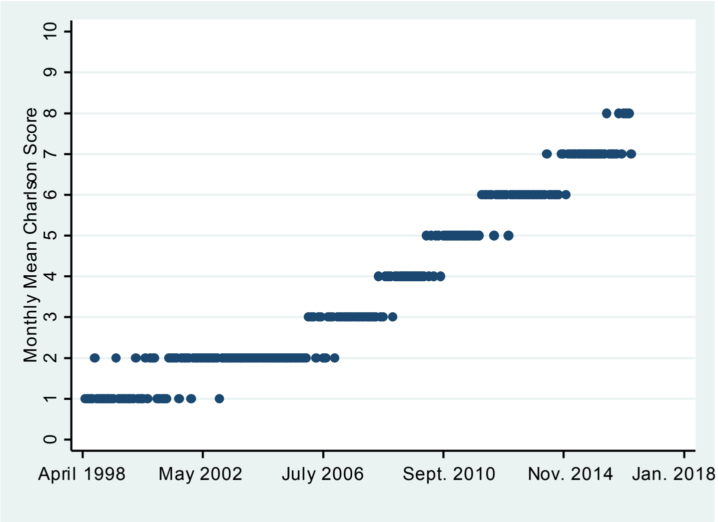

Mean Monthly Trauma Modified Charlson Score (65 and over)

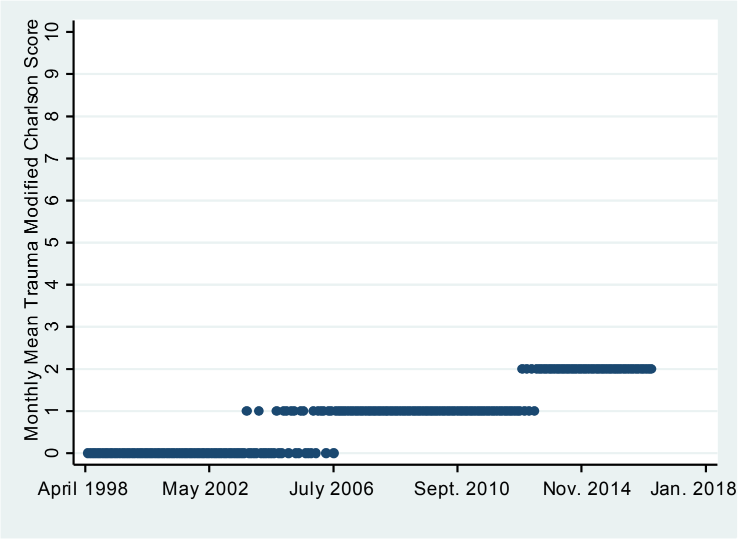

Median age (16-64)

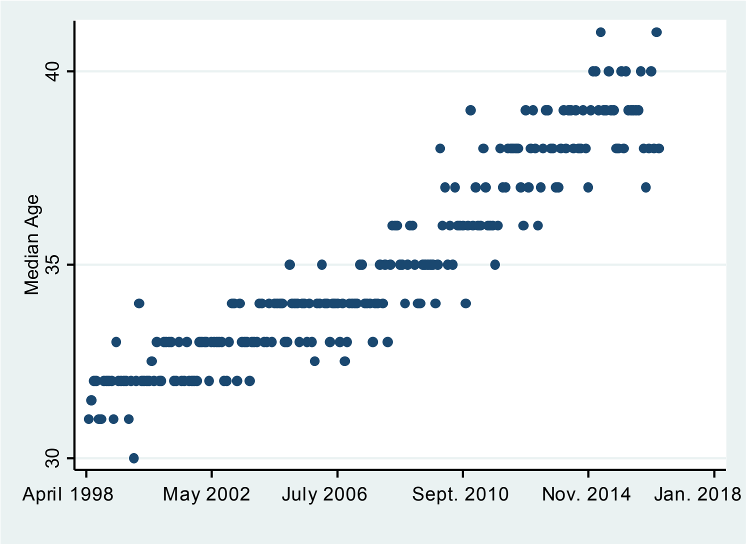

Proportion Male (16-64)

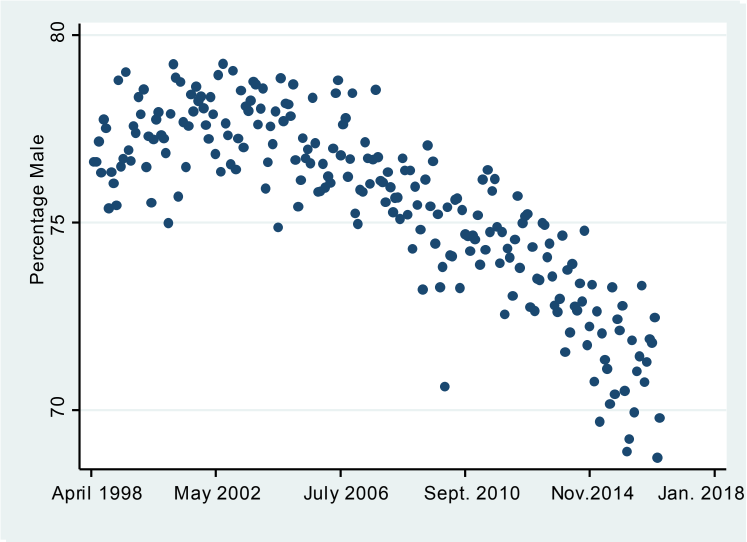

Mean Monthly Standard Charlson Score (16-64)

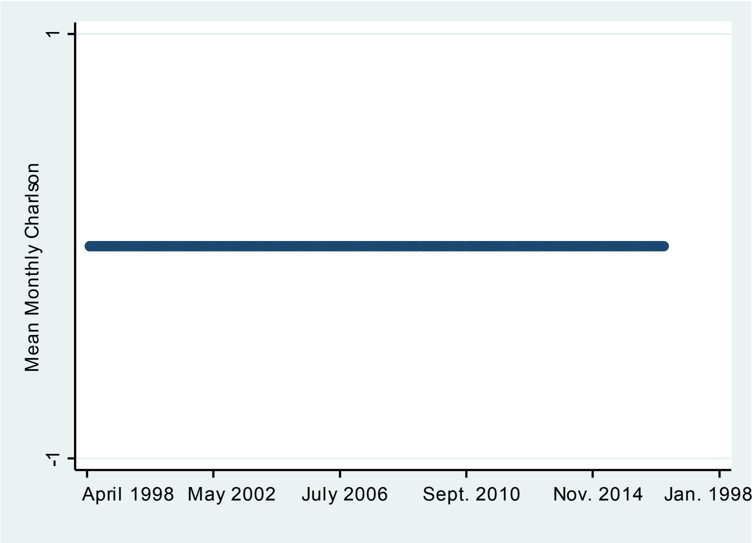

Mean Monthly Trauma Modified Charlson Score (16-64)

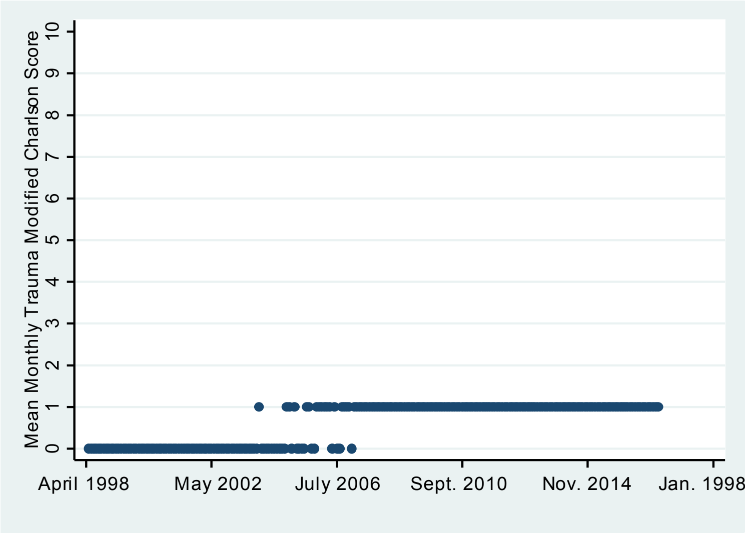

Median age (0-15)

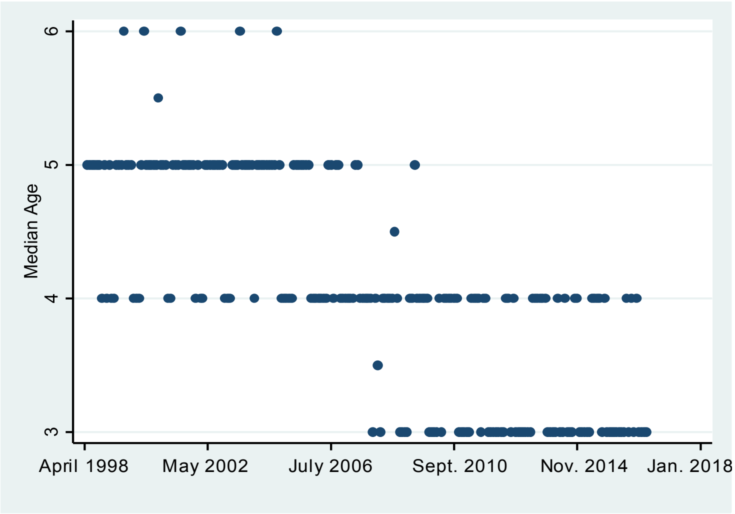

Proportion Male (0-15)

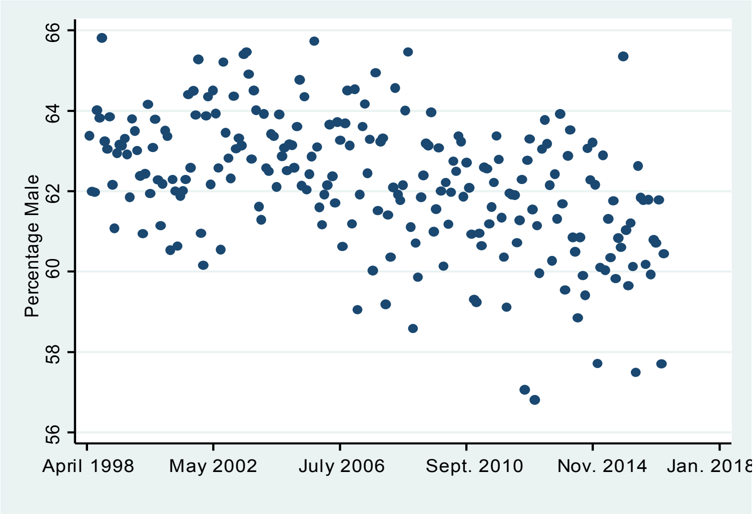

Mean Monthly Standard Charlson Score (0-15)

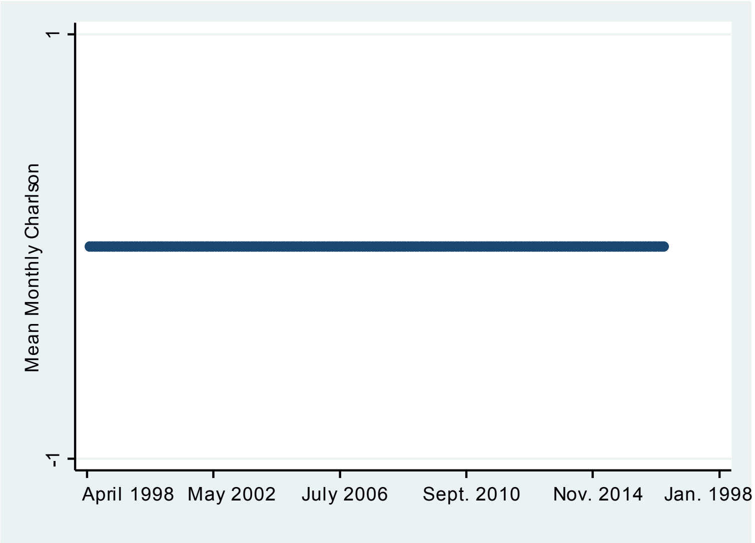

Mean Monthly Trauma Modified Charlson Score (0-15)

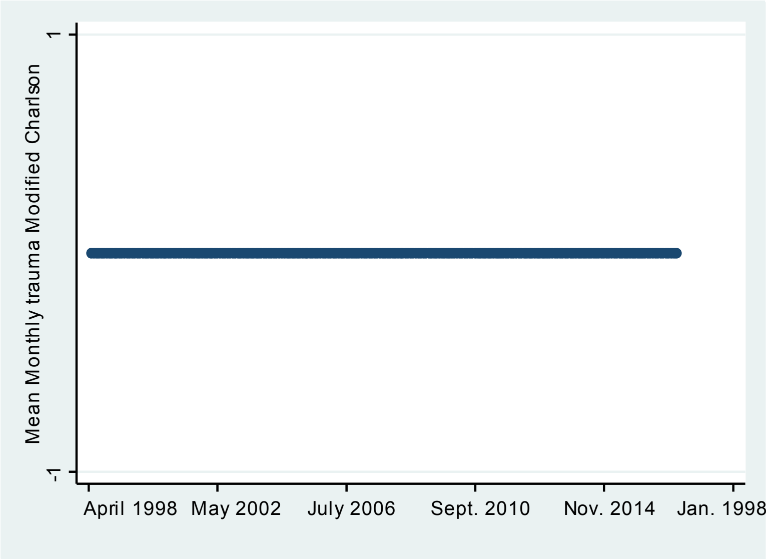

Supplementary Material 5: Subgroup analysis of effect of the NICE guidelines on patients aged 65 deaths per 100, 000 population

**Age Band**	**Winter Effect**	**Initial Trend**	**1st NICE Guideline**	**2nd NICE Guideline**	**3rd NICE Guideline**	**Durbin-Watson Statistic**
**65-84**	-0.06<br>(95% CI: -0.1 to -0.02)<br>P=0.01	0.003<br>(95% CI: 0.001 to 0.005)<br>P=0.006	**Change level:**<br>-0.02<br>(95% CI:-0.13 to 0.1)<br>P=0.78<br>**Change trend:**<br>0.001<br>(95% CI:-0.002 to 0.005)<br>P=0.51	**Change level:**<br>-0.07<br>(95% CI: -0.19 to 0.04)<br>P=0.21<br>**Change trend:**<br>-0.001<br>(95% CI: -0.005 to 0.002)<br>P=0.44	**Change level:**<br>0.09<br>(95% CI:-0.03 to 0.21)<br>P=0.15<br>**Change trend:**<br>-0.003<br>(95% CI:-0.008 to 0.001)<br>P=0.16	Untransformed 1.62<br>Prais-Winsten 1.89
**85+**	-0.46<br>(95% CI: -0.73 to -0.2)<br>P<0.01	0.02<br>(95% CI: 0.01 to 0.03)<br>P=0.01	**Change level:**<br>-0.03<br>(95% CI:-0.7 to 0.7)<br>P=0.92<br>**Change trend:**<br>0.001<br>(95% CI:-0.02 to 0.02)<br>P=0.9	**Change level:**<br>-0.38<br>(95% CI: -1.05 to 0.29)<br>P=0.27<br>**Change trend:**<br>0.02<br>(95% CI: -0.001 to 0.04)<br>P=0.65	**Change level:**<br>0.54<br>(95% CI:-0.18 to 1.26)<br>P=0.14<br>**Change trend:**<br>-0.02<br>(95% CI:-0.05 to 0.01)<br>P=0.15	Untransformed 1.68<br>Prais-Winsten 1.91

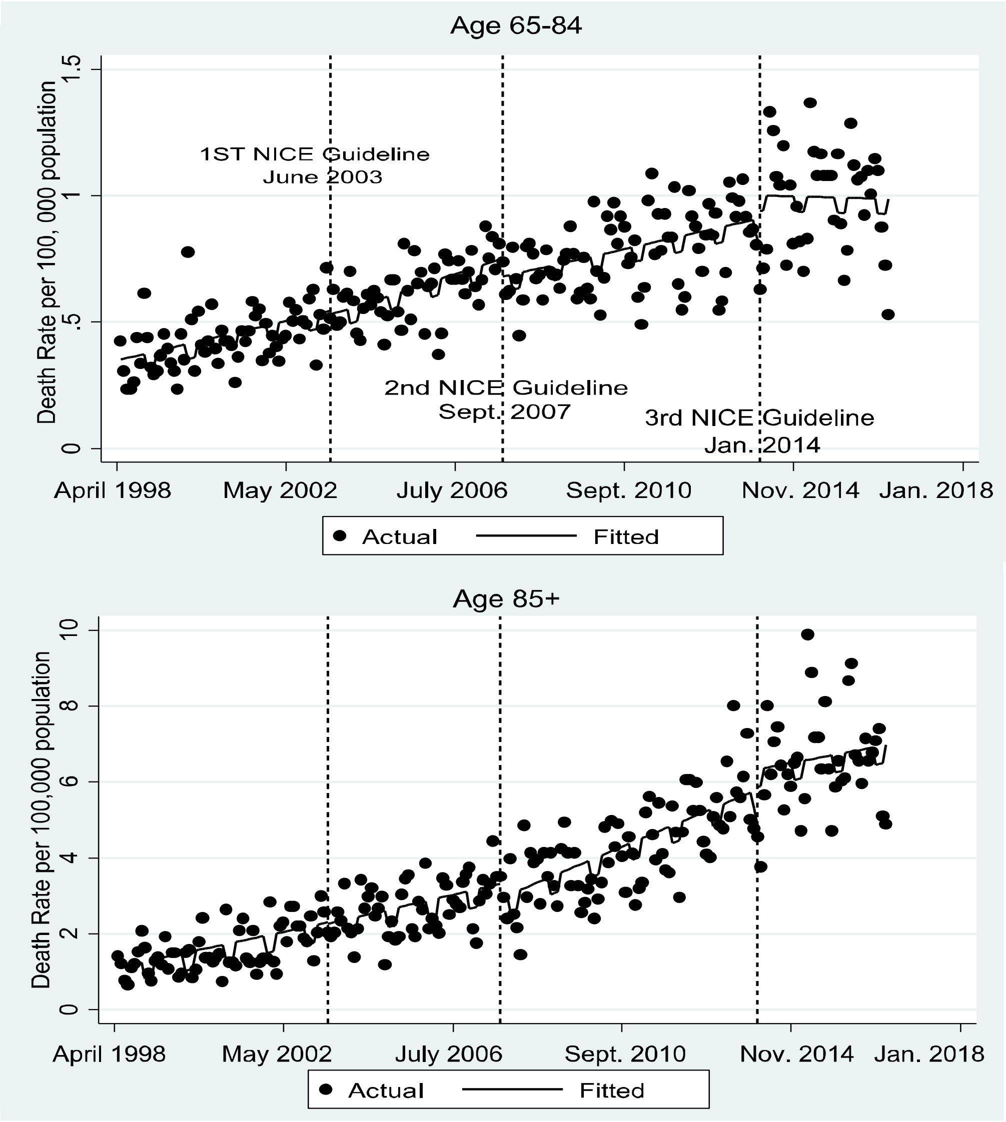
Age 65-84
Death Rate per 100, 000 population
0
.5
1
1.5
1ST NICE Guideline
June 2003
2nd NICE Guideline
Sept. 2007
3rd NICE Guideline
Jan. 2014
April 1998
May 2002
July 2006
Sept. 2010
Nov. 2014
Jan. 2018
Actual
Fitted
Age 85+
Death Rate per 100,000 population
0
2
4
6
8
10
April 1998
May 2002
July 2006
Sept. 2010
Nov. 2014
Jan. 2018
Actual
Fitted

Supplementary Material 6: The impact of the NICE head injury guidelines on monthly TBI mortality rate per 100 000 population adjusted for age, sex and comorbidity

**Age Band**	**Winter Effect**	**Initial Trend**	**Median Age**	**Proportion Male**	**Charlson Score**	**1st NICE Guideline**	**2nd NICE Guideline**	**3rd NICE Guideline**	**Durbin-Watson Statistic**
**65+**	-0.1<br>(95% CI: -0.17 to -0.04)<br>P<0.01	0.006<br>(95% CI: 0.002 to 0.009)<br>P<0.01	-0.03<br>(95% CI: -0.09 to 0.02)<br>P=0.25	0.03<br>(95% CI: -1.80 to 1.87)<br>P=0.97	0.00003<br>(95% CI: -0.07 to 0.07)<br>P>0.99	**Change level:**<br>-0.04<br>(95% CI:-0.22 to 0.14)<br>P=0.69	**Change level:**<br>-0.1<br>(95% CI: -0.28 to 0.07)<br>P=0.25	**Change level:**<br>0.14<br>(95% CI:-0.05 to 0.32)<br>P=0.15	Untransformed 1.56<br>Prais-Winsten 1.86
						**Change trend:**<br>0.003<br>(95% CI: -0.003 to 0.008)<br>P=0.39	**Change trend:**<br>-0.0002<br>(95% CI:-0.006 to 0.005)<br>P=0.95	**Change trend:**<br>-0.005<br>(95% CI:-0.01 to 0.002)<br>P=0.14	
**16-64**	-0.12<br>(95% CI: -0.15 to -0.09)<br>P<0.01	0.001<br>(95% CI: -0.0003 to 0.003)<br>P=0.1	0.03<br>(95% CI: 0.01 to 0.05)<br>P<0.01	1.40<br>(95% CI:0.1 to 2.69)<br>P=0.04	Not adjusted for as no change over time period.	**Change level:**<br>-0.03<br>(95% CI:-0.11 to 0.06)<br>P=0.52	**Change level:**<br>0.06<br>(95% CI:-0.14 to 0.02)<br>P=0.15	**Change level:**<br>-0.0004<br>(95% CI: -0.085 to 0.085)<br>P=0.99	Untransformed 1.89<br>Prais-Winsten 1.98
						**Change trend:**<br>0.0001<br>(95% CI: -0.002 to 0.004)<br>P=0.52	**Change trend:**<br>*-0.006*<br>*(95% CI: -0.008 to -0.003)*<br>*P<0.01*	**Change trend:**<br>0.003<br>(95% CI: 0.00005 to 0.007)<br>P=0.047	
**0-15**	-0.01<br>(95% CI: -0.01 to 0.001)<br>P=0.09	-0.0002<br>(95% CI: -0.0005 to -0.00002)<br>P=0.04	0.006<br>(95% CI: 0.00002 to 0.01) P=0.049	-0.09<br>(95% CI:-0.28 to 0.09)<br>P=0.32	Not adjusted for as no change over time period.	**Change level:**<br>0.0001<br>(95% CI: -0.01 to 0.01) P= 0.99	**Change level:**<br>-0.0004<br>(95% CI: -0.01 to 0.01) P=0.95	**Change level:**<br>-0.01<br>(95% CI:-0.03 to 0.001) P=0.08	Untransformed 2.19<br>Prais-Winsten 1.99
						**Change trend:**<br>0.0001<br>(95% CI:-0.0003 to 0.0005) P=0.58	**Change trend**<br>0.00005<br>(95% CI:-0.0003 to 0.0004)<br>P=0.81	**Change trend:**<br>0.0004<br>(95% CI: -0.00007 to 0.001) P=0.09	

Supplementary Material 7: Sensitivity analysis of implementation lags on the impact of the NICE head injury guidelines on deaths per 100 000 population

Age Band	Winter Effect	Initial Trend	1st NICE Guideline	2nd NICE Guideline	3rd NICE Guideline	Durbin-Watson Statistic
**65+**	-0.11<br>(95% CI: -0.18 to-0.04)<br>P<0.01	0.005<br>(95% CI: 0.001 to 0.008)<br>P<0.01	**Change level:**<br>-0.007<br>(95% CI:-0.2 to 0.19)<br>P=0.95<br>**Change trend:**<br>0.005<br>(95% CI:-0.003 to 0.012)<br>P=0.24	**Change level:**<br>-0.05<br>(95% CI: -0.25 to 0.14)<br>P=0.60<br>**Change trend:**<br>-0.0018<br>(95% CI: -0.01 to 0.006)<br>P=0.65	**Change level:**<br>0.13<br>(95% CI:-0.06 to 0.33)<br>P=0.18<br>**Change trend:**<br>-0.006<br>(95% CI:-0.01 to 0.002)<br>P=0.16	Untransformed 1.56<br>Prais-Winsten 1.86
**16-64**	-0.1<br>(95% CI: -0.14 to -0.06)<br>P<0.01	0.002<br>(95% CI:0.001 to 0.004)<br>P<0.01	**Change level:**<br>0.01<br>(95% CI: -0.08 to 0.11)<br>P=0.78<br>**Change trend:**<br>-0.001<br>(95% CI: -0.004 to 0.003)<br>P=0.77	**Change level:**<br>0.06<br>(95% CI:-0.15 to 0.003)<br>P=0.11<br>**Change trend:**<br>-0.004<br>(95% CI:-0.008 to -0.001)<br>P=0.03	**Change level:**<br>0.006<br>(95% CI: -0.09 to 0.1)<br>P=0.91<br>**Change trend:**<br>0.002<br>(95% CI:-0.002 to 0.005)<br>P=0.41	Untransformed 1.75<br>Prais-Winsten 1.94
**0-15**	-0.01<br>(95%CI:-0.01 to -0.001)<br>P=0.02	-0.0003<br>(95% CI: -0.0005 to -0.00001)<br>P=0.03	**Change level:**<br>0.001<br>(95% CI: -0.01 to 0.01)<br>P= 0.88<br>**Change trend:**<br>0.00007<br>(95% CI: -0.0006 to 0.0005)<br>P=0.80	**Change level:**<br>-0.001<br>(95% CI: -0.01 to 0.01)<br>P=0.93<br>**Change trend**<br>0.0002<br>(95% CI: -0.0003 to 0.0007)<br>P=0.47	**Change level:**<br>-0.01<br>(95% CI:-0.03 to 0.002)<br>P=0.097<br>**Change trend:**<br>0.0005<br>(95% CI: -0.00003 to 0.001)<br>P=0.07	Untransformed 2.18<br>Prais-Winsten 1.98

Supplementary Material 8: Sensitivity analysis of implementation lags on the impact of the NICE head injury guidelines on admissions per 100 000 population

Age Band	Winter Effect	Initial Trend	1st NICE Guideline	2nd NICE Guideline	3rd NICE Guideline	Durbin-Watson Statistic
**65+**	-0.51<br>(95% CI: -1.05 to 0.04)<br>P=0.07	0.02<br>(95% CI -0.02 to 0.05)<br>P=0.31	**Change level:**<br>3.88<br>(95% CI: 2.11 to 5.66)<br>P<0.01	**Change level:**<br>1.71<br>(95% CI: -0.08 to 3.5)<br>P=0.06	**Change level:**<br>0.6<br>(95% CI:-1.17 to 2.36)<br>P=0.51	Untransformed 1.24<br>Prais-Winsten 2.05
			**Change trend:**<br>0.17<br>(95% CI: 0.09 to 0.24)<br>P<0.01	**Change trend:**<br>-0.1<br>(95% CI: -0.17 to -0.03)<br>P=0.01	**Change trend:**<br>-0.1<br>(95% CI:-0.18 to -0.03)<br>P=0.01	
**16-64**	-2.16<br>(95% CI: -3.03 to -1.28)<br>P<0.01	-0.08<br>(95% CI:-0.12 to -0.03)<br>P<0.01	**Change level:**<br>8.6<br>(95% CI: 6 to 11.2)<br>P<0.01	**Change level:**<br>-2.22<br>(95% CI:-4.84 to 0.4)<br>P=0.1	**Change level:**<br>0.25<br>(95% CI:-2.33 to 2.84)<br>P=0.85	Untransformed 1.49<br>Prais-Winsten 2.06
			**Change trend:**<br>0.2<br>(95% CI: 0.09 to 0.3)<br>P<0.01	**Change trend:**<br>-0.32<br>(95% CI: -0.42 to -0.21)<br>P<0.01	**Change trend:**<br>0.06<br>(95% CI:-0.05 to 0.16)<br>P=0.29	
**0-15**	-2.93<br>(95% CI: -3.49 to -2.38)<br>P<0.01	-0.06<br>(95%CI:-0.11 to -0.01)<br>P=0.02	**Change level:**<br>1.16<br>(95% CI: -1.22 to 3.54)<br>P= 0.34	**Change level:**<br>0.4<br>(95% CI: -1.99 to 2.8)<br>P=0.74	**Change level:**<br>0.5<br>(95% CI:-1.87 to 2.88)<br>P=0.68	Untransformed 1.06<br>Prais-Winsten 1.71
			**Change trend:**<br>0.02<br>(95% CI: -0.1 to 0.13)<br>P=0.8	**Change trend**<br>-0.01<br>(95% CI: -0.12 to 0.1)<br>P=0.9	**Change trend:**<br>-0.06<br>(95% CI: -0.17 to 0.05)<br>P=0.28	

Supplementary Material 9: Annual attendance to the ED in England for head injury

Year	Number head injury primary diagnosis for ED attendance	Proportion attendances primary diagnosis head injury (all attendances)	Proportion attendances primary diagnosis head injury (where primary diagnosis known)
2007/2008	238,099	1.90%	
2008/2009	272,485	2.00%	
2009/2010	336,396	2.2%	3.7%
2010/2011	363,187	2.2%	3.8%
2011/2012	421,221	2.4%	3.8%
2012/2013	423,413	*2.3%*	*3.7%*
2013/2014	449,397	*2.4%*	3.8%
2014/2015	395, 401	*2%*	3.1%
2015/2016	430, 725	*2.1%*	3.2%
2016/2017	449, 584	*2.2%*	3.3%
2017/2018	443, 758	*2.1%*	3.0%

*data obtained from NHS Digital Annual ED reports https://digital.nhs.uk/data-and-information/publications/statistical/hospital-accident--emergency-activity (data was submitted by all hospitals in England from 2012 onwards, prior to this data was only submitted by a variable proportion of hospitals)
