# Supplementary material for: Evaluation of the impact of the NICE head injury guidelines on inpatient mortality from traumatic brain injury: an interrupted time series analysis
Source: BMJ Open. 2019 Jun 4;9(6):e028912. doi: 10.1136/bmjopen-2019-028912 (PMC6561604; doi:10.1136/bmjopen-2019-028912)
Supplement: Author's manuscript [file bmjopen-2019-028912.draft_revisions.pdf]

BMJ Open

BMJ Open is committed to open peer review. As part of this commitment we make the peer review history of every article we publish publicly available.

When an article is published we post the peer reviewers' comments and the authors' responses online. We also post the versions of the paper that were used during peer review. These are the versions that the peer review comments apply to.

The versions of the paper that follow are the versions that were submitted during the peer review process. They are not the versions of record or the final published versions. They should not be cited or distributed as the published version of this manuscript.

BMJ Open is an open access journal and the full, final, typeset and author-corrected version of record of the manuscript is available on our site with no access controls, subscription charges or pay-per-view fees (http://bmjopen.bmj.com).

If you have any questions on BMJ Open's open peer review process please email info.bmjopen@bmj.com

BMJ Open

The effect of the NICE head injury guidelines on inpatient mortality from traumatic brain injury: an interrupted time series analysis

Journal:	*BMJ Open*
Manuscript ID	bmjopen-2019-028912
Article Type:	Research
Date Submitted by the Author:	02-Jan-2019

Complete List of Authors:	Marincowitz, Carl; University of Hull, Hull York Medical School<br>Lecky, Fiona; University of Sheffield, School of Health and Related Research<br>Allgar, Victoria; York University, HYMS/Health Sciences<br>Sheldon, Trevor; University of York, health Sciences

Keywords:	ACCIDENT & EMERGENCY MEDICINE, Health policy < HEALTH SERVICES ADMINISTRATION & MANAGEMENT, Traumatic Brain Injury, Head Injury

SCHOLARONE™ Manuscripts

The effect of the NICE head injury guidelines on inpatient mortality from traumatic brain injury: an interrupted time series analysis

Carl Marincowitz[1] NIHR Doctoral Research Fellow, MB BChir, MSc, BA

Fiona E. Lecky[2] Professor, MB Ch B, FRCS, DA, MSc, PhD, FCEM

Victoria Allgar[3] Senior Statistician, PhD, Chartered Statistician, Royal Statistical Society, BSc

Trevor A. Sheldon[4] Professor, MSc, MSc, DSc, FMedSci

1. **Corresponding Author.** Hull York Medical School, Allam Medical Building, University of Hull, Hull HU6, 7RX, Fax: +44 (0) 1482 464705 Tel +44 (0) 870 1245500
Email: Carl.Marincowitz@hyms.ac.uk

2. School of Health and Related Research, University of Sheffield, Regent Court, 30 Regent Street, Sheffield, S1 4DA, UK, Fax: +44 (0)114 222 0749 Tel: (+44) (0)114 222 4345,
Email: f.e.lecky@sheffield.ac.uk

3 Hull York Medical School, John Hughlings Jackson Building, University of York, Heslington, York YO10 5DD Tel: 0870 124 5500, Email: Victoria.Allgar@hyms.ac.uk

4. Department of Health Sciences, University of York, Alcuin Research Resource Centre, Heslington, York, YO10 5DD, Tel +44 (0) 1904 321344, Fax: +44 (0) 1904 32 3433,
E-mail: Trevor.Sheldon@york.ac.uk

Word Count: 3476

For peer review only - http://bmjopen.bmj.com/site/about/guidelines.xhtml

For peer review only

Abstract

Objective

To evaluate the impact of National Institute of Health and Care Excellence (NICE) head injury guidelines on deaths and hospital admissions caused by traumatic brain injury (TBI).

Setting

All hospitals in England between 1998-2017.

Participants

Patients admitted to hospital or who died up to 30 days following hospital admission with ICD coding indicating the reason for admission or death was TBI.

Intervention

An interrupted time series analysis was conducted with intervention points when each guideline was introduced. Analysis was stratified by guideline recommendation specific age groups (0-15, 16-64 and 65+).

Outcome Measures

The monthly population mortality and admission rate for TBI.

Study Design

An interrupted time series analysis using complete Office of National Statistics (ONS) cause of death data linked to Hospital Episode Statistics for inpatient admissions in England.

Results

The monthly TBI mortality and admission rate in the 65+ age group increased from 0.5 to 1.5 and 10 to 30 per 100, 000 population respectively. The increasing mortality rate was unaffected by the introduction of any of the guidelines.

The introduction of the 2nd NICE Head Injury guideline was associated with a significant reduction in the monthly TBI mortality rate in the 16-64 age group (-0.005; 95% CI:-0.002 to -0.007).

For peer review only - http://bmjopen.bmj.com/site/about/guidelines.xhtml

In the 0-15 age group the TBI mortality rate fell from around 0.05 to 0.01 per 100 000 population, the trend was unaffected by the guidelines.

Conclusion

The introduction of NICE head injury guidelines was associated with reduced admitted TBI mortality rates after specialist care was recommended for severe TBI. The improvement was solely observed in 16-64 year olds.

The cause of the observed increased admission and mortality rate in those 65+ and potential treatments for TBI in this age group require further investigation.

Strengths and Limitations of this study:

This study is the first to use complete national data and interrupted time series analysis to evaluate the impact of the NICE head injury guidelines.

Using the robust method of interrupted time series analysis, we found the sole TBI mortality change attributable to guideline introduction occurred in 16-64 year olds between 2007-14 - after the publication of CG 56.

Inpatient mortality was assessed at a population level as national data on ED attendance for TBI was unavailable and the guidelines acted to change the admission threshold for TBI identified by CT imaging.

Keywords:

Traumatic Brain Injury, Head Injury, NICE Guidelines, Health Service Evaluation.

For peer review only

Background

There are approximately 2.5 million cases of Traumatic Brain Injury (TBI) (injury to the brain/ functional impairment due to external force) annually in the European Union and TBI is a leading cause of death and disability.[1] In higher income countries the epidemiology of TBI has changed from a condition predominantly of younger males resulting from high energy trauma, to older people caused by falls.[2]

One of the important health service challenges is identifying the small proportion of patients with life threatening TBI amongst the large number of patients who attend Emergency Departments (EDs) following head injury (blunt trauma to the head) and then ensure they receive specialist care, including neurosurgery, within a time critical period.[3] Previous research demonstrated correctly configured emergency health care systems are required to deliver optimal outcomes for patients with severe TBI.[1 4]

In England, since 2003, three NICE head injury guidelines have aimed to improve the ED identification and subsequent management of TBI (Supplementary Material 1).[3 5-7] All three guidelines advocated increased CT imaging of head injured patients that present with a minimally impaired conscious level equivalent to a Glasgow Coma Scale (GCS) of 13-15. Increased costs from imaging were intended to be offset through reduced hospital admissions.[8] The 2007 guideline additionally recommended that patients with severe TBI should be managed in specialist neuroscience centres. At the time of implementation, concerns were raised that guideline recommendations were based on studies in subgroups and lacked supporting level 1 evidence .[4 9 10] Evaluation of the effect of these guidelines on national rates of TBI admissions and patient outcomes, is therefore needed.

We describe the first study to use complete national data and interrupted time series analysis to evaluate the impact of early TBI management guidelines on patient outcomes and admission rates for all severities of TBI.

For peer review only - http://bmjopen.bmj.com/site/about/guidelines.xhtml

Methods

Data set:

Hospital Episode Statistics (HES) are collected on all inpatients in England. The Office for National Statistics (ONS) has computerised ICD coding of cause of death information recorded on death certificates.

We used individual patient level HES data provided by NHS Digital on all emergency inpatient hospital admissions in England from April 1998 to April 2017. Reason for admission is recorded using ICD10 coding. For patients with ICD10 diagnostic codes: S00-S09 (indicating TBI) or T04.0 and T06.0 (crushing injury to the head) who died up to 30 days from discharge ONS cause of death was also provided.[11] ONS coding changed from ICD9 to ICD10 in 2001.

Deaths attributable to TBI:

Supplementary Material 2 summarises how deaths attributable to TBI over the study period were identified. 852646 deaths linked to admissions for head injury were identified by NHS Digital. We searched all cause of death fields for ICD9 and ICD10 codes defined by the CDC as indicating a death attributable to TBI (Table 1).[12] When any were present the death was coded as attributable to TBI. 34659 deaths attributable to TBI were identified and these were linked to their last recorded admission date as a proxy for when the injury and death occurred. This was not possible for 2862 patients. Neonatal deaths were excluded from analysis due to differences in cause of death coding.

For peer review only - http://bmjopen.bmj.com/site/about/guidelines.xhtml

Table 1: Annual numbers of deaths and admissions from TBI in England (source NHS digital)

Year	Admissions all age groups	Admissions 0-15	Admissions 16-64	Admissions 65+	Death all age groups	Deaths 0-15	Deaths 16-64	Deaths 65+
*1998	47820	17739	22348	7631	677	45	307	331
1999	63599	23848	29088	10553	964	71	446	453
2000	60001	21774	27793	10280	1076	69	492	525
2001	58497	21065	26553	10774	1105	62	519	532
2002	55941	19579	25808	10424	1178	46	508	634
2003	60336	19630	28405	12239	1294	51	521	729
2004	68662	20361	33298	14937	1342	49	568	734
2005	75391	20417	36832	18093	1484	43	606	840
2006	77333	19696	38005	19566	1570	49	610	917
2007	75219	18128	36473	20566	1665	39	624	1012
2008	74158	17481	34657	21938	1621	26	564	1036
2009	81218	18111	37178	25848	1739	35	603	1105
2010	81032	18008	35064	27856	1817	29	530	1260
2011	82093	18604	33989	29390	1879	35	500	1354
2012	76925	16453	30475	29901	2025	27	525	1474
2013	76429	15966	28983	31379	2204	27	497	1687
2014	79372	15535	28833	34890	2361	15	462	1886
2015	76648	13630	27517	35357	2610	18	493	2102
2016	74242	13120	25228	35488	2682	30	511	2145
*2017	16247	2619	5483	8037	504		79	420

*Data are from April 1998-March 2017, so 1998 and 2017 are part years and small number have been suppressed in accordance with NHS Digital guidance

ICD9 definition TBI: 800, 801, 803, 804, 850, 851, 852, 853, 854, 905.0, 907.0 and 873

ICD10 definition TBI: S01.0–S01.9, S02.0, S02.1, S02.3, S02.7-S02.9, S04.0, S06.0–S06.9, S07.0, S07.1, S07.8, S07.9, S09.7–S09.9, T01.0, T02.0, T04.0, T06.0, T90.1, T90.2, T90.4, T90.5, T90.8 andT90.9

For peer review only - http://bmjopen.bmj.com/site/about/guidelines.xhtml

Admissions attributable to TBI:

The same ICD10 codes used were used to identify patients admitted with TBI (Table 1).[12] We searched the primary diagnostic field in the inpatient HES data set for these codes and when present the reason for admission was coded as due to TBI. Data were cleaned and continuous inpatient spells (CIPS) were created for patients admitted with TBI using the approach outlined by Castelli, Laudicella and Street as this includes transfers within CIPS.[13] 1361537 CIPS for TBI were identified for 1245720 patients. Following cleaning, 402 CIPs were found to have admission dates prior to April 1998 and were excluded. Demographic and comorbidity information was calculated from the first consultant episode of a CIP. This included the monthly proportion of TBI admissions for males, monthly median age of admissions and mean monthly admission Charlson Comorbidity Index Score (using ICD10 code definitions and weights used to calculate the Summary Hospital-level Mortality Indicator (SHMI)).[14] This was compared to adjustment using a modified Charlson Comorbidity Index derived from the national (Trauma Audit and Research Network - TARN) trauma registry.[15]

Outcomes:

The monthly number of patients with deaths and admissions attributable to TBI between April 1998 and March 2017 was calculated. These were stratified into guideline specific age groupings: 0-15, 16-64 and ≥65. Monthly mortality and admission rates were calculated per 100, 000 population using Nomis ONS mid-year population estimates for England for each age grouping.[16]

Statistical Analysis:

A monthly time series of the mortality rate for TBI was plotted for the study period. Interrupted times series analysis (ITS) was conducted assessing the impact of the NICE guidelines using the ITSA package in STATA 14.[17] ITS analysis is a robust and increasingly used quasi-experimental method for the evaluation of health policies and allows causality to be attributed to an intervention introduced at a specific time point.[18]

The ITS model included three intervention time points corresponding to the introduction of each guidelines in: June 2003, September 2007 and January 2014. Analysis was conducted

For peer review only - http://bmjopen.bmj.com/site/about/guidelines.xhtml

separately for the 0-15, 16-64 and ≥65s age groups. A segmented regression model predicting the mortality rate for TBI per 100, 000 population in each age grouping per month was estimated.[18] The segmented regression model was then adjusted for the monthly proportion of males, median age and mean Charlson Comorbidity Index Score.

A monthly time series was plotted and the same analysis repeated for the monthly rate of admissions for TBI per 100, 000 population, also stratified into the 3 age groups. No adjustment for age, sex or comorbidity was completed.

In all analyses, autocorrelation of the residuals was assessed using the Durbin-Watson and Rho statistic. Throughout we used the Prais-Winsten transformation adjustment for auto-correlation due to improved fit of the model, deviation from a Durbin Watson statistic of 2 and a non-statistically significant Rho statistic.[18] Seasonality was assessed by introducing a dummy variable to the model in which winter months (December, January and February) were coded 1 and was included in the model when statistically significant.[19] To assess for the effect of implementation lags a sensitivity analysis was performed for all models in which the 12 months immediately following the introduction of a guideline were removed.[18]

Ethics

This study involved the analysis of anonymised routinely collected data and therefore NHS Research Ethics Committee review was not required. Data were stored and processed in accordance with NHS Digital guidance and data sharing agreement.

Patient and Public Involvement

The Hull and East Yorkshire NHS Trust Trans-Humber Consumer Research Panel and Hull branch of the Headway charity were consulted in the initial stages of developing the research questions addressed in this study. These patient groups highlighted that although national head injury guidelines seemed evidence based, there appeared to be little evidence to show they had achieved their aims.

Results

Mortality rate:

For peer review only - http://bmjopen.bmj.com/site/about/guidelines.xhtml

Table 1 shows the annual number of deaths and hospital admissions for TBI. The proportion of all TBI annual admissions and deaths in patients over 65 increased from 17% and 49% in 1998 to 48% and 78% in 2016. Figure 1 shows the monthly mortality rate per 100, 000 population in each age group. Table 2 shows the results of the unadjusted interrupted time series assessing the impact of the NICE head injury guidelines. Deaths were more likely to occur in non-winter months in all age groups and so the figures are seasonally adjusted.

The trends in mortality rate and impact of the guidelines varied between age groups. In the 65+ age group the monthly TBI mortality rate increased from around 0.5 to over 1.5 per 100, 000 population over the time period (Figure 1a). This was accompanied by an increase in the Charlson score of patients 65+ admitted with TBI (Supplementary Material 3). The NICE head injury guidelines were not associated with statistically significant changes in the level or trend in the mortality rate (Table 2). Subgroup analysis of patients aged 65-84 and 85+ showed that the increase in the mortality rate was greater in those 85+, from around 1 to over 6 per 100, 000 population but similar estimates of guideline effect to the whole 65+ population (Supplementary Material 4).

The 2nd guideline was found to be associated with a large reduction in mortality in the 16-64 age group. Before the guideline, the monthly mortality rate was increasing but the introduction of the 2nd NICE guideline is associated with a reversal of this trend (-0.005; 95% CI:-0.002 to -0.007) (Table 2). The reduction in mortality appears to slow at the time of the introduction of the 3rd NICE guideline but this was not statistically significant. There was an increase in age of patients in the 16-64 age group admitted with TBI but no change in the Charlson comorbidity score over the period (Supplementary Material 3).

In the 0-15 age group the mortality rate fell continuously over the time period from around 0.05 to 0.01 per 100 000 population. There were fewer monthly numbers of deaths and so more random variability in rates. None of the guidelines were associated with a statistically significant change in the level or trend in the mortality rate (Table 2), though the high random variability meant we had lower statistical power to detect such changes as statistically significant.

Adjustment for the monthly median age, mean Charlson Score and proportion of male admissions for TBI did not materially alter the estimates of guideline effect in any of the age

For peer review only - http://bmjopen.bmj.com/site/about/guidelines.xhtml

groups (Supplementary Material 5). In the 16-64 age group the estimate of the reversal in trend in mortality rate associated with the 2nd Guideline, -0.006 (95% CI:-0.008 to -0.003), was similar to the unadjusted analysis. The levelling off in the rate of reduction in mortality in the 16-64 age group associated with the 3rd NICE guideline became marginally statistically significant, although the estimate of effect is similar, 0.003 (95% CI: 0.00005 to 0.007). No adjustment was made for the standard Charlson score in the paediatric and 16-64 age groups as it did not change over time. The monthly mean trauma modified Charlson score in the 16-64 age group increased slightly from 0 to 1 and adjustment for this increased the estimate of the 2nd NICE guideline's effect, -0.008 (95% CI:-0.01 to -0.005), (Supplementary Material 3). The sensitivity analysis for the effect of implementation lags did not affect the estimates of guideline effects (Supplementary Material 6).

Admission Rate:

Figure 2 shows the trends in monthly TBI admissions stratified by age group and Table 3 presents estimates of the effect of the NICE Head Injury guidelines. The admission rate increased threefold (from around 10 per 100, 000 to 30 per 100, 000) in the 65+ age group. The introduction of the 1st NICE guideline is associated with large increasing trends in monthly TBI admissions per 100,000 population in both the 65+ age group (0.17: 95% CI: 0.11 to 0.22) and the 16-64 age group (0.25: 95% CI: 0.16 to 0.34) (Table 3).[20] The subsequent 2 guidelines are associated with significant reductions in this trend and admission rates level off following the 3rd guideline in the 65+ age group (Table 3 and Figure 2a). In the 16-64 age group, the TBI admissions trend reverses and declines after the 2nd NICE guideline (-0.33: 95% CI: -0.42 to -0.25) (Table 3 and Figure 2b).

In the 0-15 age group TBI admissions steadily fall over the study period from around 20 per 100, 000 to 10 per 100, 000 (Figure 2c), and is unaffected by the introduction of the guidelines (Table 3).

A sensitivity analysis for implementation lags in which the 12 months following the introduction of a guideline were removed from the analysis did not materially change the estimates of effect in any age group (Supplementary Material 7).

For peer review only - http://bmjopen.bmj.com/site/about/guidelines.xhtml

Table 2: The impact of the NICE head injury guidelines on monthly TBI mortality rate per 100 000 population

**Age Band**	**Winter Effect**	**Initial Trend**	**1st NICE Guideline**	**2nd NICE Guideline**	**3rd NICE Guideline**	**Durbin-Watson Statistic**
**65+**	-0.1<br>(95% CI: -0.16 to -0.04)<br>P<0.01	0.005<br>(95% CI: 0.002 to 0.008)<br>P<0.01	**Change level:**<br>-0.034<br>(95% CI:-0.21 to 0.14)<br>P=0.71	**Change level:**<br>-0.1<br>(95% CI: -0.27 to 0.07)<br>P=0.24	**Change level:**<br>0.13<br>(95% CI:-0.04 to 0.32)<br>P=0.14	Untransformed<br>1.57<br>Prais-Winsten<br>1.86
			**Change trend:**<br>0.002<br>(95% CI:-0.003 to 0.008)<br>P=0.43	**Change trend:**<br>0.0004<br>(95% CI: -0.005 to 0.006)<br>P=0.89	**Change trend:**<br>-0.005<br>(95% CI:-0.01 to 0.002)<br>P=0.14	
**16-64**	-0.1<br>(95% CI: -0.13 to -0.06)<br>P<0.01	0.002<br>(95% CI:0.001 to 0.004)<br>P<0.01	**Change level:**<br>-0.03<br>(95% CI: -0.11 to 0.06)<br>P=0.57	**Change level:**<br>-0.06<br>(95% CI:-0.15 to 0.003)<br>P=0.17	**Change level:**<br>0.005<br>(95% CI:-0.087 to 0.096)<br>P=0.92	Untransformed<br>1.79<br>Prais-Winsten<br>1.95
			**Change trend:**<br>-0.00002<br>(95% CI: -0.003 to 0.003)<br>P=0.99	**Change trend:**<br>*-0.005*<br>*(95% CI:-0.007 to -0.002)*<br>*P<0.01*	**Change trend:**<br>0.002<br>(95% CI:-0.002 to 0.005)<br>P=0.38	
**0-15**	-0.01<br>(95% CI:-0.01 to -0.003)<br>P<0.01	-0.0003<br>(95% CI: -0.0005 to -0.00001)<br>P=0.04	**Change level:**<br>0.001<br>(95% CI: -0.01 to 0.01)<br>P= 0.18	**Change level:**<br>-0.0021<br>(95% CI: -0.01 to 0.01)<br>P=0.74	**Change level:**<br>-0.01<br>(95% CI:-0.03 to 0.002)<br>P=0.09	Untransformed<br>2.12<br>Prais-Winsten<br>1.99
			**Change trend:**<br>0.00004<br>(95% CI:-0.0004 to 0.0004)<br>P=0.17	**Change trend**<br>0.0001<br>(95% CI:-0.0003 to 0.0005)<br>P=0.58	**Change trend:**<br>0.0005<br>(95% CI: -0.00005 to 0.001)<br>P=0.08	

For peer review only

For peer review only - http://bmjopen.bmj.com/site/about/guidelines.xhtml

Table 3: The impact of the NICE head injury guidelines on monthly TBI hospital admission rate per 100 000 population

Age Band	Winter Effect	Initial Trend	1st NICE Guideline	2nd NICE Guideline	3rd NICE Guideline	Durbin-Watson Statistic
**65+**	-0.44<br>(95% CI: -0.94 to 0.06)<br>P=0.08	0.01<br>(95% CI: -0.02 to 0.05)<br>P=0.42	***Change level:***<br>*1.71*<br>*(95% CI:-0.01 to 3.44)*<br>*P=0.05*	**Change level:**<br>-0.4<br>(95% CI: -2.08 to 1.27)<br>P=0.64	**Change level:**<br>0.04<br>(95% CI:-1.73 to 1.82)<br>P=0.96	Untransformed 1.1<br>Prais-Winsten 2.09
			Change trend:<br>*0.17*<br>*(95% CI: 0.11 to 0.23)*<br>*P<0.01*	***Change trend:***<br>*-0.08*<br>*(95% CI: -0.13 to -0.03)*<br>*P<0.01*	***Change trend:***<br>*-0.13*<br>*(95% CI:-0.2 to -0.05)*<br>*P<0.01*	
**16-64**	-1.92<br>(95% CI: -2.77 to -1.07)<br>P<0.01	-0.08<br>(95% CI: -0.13 to - 0.02)<br>P<0.01	***Change level:***<br>*5.21*<br>*(95% CI: 2.53 to 7.89)*<br>*P<0.01*	***Change level:***<br>*-2.76*<br>*(95% CI:-5.35 to -0.16)*<br>*P=0.04*	**Change level:**<br>-0.72<br>(95% CI: -3.49 to 2.03)<br>P=0.61	Untransformed 1.35<br>Prais-Winsten 2.11
			Change trend:<br>*0.25*<br>*(95% CI: 0.16 to 0.34)*<br>*P<0.01*	***Change trend:***<br>*-0.33*<br>*(95% CI: -0.42 to -0.25)*<br>*P<0.01*	**Change trend:**<br>0.02<br>(95% CI:-0.09 to 0.13)<br>P=0.73	
**0-15**	-2.87<br>(95% CI: -3.40 to -2.34)<br>P<0.01	-0.06<br>(95% CI:-0.11 to -0.01)<br>P=0.03	**Change level:**<br>1.3<br>(95% CI: -1.03 to 3.63)<br>P= 0.27	**Change level:**<br>0.19<br>(95% CI: -2.09 to 2.47)<br>P=0.87	**Change level:**<br>0.34<br>(95% CI:-2.03 to 2.72)<br>P=0.78	Untransformed 1.07<br>Prais-Winsten 1.70
			**Change trend:**<br>0.02<br>(95% CI: -0.07 to 0.11)<br>P=0.61	**Change trend**<br>-0.005<br>(95% CI: -0.08 to 0.08)<br>P=0.91	**Change trend:**<br>-0.08<br>(95% CI: -0.19 to 0.03)<br>P=0.17	

For peer review only - http://bmjopen.bmj.com/site/about/guidelines.xhtml

Discussion:

Summary:

To our knowledge this is the first study to use national population based data and interrupted time series analysis to evaluate the impact of the NICE head injury guidelines in England. The 2nd NICE guideline was associated with a reduction in the admitted TBI mortality rate in the 16-64 age group at a population level (Table 2). We found no other impact on mortality associated with the three guideline iterations.

There was a continual and significant increase in TBI mortality and admission rates in the 65+ age group and a contrasting falling trend in mortality and admission rates in children. (Figure 1 and Figure 2). Both trends began before the introduction of the NICE guidelines and were not significantly impacted upon by any of the three iterations. In both the 16-64 and 65+ age groups there was a large increase in hospital admissions for TBI at the time the 1st NICE guideline was introduced (Figure 2).

Increased imaging was intended to reduce hospital admissions by reducing diagnostic uncertainty but the 1st NICE guideline coincided with the introduction of the 4-hour target.[8] [20] We have shown, using Scottish data assessing the impact of similar (SIGN) guidelines (introduced at a different time to the 4-hour target), that the 4-hour target acted to undermine the effect of head injury guidelines and cause a large increase in hospital admissions.[21] No mortality benefit was found at the time of the introduction of the 4-hour target in England.

Later guidelines were associated with a reduction in hospital admissions rates in both adult populations assessed (Figure 2). Further increases in CT imaging may have reduced hospital admissions, as intended, by reducing diagnostic uncertainty in the ED, without the distorting effect of the 4-hour target introduction.

Strengths:

We used complete national data for England to assess the impact of the NICE head injury guidelines on mortality after admission for TBI at a population level. We have used individual level patient data to define TBI deaths and admissions. We controlled for seasonal

For peer review only

factors and auto-correlation using established techniques.[18] We used mid-year population estimates to adjust for changes in the demography of England's population.

Weaknesses

Ideally, we would have estimated the impact of the guidelines on case fatality, as this better measures the effect on the population at risk. The impact on case fatality of those attending ED with TBI could not be estimated because ED data were not collected until 2007. The impact on case fatality of those admitted with TBI could be estimated but because the guidelines resulted in changes in admissions policies and rates, the rate of deaths per admission is difficult to interpret. Instead we analysed the impact on the population TBI mortality rate, as this represents the best available unbiased measure of the guidelines' impact. We were unable to assess possible impact on disability or other patient reported outcomes, as they are not routinely collected.

ONS linked HES data is based on routinely collected administrative data; these can suffer from poor accuracy of injury coding.[22] This is particularly likely in older patients with multimorbidity (TARN – personal communication). Random poor coding, as opposed to a discrete and systematic change in coding practice, however, is unlikely to account for discontinuities observed at the specific time points of interest but may make a discontinuity harder to detect. ONS changed from ICD9 to ICD10 coding of cause of death in 2001. A sensitivity analysis excluding the period that used ICD9 coding did not materially alter the estimate of effect of the 2nd guideline on mortality in the 16-64 age group. We are unaware of other significant changes to coding practice in the HES or ONS data during the study period. The limitations of HES data mean that mortality rates could not be adjusted for anatomical severity of brain injury and presenting physiology. However, adjustment for other known predictors of TBI mortality did not materially change estimates of guideline effect and we are unaware of evidence that the prevalence of these factors changed at the point individual guidelines were introduced.

The impact of guidelines is limited by how well they are implemented. The NICE head injury guidelines have been found to be well implemented,[28] albeit with less compliance to CT imaging recommendations in the paediatric population.[23 24] There is evidence that the

For peer review only - http://bmjopen.bmj.com/site/about/guidelines.xhtml

guidelines caused factorial increases in CT head scanning in other age groups, particularly in those 65+.[10 25]

The reconfiguration of the trauma network in England in 2012 is a co-intervention which could affect the TBI mortality rate.[26] However, we found no impact on mortality associated with the 2014 NICE guideline introduced around this time.

Comparison to previous literature:

Few previous studies assess the impact of the NICE head injury guidelines.[9] A cohort study using Trauma Audit and Research Network (TARN) national registry data suggested the increased rate of transfer of severe TBI patients to neuroscience centres between 2003-2009 was associated with a halving of severe TBI case fatality.[4] TARN data were only collected at approximately half of hospitals in England until 2012 and on a TBI patient subset. Our study, using complete national data and interrupted time series analysis, found that guideline recommended management of patients with severe injuries in specialist centres only reduced the mortality rate in the 16-64 age group.

A paediatric study analysing English HES data from 2000-2011 found a reduction in annual mortality during admissions for head injury after the introduction of 2007 NICE guideline.[24] We found a fall in the mortality rate over the study period in the 0-16 age group which was unaffected by any guideline. This may reflect the greater number of data points we used to estimate the time dependent model and use of interrupted time series analysis to assess for discontinuities. We also used ONS linked HES data to identify deaths directly attributable to TBI up to 30 days following discharge.

An economic evaluation of the NICE guidelines found them to be cost effective due to a reduction in hospital admissions predicted from early single centre studies and improved outcomes.[8 10] A subsequent study using HES data found hospital admissions for head injury increased after the introduction of the 1st NICE guideline.[11] The similar increase in adult TBI admissions we found associated with the 1st NICE guideline probably is due the 4-hour target.[21] We found subsequent NICE guidelines improved outcomes and reduced hospital admissions in the 16-64 but not the 65+ age group, implying the guidelines were less cost-effective in older patients.

For peer review only - http://bmjopen.bmj.com/site/about/guidelines.xhtml

Other studies using TARN data have found increases in TBI in patients 65+ disproportionate to population changes and it has been suggested that better case ascertainment due to increased CT imaging in older patients may account for this.[2,25] The large increase in admissions for TBI for those 65+ we found at the point the first guideline was introduced, although boosted by the 4-hour target, supports this (Fig 2a and Table 3). The lack of improvement in admitted TBI mortality in older patients following the 2nd NICE guideline could either result from unequal access to treatment in specialist centres or such treatment appearing to be less effective in this group. The TARN older persons audit found patients aged over 60 to be less likely to be manged in Major Trauma Centres (where neurosurgical units are located in England) and more likely to experience delays in investigation and be treated by junior staff.[25] However, other studies have found age to be an independent predictor of mortality that is unaffected by early treatment in neuroscience centres.[27,28]

We are unaware of comparable national evaluations of the impact of head injury guidelines. Evaluations of international Brain Trauma Foundation guidelines, particularly in the USA, have utilised evidence from single centre studies or subsets of patients.[23,29,30] Evaluation of their national impact has not been possible due to their variable implementation.[23,30]

Implications:

We found evidence that only the second NICE head injury guideline was associated with a change in population based TBI mortality. This guideline contained a recommendation for increased management of severe TBI in specialist centres. Much research has focused on determining which head injured patients require CT imaging.[3,31] Increased diagnosis by itself, however, without a change in subsequent patient management was not associated with improved outcomes in our analysis. Even if apparent increases in TBI rates in older patients reflect the identification of previously unmet need, this still represents a significant health service challenge. Routine ICD coding of TBI is particularly problematic in this group and robust evaluation of treatment in specialist neuroscience centres and other interventions may be required to improve outcomes in older TBI patients. The UK, however, has one of the lowest numbers of ICU beds per population in Europe and when the 2007 guideline recommendation was made concerns were raised about the system meeting demand.[9,32] Research needs to focus on how to best configure and ration specialist services for TBI in a transparent and evidenced based way.

For peer review only - http://bmjopen.bmj.com/site/about/guidelines.xhtml

Conclusion

This first national evaluation suggests that the introduction of the second NICE head injury guideline was associated with a reduction in the admitted TBI mortality rate in the 16-64 age group and a reduction in TBI admissions in England. The guidelines were not associated with significant changes in the secular trend for TBI admissions and subsequent mortality in children and those aged 65+. Research is needed to identify clinically and cost-effective management approaches for TBI in older patients.

Acknowledgements:

The Hull and East Yorkshire NHS Trust Trans-Humber Consumer Research Panel and Hull branch of the Headway charity helped develop the research questions addressed in this study.

Authors' contributions:

This idea for the study was conceived by Carl Marincowitz with help from Trevor Sheldon, Fiona Lecky and Victoria Allgar. The analysis was completed by Carl Marincowitz with specialist advice regarding interrupted time series analysis from Trevor Sheldon and Victoria Allgar. Fiona Lecky provided specialist advice regarding the clinical context and interpretation of the results. All authors read and approved the final manuscript.

Data Sharing

Access to the individual level Office of National Statistics linked Hospital Episode Statistics is subject to a data sharing agreement with NHS Digital that limits access to the data to named members of the research team.

Competing interests:

The authors declare that they have no competing interests.

Funding:

Carl Marincowitz is funded by a National Institute for Health Research Doctoral Fellowship (DRF-2016-09-086). This study presents independent research funded by the National Institute for Health Research (NIHR). The views expressed are those of the author(s) and not necessarily those of the NHS, the NIHR or the Department of Health.

For peer review only

For peer review only - http://bmjopen.bmj.com/site/about/guidelines.xhtml

Figures:

Figure 1: The impact of the NICE Head Injury Guidelines on monthly TBI mortality rate per 100 000 population

Figure 2: The impact of the NICE Head Injury Guidelines on monthly TBI hospital admissions per 100, 000 population

For peer review only

For peer review only - http://bmjopen.bmj.com/site/about/guidelines.xhtml

1. Maas AIR, Menon DK, Adelson PD, et al. Traumatic brain injury: integrated approaches to improve prevention, clinical care, and research. *Lancet Neurol* 2017;16(12):987-1048. doi: 10.1016/S1474-4422(17)30371-X [published Online First: 2017/11/11]
2. Lawrence T, Helmy A, Bouamra O, et al. Traumatic brain injury in England and Wales: prospective audit of epidemiology, complications and standardised mortality. *BMJ Open* 2016;6(11):e012197. doi: 10.1136/bmjopen-2016-012197
3. NICE. National Clinical Guidance Centre. (2014). CG 176 Head Injury Triage, assessment, investigation and early management of head injury in children, young people and adults. National Institute for Health and Care Excellence. In: NICE, ed. UK: DOH, 2014.
4. Fuller G, Bouamra O, Woodford M, et al. Temporal trends in head injury outcomes from 2003 to 2009 in England and Wales. *Br J Neurosurg* 2011;25(3):414-21. doi: 10.3109/02688697.2011.570882
5. NICE. National Clinical Guidance Centre. (2007). Guideline CG56: Head injury: Triage, assessment, investigation and early management of head injury in infants, children and adults. National Institute for Health and Care Excellence. UK: DOH; 2007.
6. NICE. National Clinical Guidance Centre. (2003). Clinical guideline 4: Head injury: triage, assessment, investigation and early management of head injury in infants, children and adults. National Institute for Health and Care Excellence. UK: DOH; 2003.
7. Hodgkinson S, Pollit V, Sharpin C, et al. Early management of head injury: summary of updated NICE guidance. *BMJ* 2014;348:g104. doi: 10.1136/bmj.g104 [published Online First: 2014/01/24]
8. Pandor A, Goodacre S, Harnan S, et al. Diagnostic management strategies for adults and children with minor head injury: a systematic review and an economic evaluation. *Health Technol Assess* 2011;15(27):1-202. doi: 10.3310/hta15270
9. Barratt H, Wilson M, Moore F, et al. The implications of the NICE guidelines on neurosurgical management for all severe head injuries: systematic review. *Emerg Med J* 2010;27(3):173-8. doi: 10.1136/emj.2009.075382 [published Online First: 2010/03/23]
10. Hassan Z, Smith M, Littlewood S, et al. Head injuries: a study evaluating the impact of the NICE head injury guidelines. *Emerg Med J* 2005;22(12):845-9. doi: 10.1136/emj.2004.021717
11. Goodacre S. Hospital admissions with head injury following publication of NICE guidance. *Emerg Med J* 2008;25(9):556-7. doi: 10.1136/emj.2007.055723
12. Coronado VG, Xu L, Basavaraju SV, et al. Surveillance for traumatic brain injury-related deaths--United States, 1997-2007. *MMWR Surveill Summ* 2011;60(5):1-32. [published Online First: 2011/05/06]
13. Castelli A, Laudicella M, Street A. Measuring NHS Output growth. *CHE research paper* 2008; **043**: University of York. http://www.york.ac.uk/inst/che/pdf/
rp43.pdf.
14. Campbell MJ, Jacques RM, Fotheringham J, et al. Developing a summary hospital mortality index: retrospective analysis in English hospitals over five years. *BMJ* 2012;344:e1001. doi: 10.1136/bmj.e1001 [published Online First: 2012/03/03]
15. Bouamra O, Jacques R, Edwards A, et al. Prediction modelling for trauma using comorbidity and 'true' 30-day outcome. *Emerg Med J* 2015;32(12):933-8. doi: 10.1136/emermed-2015-205176
16. Office of National Statistics https://www.nomisweb.co.uk/articles/924.aspx.
17. Linden A. Conducting interrupted time-series analysis for single- and multiple-group comparisons. *The Stata Journal* 2015;15(2):480-500.
18. Lopez Bernal J, Cummins S, Gasparrini A. Interrupted time series regression for the evaluation of public health interventions: a tutorial. *Int J Epidemiol* 2016 doi: 10.1093/ije/dyw098 [published Online First: 2016/06/11]

For peer review only - http://bmjopen.bmj.com/site/about/guidelines.xhtml

19. Hamilton I, Lloyd C, Hewitt C, et al. Effect of reclassification of cannabis on hospital admissions for cannabis psychosis: a time series analysis. *Int J Drug Policy* 2014;25(1):151-6. doi: 10.1016/j.drugpo.2013.05.016 [published Online First: 2013/07/23]
20. Comptroller General. Improving Emergency Care in England. *National Audit Office* 2004; HC 1075 Session 2003-2004. https://www.nao.org.uk/wp-content/uploads/2004/10/03041075.pdf
21. Marincowitz C. The impact of the SIGN head injury guidelines and NHS 4-hour Emergency Target on hospital admissions for head injury in Scotland: An Interrupted Times Series. *BMJ Open* 2018 In Press
22. Hand DJ. Statistical challenges of administrative and transaction data. *J R Statist Soc A* 2018 (pre-publication presented at The Royal Statistical Society);181(Part 3):1-24.
23. Cnossen MC, Scholten AC, Lingsma HF, et al. Adherence to Guidelines in Adult Patients with Traumatic Brain Injury: A Living Systematic Review. *J Neurotrauma* 2016 doi: 10.1089/neu.2015.4121 [published Online First: 2015/10/04]
24. Marlow R, Mytton J, Maconochie IK, et al. Trends in admission and death rates due to paediatric head injury in England, 2000-2011. *Arch Dis Child* 2015;100(12):1136-40. doi: 10.1136/archdischild-2015-308615
25. *The Trauma and Audit Research Network Report* 2017. Major Trauma in Older People. https://www.tarn.ac.uk/content/downloads/3793/Major%20Trauma%20in%20Older%20People%202017.pdf
26. Yiannoullou P, Hall C, Newton K, et al. A review of the management of blunt splenic trauma in England and Wales: have regional trauma networks influenced management strategies and outcomes? *Ann R Coll Surg Engl* 2017;99(1):63-69. doi: 10.1308/rcsann.2016.0325
27. Fountain DM, Kolias AG, Lecky FE, et al. Survival Trends After Surgery for Acute Subdural Hematoma in Adults Over a 20-year Period. *Ann Surg* 2017;265(3):590-96. doi: 10.1097/SLA.0000000000001682
28. Utomo WK, Gabbe BJ, Simpson PM, et al. Predictors of in-hospital mortality and 6-month functional outcomes in older adults after moderate to severe traumatic brain injury. *Injury* 2009;40(9):973-7. doi: 10.1016/j.injury.2009.05.034 [published Online First: 2009/06/23]
29. Faul M, Wald MM, Rutland-Brown W, et al. Using a cost-benefit analysis to estimate outcomes of a clinical treatment guideline: testing theBrain Trauma Foundation guidelines for the treatment of severe traumatic brain injury. *J Trauma* 2007;63(6):1271-8. doi: 10.1097/TA.0b013e3181493080 [published Online First: 2008/01/24]
30. Lee JC, Rittenhouse K, Bupp K, et al. An analysis of Brain Trauma Foundation traumatic brain injury guideline compliance and patient outcome. *Injury* 2015;46(5):854-8. doi: 10.1016/j.injury.2014.12.023 [published Online First: 2015/02/11]
31. Foks KA, van den Brand CL, Lingsma HF, et al. External validation of computed tomography decision rules for minor head injury: prospective, multicentre cohort study in the Netherlands. *BMJ* 2018;362:k3527. doi: 10.1136/bmj.k3527 [published Online First: 2018/08/26]
32. Murthy S, Wunsch H. Clinical review: International comparisons in critical care - lessons learned. *Crit Care* 2012;16(2):218. doi: 10.1186/cc11140 [published Online First: 2012/05/02]

For peer review only - http://bmjopen.bmj.com/site/about/guidelines.xhtml

Figure 1: The impact of the NICE Head Injury Guidelines on monthly TBI mortality rate per 100 000 population

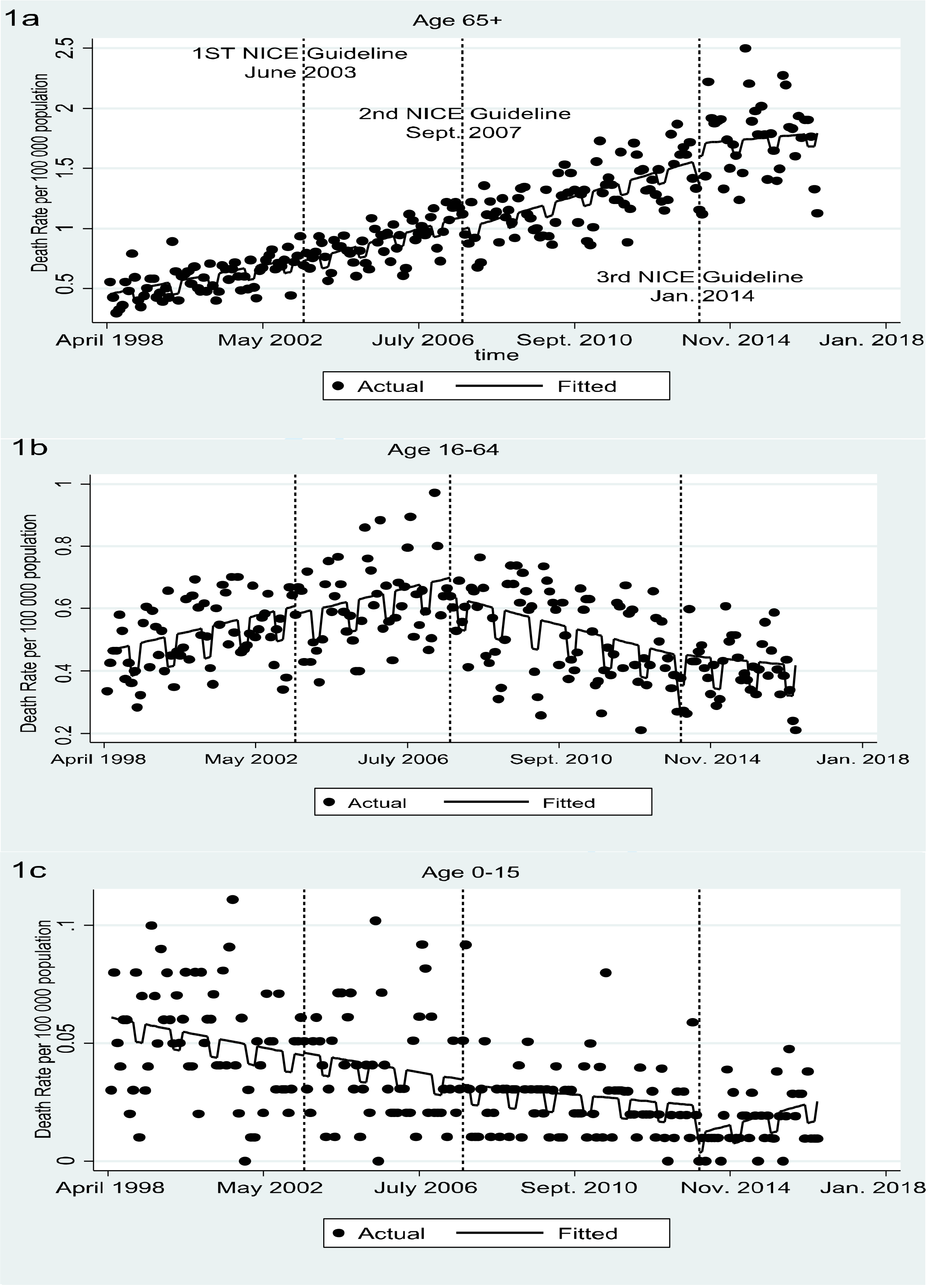

Figure 2: The impact of the NICE Head Injury Guidelines on monthly TBI hospital admissions per 100, 000 population

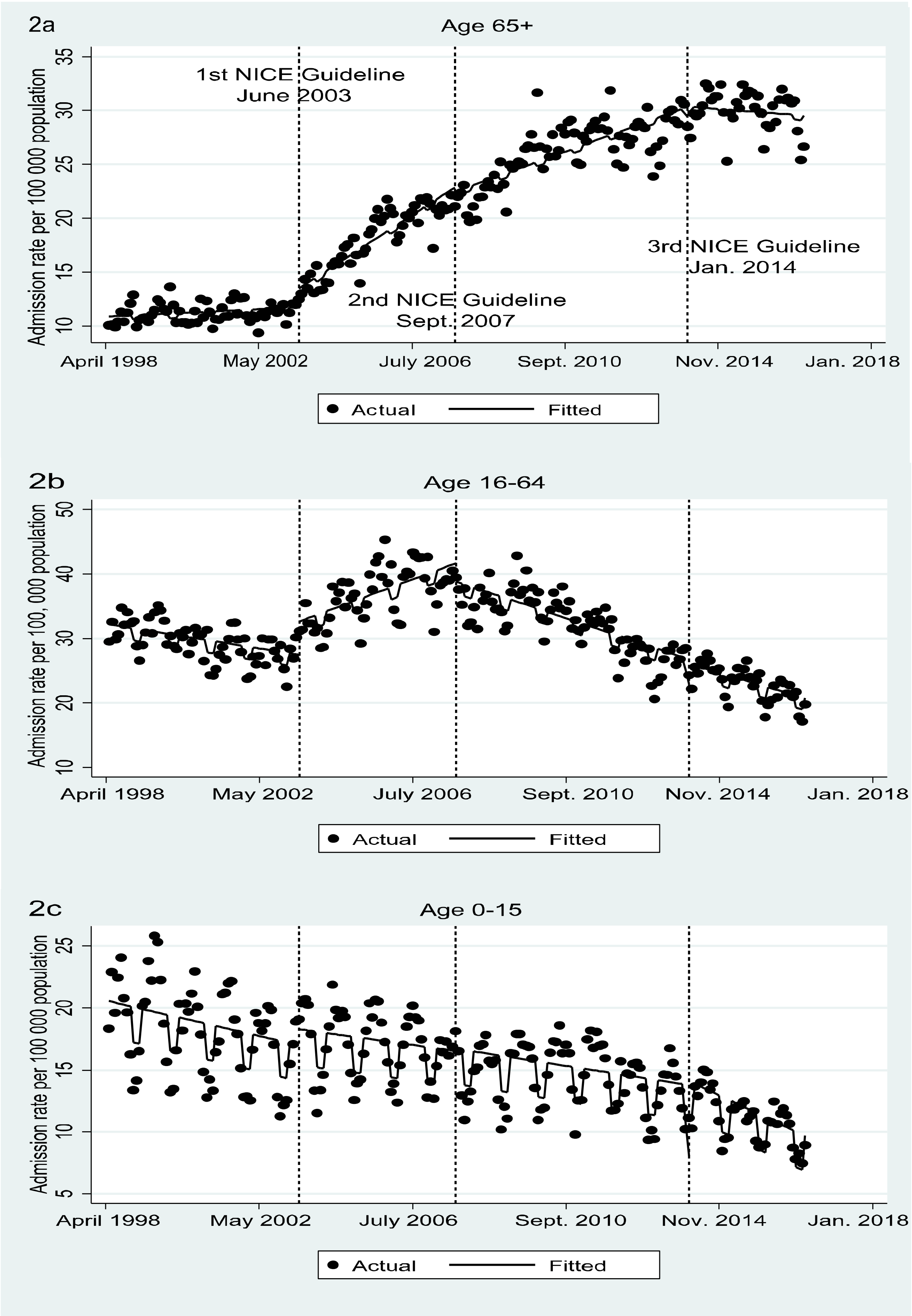

Supplementary Material 1: Key Features of the NICE Head Injury Guidelines

Policy	Time of Introduction	Key Features
1st NICE Head Injury Guideline	June 2003	Indication for CT imaging (referenced directly from 2003 Guideline):<br><br>- GCS less than 13 on initial assessment in the emergency department.<br>- GCS equal to 13 or 14 at 2 hours after the injury on assessment in the emergency department.<br>- Suspected open or depressed skull fracture.<br>- Any sign of basal skull fracture (haemotympanum, 'panda' eyes, cerebrospinal fluid otorrhoea, Battle's sign).<br>- Post-traumatic seizure.<br>- Focal neurological deficit.<br>- More than one episode of vomiting.<br>- Amnesia for greater than 30 minutes of events before impact.<br><br>CT should also recommended in patients with any of the following risk factors, provided they have experienced some loss of consciousness or amnesia since the injury:<br>-Age greater than or equal to 65 years.<br>-Coagulopathy (history of bleeding, clotting disorder, current treatment with warfarin).<br>-Dangerous mechanism of injury (a pedestrian struck by a motor vehicle, an occupant ejected from a motor vehicle or a fall from a height of greater than 1 metre or five stairs).
2nd NICE Head Injury Guideline	September 2007	Specialist management (referenced directly from 2007 Guideline):<br><br>Local guidelines on the transfer of patients with head injuries should be drawn up between the referring hospital trusts, the neuroscience unit and the local ambulance service, and should recognise that:<br>-Transfer would benefit all patients with serious head injuries (GCS<9), irrespective of the need for neurosurgery

For peer review

		-If transfer of those who do not require neurosurgery is not possible, ongoing liaison with the neuroscience unit over clinical management is essential.<br><br>Indications Immediate CT scanning (adult):<br>- Glasgow coma score <13 on initial assessment in the emergency department<br>- Glasgow coma score <15 two hours after the injury on assessment in the emergency department<br>- Suspected open or depressed skull fracture<br>- Any sign of basal skull fracture<br>- Post-traumatic seizure<br>- Focal neurological deficit<br>- One or more episodes of vomiting<br>- Amnesia for events more than 30 minutes before impact.<br><br>Indications Immediate CT scanning (<16 years):<br>- Age over 1 year: Glasgow coma score <14 on assessment in the emergency department<br>- Age under 1 year: Glasgow coma score paediatric <15 on assessment in the emergency department<br>- Age under 1 year and presence of bruise, swelling, or laceration (>5 cm) on the head<br>- Clinical suspicion of non-accidental injury<br>- Post-traumatic seizure but no history of epilepsy<br>- Abnormal drowsiness<br>- Suspected open or depressed skull injury, or tense fontanelle<br>- Any sign of basal skull fracture<br>- Focal neurological deficit<br>- Three or more discrete episodes of vomiting<br>- Amnesia (antegrade or retrograde) lasting more than five minutes.
3rd NICE Head Injury Guideline	January 2014	Referenced directly from 3nd NICE Guidelines<br><br>Indications CT scanning < 1 hour (adult):<br>- GCS<13/15<br>- GCS <15 after2 hours from injury<br>- Suspected open or depressed skull fracture<br>- Any sign of basal skull fracture

For peer review only

		- Post-traumatic seizure<br>- Focal neurological deficit<br>- One or more episodes of vomiting<br>Indications CT scanning < 8 hours (adult):<br>- Patient taking warfarin<br>- LOC or amnesia + dangerous mechanism/age 65+/history of bleeding/clotting disorder<br>- Amnesia for events more than 30 minutes before impact.<br><br><br>Indications CT scanning < 1 hour (<16 years) if 1 of:<br>- Suspicion of non-accidental injury<br>- Post-traumatic seizure but no history of epilepsy.<br>- On initial emergency department assessment, GCS less than 14, or for children under 1 year GCS (paediatric) less than 15.<br>- At 2 hours after the injury, GCS less than 15.<br>- Suspected open or depressed skull fracture or tense fontanelle.<br>- Any sign of basal skull fracture (haemotympanum, 'panda' eyes, cerebrospinal fluid leakage from the ear or nose, Battle's sign).<br>- Focal neurological deficit.<br>- For children under 1 year, presence of bruise, swelling or laceration of more than 5 cm on the head.<br>Indications CT scanning < 1 hour (<16 years) if 2 or more of:<br>- Loss of consciousness lasting more than 5 minutes (witnessed).<br>- Abnormal drowsiness.<br>- Three or more discrete episodes of vomiting.<br>- Dangerous mechanism of injury<br>- Amnesia (antegrade or retrograde) lasting more than 5 minutes[4].<br>If only 1 above risk factor observe for 4 hours post injury if during observation develop any risk factor below for CT within 1 hour<br>- GCS less than 15.<br>- Further vomiting.<br>- A further episode of abnormal drowsiness.<br>If taking warfarin for CT within 8 hours.

Supplementary Material 2: Flow diagram of identification of deaths attributable to TBI used in analysis

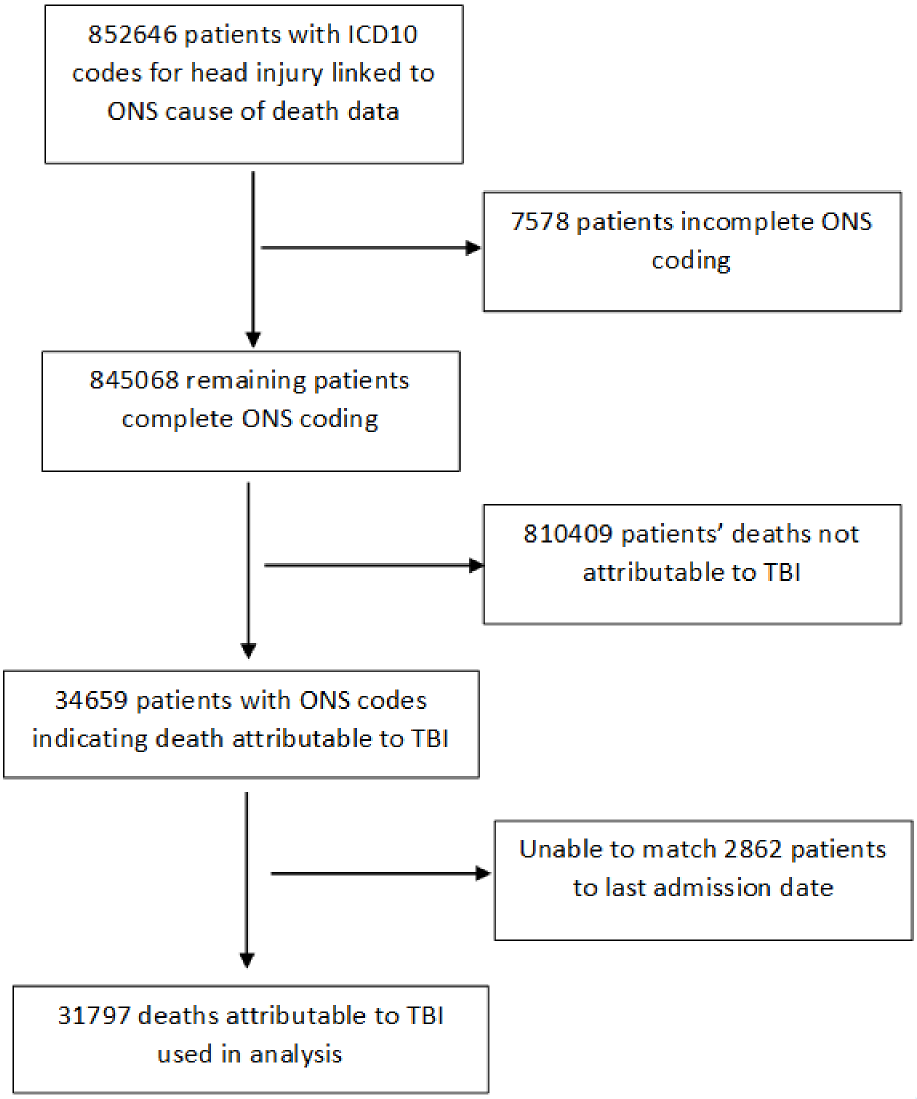

Supplementary Material 3: Monthly admission characteristics of patients with TBI:

Median age (65 and over)

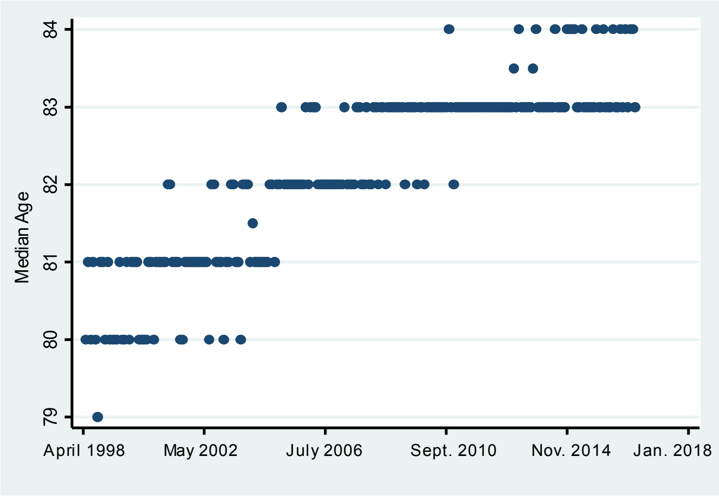

Proportion Male (65 and over)

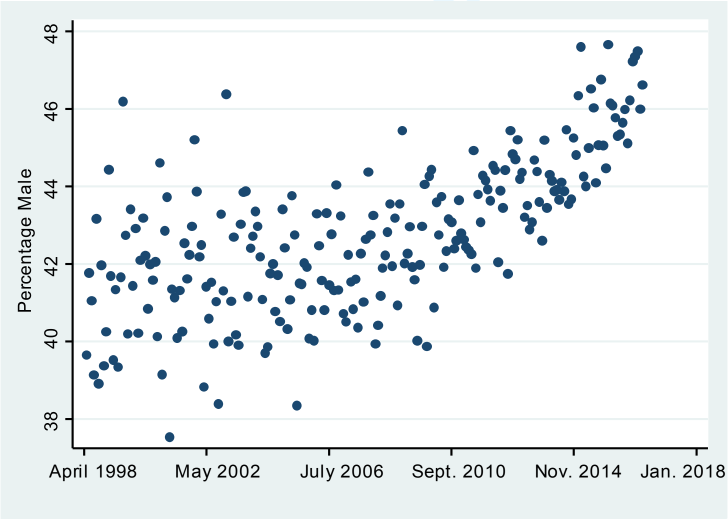

Mean Monthly Charlson Score (65 and over)

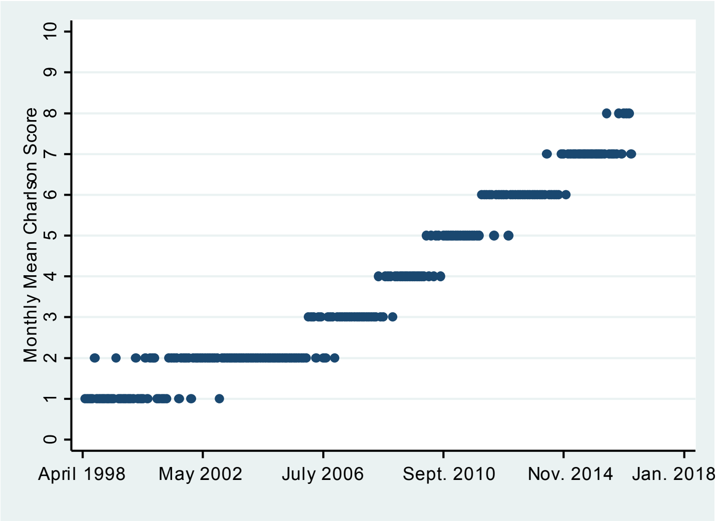

Mean Monthly Trauma Modified Charlson Score (65 and over)

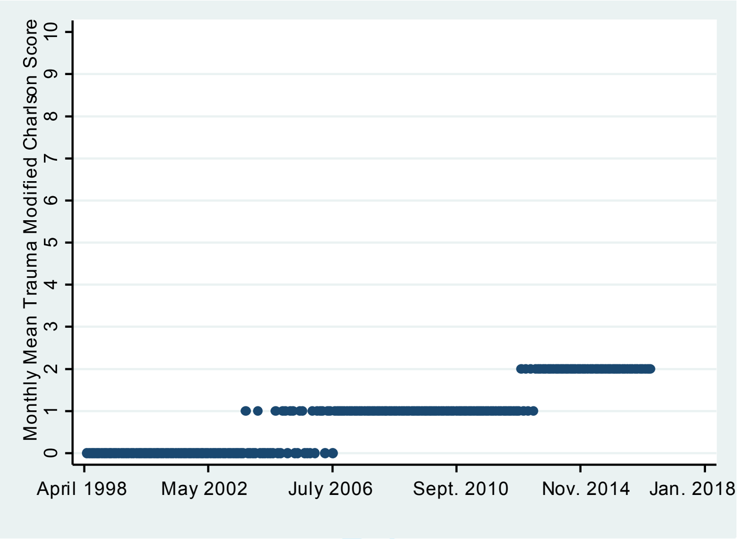

Median age (16-64)

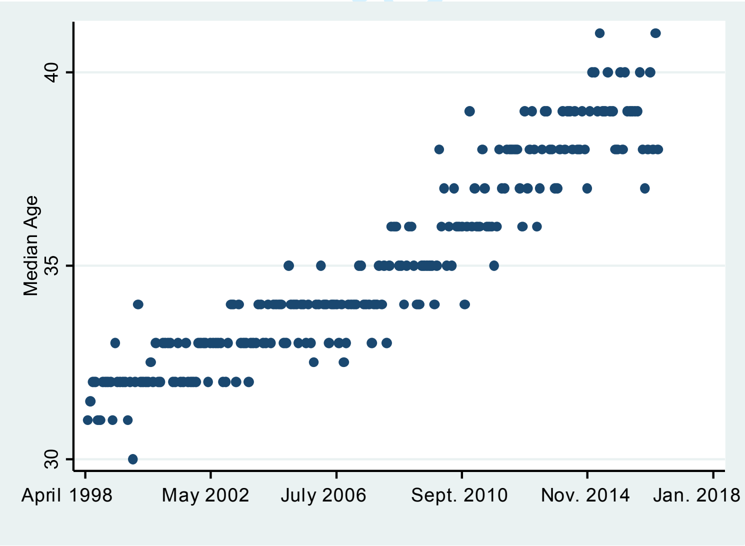

Proportion Male (16-64)

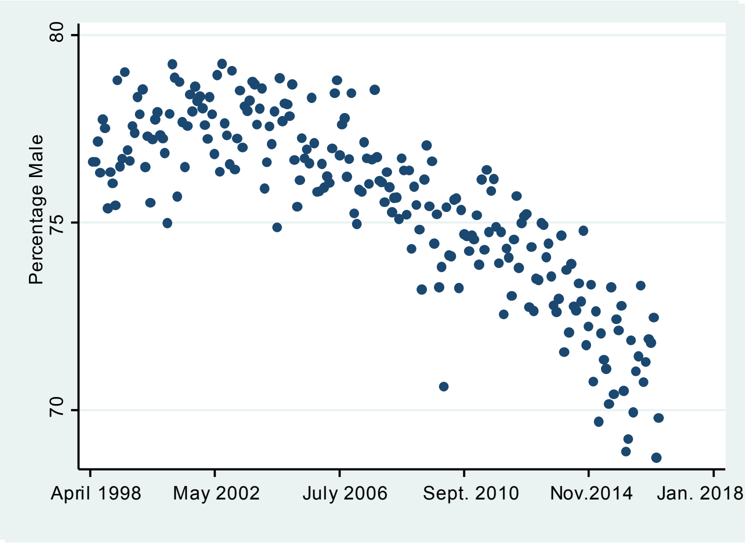

Mean Monthly Standard Charlson Score (16-64)

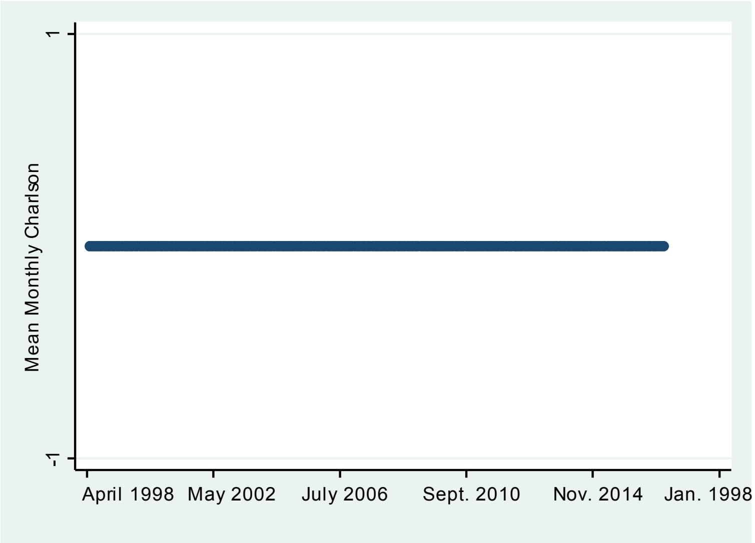

Mean Monthly Trauma Modified Charlson Score (16-64)

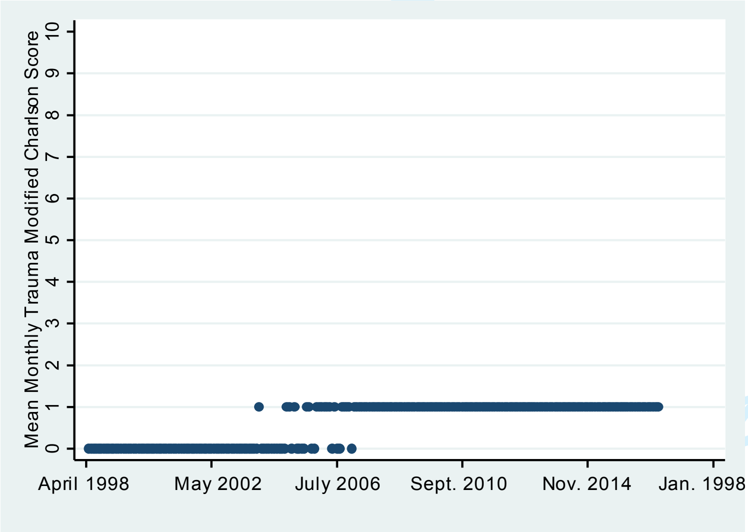

Median age (0-15)

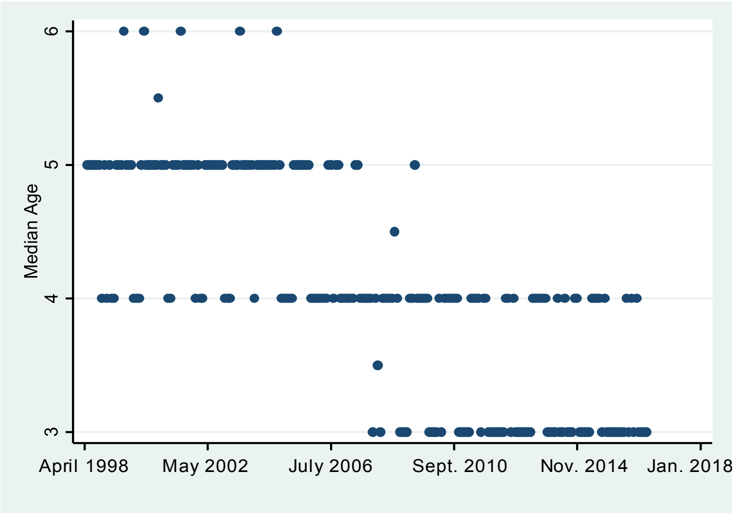

Proportion Male (0-15)

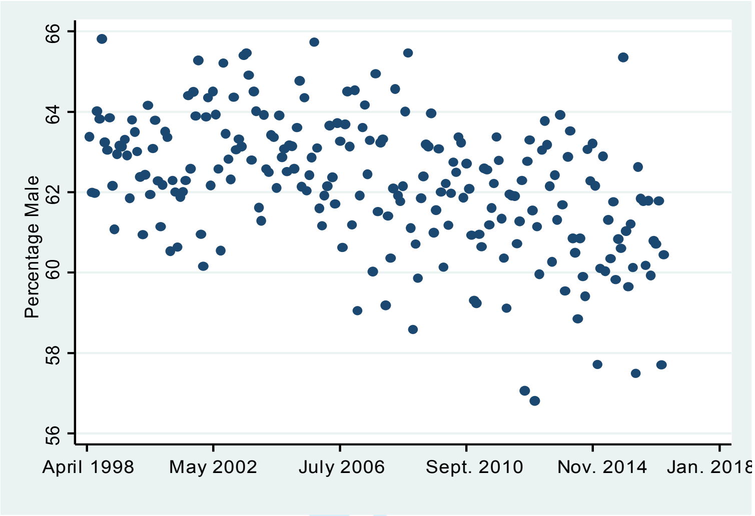

Mean Monthly Standard Charlson Score (0-15)

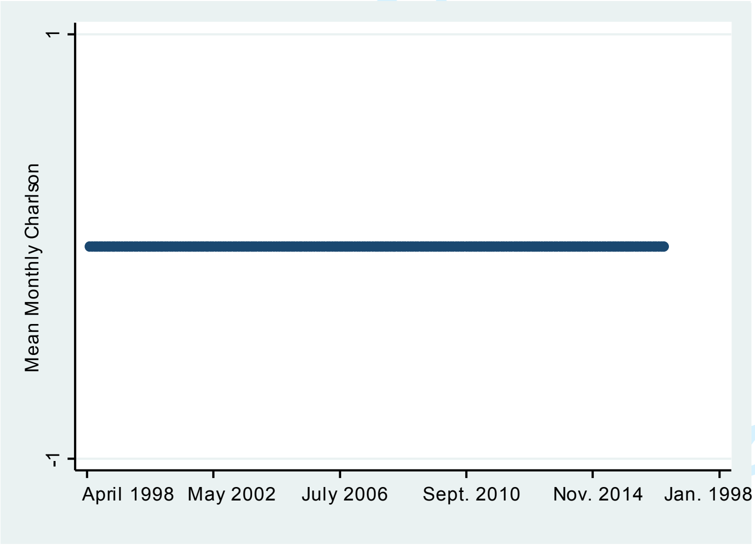

Mean Monthly Trauma Modified Charlson Score (0-15)

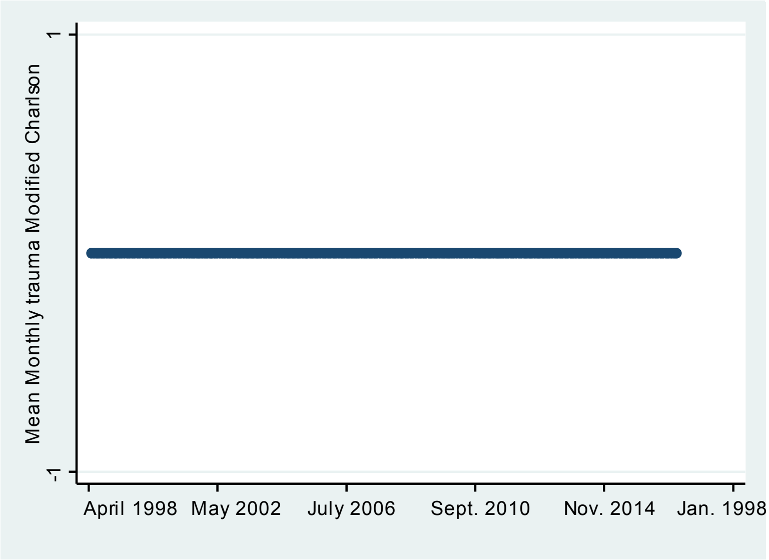

For peer review only - http://bmjopen.bmj.com/site/about/guidelines.xhtml

Supplementary Material 4: Subgroup analysis of effect of the NICE guidelines on patients aged 65 deaths per 100, 000 population

**Age Band**	**Winter Effect**	**Initial Trend**	**1st NICE Guideline**	**2nd NICE Guideline**	**3rd NICE Guideline**	**Durbin-Watson Statistic**
**65-84**	-0.06<br>(95% CI: -0.1 to -0.02)<br>P=0.01	0.003<br>(95% CI: 0.001 to 0.005)<br>P=0.006	**Change level:**<br>-0.02<br>(95% CI:-0.13 to 0.1)<br>P=0.78	**Change level:**<br>-0.07<br>(95% CI: -0.19 to 0.04)<br>P=0.21	**Change level:**<br>0.09<br>(95% CI:-0.03 to 0.21)<br>P=0.15	Untransformed 1.62<br>Prais-Winsten 1.89
			**Change trend:**<br>0.001<br>(95% CI:-0.002 to 0.005)<br>P=0.51	**Change trend:**<br>-0.001<br>(95% CI: -0.005 to 0.002)<br>P=0.44	**Change trend:**<br>-0.003<br>(95% CI:-0.008 to 0.001)<br>P=0.16	
**85+**	-0.46<br>(95% CI: -0.73 to -0.2)<br>P<0.01	0.02<br>(95% CI: 0.01 to 0.03)<br>P=0.01	**Change level:**<br>-0.03<br>(95% CI:-0.7 to 0.7)<br>P=0.92	**Change level:**<br>-0.38<br>(95% CI: -1.05 to 0.29)<br>P=0.27	**Change level:**<br>0.54<br>(95% CI:-0.18 to 1.26)<br>P=0.14	Untransformed 1.68<br>Prais-Winsten 1.91
			**Change trend:**<br>0.001<br>(95% CI:-0.02 to 0.02)<br>P=0.9	**Change trend:**<br>0.02<br>(95% CI: -0.001 to 0.04)<br>P=0.65	**Change trend:**<br>-0.02<br>(95% CI:-0.05 to 0.01)<br>P=0.15	

For peer review only

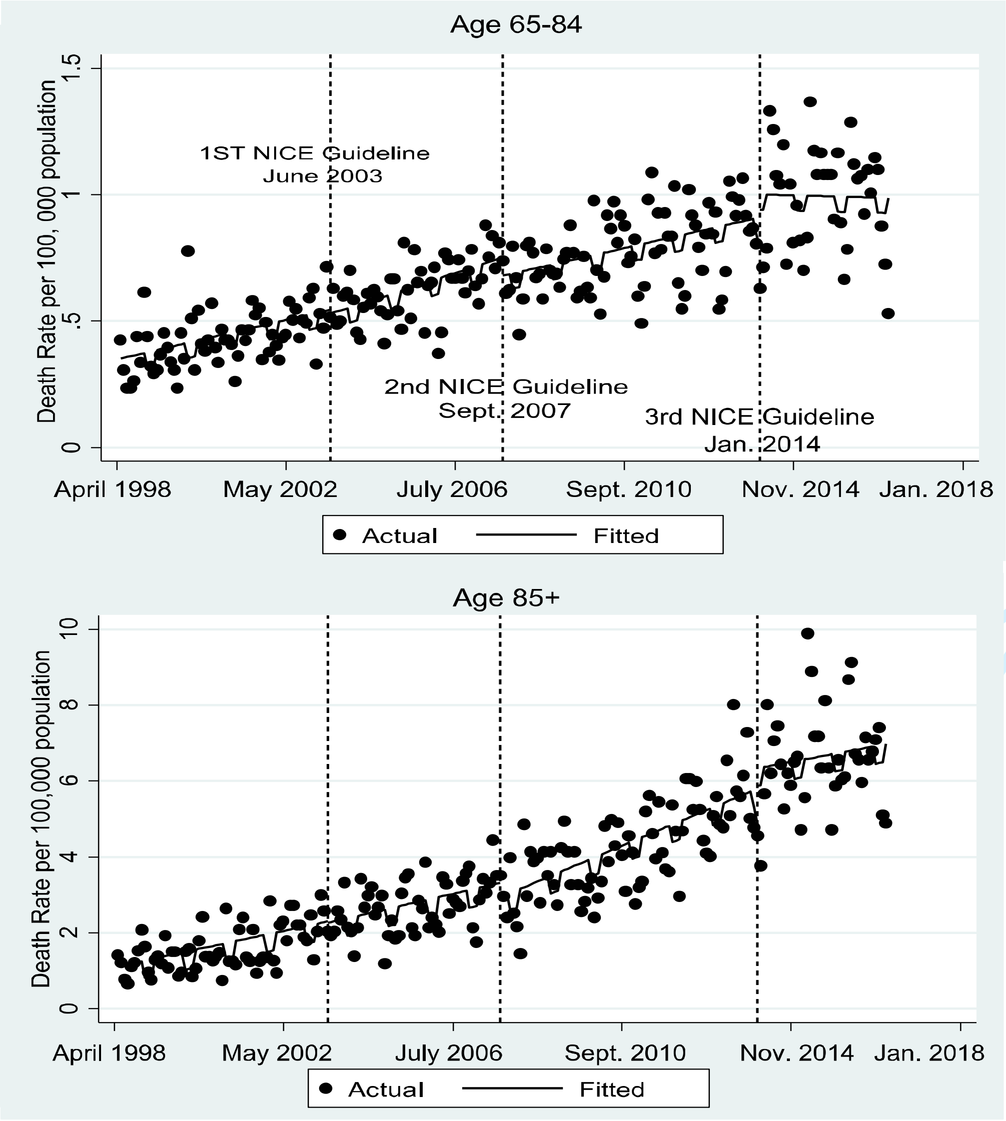
Age 65-84
Death Rate per 100, 000 population
1.5
1
.5
0
1ST NICE Guideline
June 2003
2nd NICE Guideline
Sept. 2007
3rd NICE Guideline
Jan. 2014
April 1998
May 2002
July 2006
Sept. 2010
Nov. 2014
Jan. 2018
Actual
Fitted
Age 85+
Death Rate per 100,000 population
10
8
6
4
2
0
April 1998
May 2002
July 2006
Sept. 2010
Nov. 2014
Jan. 2018
Actual
Fitted

Supplementary Material 5: The impact of the NICE head injury guidelines on monthly TBI mortality rate per 100 000 population adjusted for age, sex and comorbidity

**Age Band**	**Winter Effect**	**Initial Trend**	**Median Age**	**Proportion Male**	**Charlson Score**	**1st NICE Guideline**	**2nd NICE Guideline**	**3rd NICE Guideline**	**Durbin-Watson Statistic**
**65+**	-0.1<br>(95% CI: -0.17 to -0.04)<br>P<0.01	0.006<br>(95% CI: 0.002 to 0.009)<br>P<0.01	-0.03<br>(95% CI: -0.09 to 0.02)<br>P=0.25	0.03<br>(95% CI: -1.80 to 1.87)<br>P=0.97	0.00003<br>(95% CI: -0.07 to 0.07)<br>P>0.99	<u>**Change level:**</u><br>-0.04<br>(95% CI:-0.22 to 0.14)<br>P=0.69	<u>**Change level:**</u><br>-0.1<br>(95% CI: -0.28 to 0.07)<br>P=0.25	<u>**Change level:**</u><br>0.14<br>(95% CI:-0.05 to 0.32)<br>P=0.15	Untransformed 1.56<br>Prais-Winsten 1.86
						<u>**Change trend:**</u><br>0.003<br>(95% CI: -0.003 to 0.008)<br>P=0.39	<u>**Change trend:**</u><br>-0.0002<br>(95% CI:-0.006 to 0.005)<br>P=0.95	<u>**Change trend:**</u><br>-0.005<br>(95% CI:-0.01 to 0.002)<br>P=0.14	
**16-64**	-0.12<br>(95% CI: -0.15 to -0.09)<br>P<0.01	0.001<br>(95% CI: -0.0003 to 0.003)<br>P=0.1	0.03<br>(95% CI: 0.01 to 0.05)<br>P<0.01	1.40<br>(95% CI:0.1 to 2.69)<br>P=0.04	Not adjusted for as no change over time period.	<u>**Change level:**</u><br>-0.03<br>(95% CI:-0.11 to 0.06)<br>P=0.52	<u>**Change level:**</u><br>0.06<br>(95% CI:-0.14 to 0.02)<br>P=0.15	<u>**Change level:**</u><br>-0.0004<br>(95% CI: -0.085 to 0.085)<br>P=0.99	Untransformed 1.89<br>Prais-Winsten 1.98
						<u>**Change trend:**</u><br>0.0001<br>(95% CI: -0.002 to 0.004)<br>P=0.52	<u>**Change trend:**</u><br>*-0.006*<br>*(95% CI: -0.008 to -0.003)*<br>*P<0.01*	<u>**Change trend:**</u><br>0.003<br>(95% CI: 0.00005 to 0.007)<br>P=0.047	
**0-15**	-0.01<br>(95% CI: -0.01 to 0.001)<br>P=0.09	-0.0002<br>(95% CI: -0.0005 to -0.00002)<br>P=0.04	0.006<br>(95% CI: 0.00002 to 0.01) P=0.049	-0.09<br>(95% CI:-0.28 to 0.09)<br>P=0.32	Not adjusted for as no change over time period.	<u>**Change level:**</u><br>0.0001<br>(95% CI: -0.01 to 0.01) P= 0.99	<u>**Change level:**</u><br>-0.0004<br>(95% CI: -0.01 to 0.01) P=0.95	<u>**Change level:**</u><br>-0.01<br>(95% CI:-0.03 to 0.001) P=0.08	Untransformed 2.19<br>Prais-Winsten 1.99
						<u>**Change trend:**</u><br>0.0001<br>(95% CI:-0.0003 to 0.0005) P=0.58	<u>**Change trend**</u><br>0.00005<br>(95% CI:-0.0003 to 0.0004)<br>P=0.81	<u>**Change trend:**</u><br>0.0004<br>(95% CI: -0.00007 to 0.001) P=0.09	

For peer review only

Supplementary Material 6: Sensitivity analysis of implementation lags on the impact of the NICE head injury guidelines on deaths per 100 000 population

Age Band	Winter Effect	Initial Trend	1st NICE Guideline	2nd NICE Guideline	3rd NICE Guideline	Durbin-Watson Statistic
**65+**	-0.11<br>(95% CI: -0.18 to-0.04)<br>P<0.01	0.005<br>(95% CI: 0.001 to 0.008)<br>P<0.01	**Change level:**<br>-0.007<br>(95% CI:-0.2 to 0.19)<br>P=0.95	**Change level:**<br>-0.05<br>(95% CI: -0.25 to 0.14)<br>P=0.60	**Change level:**<br>0.13<br>(95% CI:-0.06 to 0.33)<br>P=0.18	Untransformed 1.56<br>Prais-Winsten 1.86
			**Change trend:**<br>0.005<br>(95% CI:-0.003 to 0.012)<br>P=0.24	**Change trend:**<br>-0.0018<br>(95% CI: -0.01 to 0.006)<br>P=0.65	**Change trend:**<br>-0.006<br>(95% CI:-0.01 to 0.002)<br>P=0.16	
**16-64**	-0.1<br>(95% CI: -0.14 to -0.06)<br>P<0.01	0.002<br>(95% CI:0.001 to 0.004)<br>P<0.01	**Change level:**<br>0.01<br>(95% CI: -0.08 to 0.11)<br>P=0.78	**Change level:**<br>0.06<br>(95% CI:-0.15 to 0.003)<br>P=0.11	**Change level:**<br>0.006<br>(95% CI: -0.09 to 0.1)<br>P=0.91	Untransformed 1.75<br>Prais-Winsten 1.94
			**Change trend:**<br>-0.001<br>(95% CI: -0.004 to 0.003)<br>P=0.77	**Change trend:**<br>-0.004<br>(95% CI:-0.008 to -0.001)<br>P=0.03	**Change trend:**<br>0.002<br>(95% CI:-0.002 to 0.005)<br>P=0.41	
**0-15**	-0.01<br>(95%CI:-0.01 to -0.001)<br>P=0.02	-0.0003<br>(95% CI: -0.0005 to -0.00001)<br>P=0.03	**Change level:**<br>0.001<br>(95% CI: -0.01 to 0.01)<br>P= 0.88	**Change level:**<br>-0.001<br>(95% CI: -0.01 to 0.01)<br>P=0.93	**Change level:**<br>-0.01<br>(95% CI:-0.03 to 0.002)<br>P=0.097	Untransformed 2.18<br>Prais-Winsten 1.98
			**Change trend:**<br>0.00007<br>(95% CI: -0.0006 to 0.0005)<br>P=0.80	**Change trend**<br>0.0002<br>(95% CI: -0.0003 to 0.0007)<br>P=0.47	**Change trend:**<br>0.0005<br>(95% CI: -0.00003 to 0.001)<br>P=0.07	

For peer review only

Supplementary Material 7: Sensitivity analysis of implementation lags on the impact of the NICE head injury guidelines on admissions per 100 000 population

**Age Band**	**Winter Effect**	**Initial Trend**	**1st NICE Guideline**	**2nd NICE Guideline**	**3rd NICE Guideline**	**Durbin-Watson Statistic**
**65+**	-0.51<br>(95% CI: -1.05 to 0.04)<br>P=0.07	0.02<br>(95% CI -0.02 to 0.05)<br>P=0.31	**Change level:**<br>3.88<br>(95% CI: 2.11 to 5.66)<br>P<0.01	**Change level:**<br>1.71<br>(95% CI: -0.08 to 3.5)<br>P=0.06	**Change level:**<br>0.6<br>(95% CI:-1.17 to 2.36)<br>P=0.51	Untransformed 1.24<br>Prais-Winsten 2.05
			**Change trend:**<br>0.17<br>(95% CI: 0.09 to 0.24)<br>P<0.01	**Change trend:**<br>-0.1<br>(95% CI: -0.17 to -0.03)<br>P=0.01	**Change trend:**<br>-0.1<br>(95% CI:-0.18 to -0.03)<br>P=0.01	
**16-64**	-2.16<br>(95% CI: -3.03 to -1.28)<br>P<0.01	-0.08<br>(95% CI:-0.12 to -0.03)<br>P<0.01	**Change level:**<br>8.6<br>(95% CI: 6 to 11.2)<br>P<0.01	**Change level:**<br>-2.22<br>(95% CI:-4.84 to 0.4)<br>P=0.1	**Change level:**<br>0.25<br>(95% CI:-2.33 to 2.84)<br>P=0.85	Untransformed 1.49<br>Prais-Winsten 2.06
			**Change trend:**<br>0.2<br>(95% CI: 0.09 to 0.3)<br>P<0.01	**Change trend:**<br>-0.32<br>(95% CI: -0.42 to -0.21)<br>P<0.01	**Change trend:**<br>0.06<br>(95% CI:-0.05 to 0.16)<br>P=0.29	
**0-15**	-2.93<br>(95% CI: -3.49 to -2.38)<br>P<0.01	-0.06<br>(95%CI:-0.11 to -0.01)<br>P=0.02	**Change level:**<br>1.16<br>(95% CI: -1.22 to 3.54)<br>P= 0.34	**Change level:**<br>0.4<br>(95% CI: -1.99 to 2.8)<br>P=0.74	**Change level:**<br>0.5<br>(95% CI:-1.87 to 2.88)<br>P=0.68	Untransformed 1.06<br>Prais-Winsten 1.71
			**Change trend:**<br>0.02<br>(95% CI: -0.1 to 0.13)<br>P=0.8	**Change trend**<br>-0.01<br>(95% CI: -0.12 to 0.1)<br>P=0.9	**Change trend:**<br>-0.06<br>(95% CI: -0.17 to 0.05)<br>P=0.28	

For peer review only

STROBE Statement—checklist of items that should be included in reports of observational studies

	Item No	Recommendation
**Title and abstract**	1	(a) Indicate the study's design with a commonly used term in the title or the abstract **Page 1**
		(*b*) Provide in the abstract an informative and balanced summary of what was done and what was found **Page 2**
**Introduction**		
Background/rationale	2	Explain the scientific background and rationale for the investigation being reported **Page 4**
Objectives	3	State specific objectives, including any prespecified hypotheses **Page 4**
**Methods**		
Study design	4	Present key elements of study design early in the paper **Page 5**
Setting	5	Describe the setting, locations, and relevant dates, including periods of recruitment, exposure, follow-up, and data collection **Pages 5,7,8**
Participants	6	(*a*) *Cohort study*—Give the eligibility criteria, and the sources and methods of selection of participants. Describe methods of follow-up **Pages 7,8**
		(*b*) *Cohort study*—For matched studies, give matching criteria and number of exposed and unexposed **N/A**
Variables	7	Clearly define all outcomes, exposures, predictors, potential confounders, and effect modifiers. Give diagnostic criteria, if applicable **Pages 7**
Data sources/ measurement	8*	For each variable of interest, give sources of data and details of methods of assessment (measurement). Describe comparability of assessment methods if there is more than one group **Pages 5,7,8**
Bias	9	Describe any efforts to address potential sources of bias **Pages 7,8**
Study size	10	Explain how the study size was arrived at **N/A**
Quantitative variables	11	Explain how quantitative variables were handled in the analyses. If applicable, describe which groupings were chosen and why **Pages 7,8**
Statistical methods	12	(*a*) Describe all statistical methods, including those used to control for confounding **Pages 7,8**
		(*b*) Describe any methods used to examine subgroups and interactions **Pages 7,8**
		(*c*) Explain how missing data were addressed
		(*d*) *Cohort study*—If applicable, explain how loss to follow-up was addressed *Case-control study*—If applicable, explain how matching of cases and controls was

For peer review only

		addressed<br>*Cross-sectional study*—If applicable, describe analytical methods taking account of sampling strategy
		(*e*) Describe any sensitivity analyses<br>**Page 7,8**
**Results**		
Participants	13*	(a) Report numbers of individuals at each stage of study—eg numbers potentially eligible, examined for eligibility, confirmed eligible, included in the study, completing follow-up, and analysed<br>**Page 8-12**
		(b) Give reasons for non-participation at each stage
		(c) Consider use of a flow diagram
Descriptive data	14*	(a) Give characteristics of study participants (eg demographic, clinical, social) and information on exposures and potential confounders<br>**Page 8-12**
		(b) Indicate number of participants with missing data for each variable of interest
		(c) *Cohort study*—Summarise follow-up time (eg, average and total amount)
Outcome data	15*	*Cohort study*—Report numbers of outcome events or summary measures over time
		Case-control study—Report numbers in each exposure category, or summary measures of exposure
		Cross-sectional study—Report numbers of outcome events or summary measures
Main results	16	(*a*) Give unadjusted estimates and, if applicable, confounder-adjusted estimates and their precision (eg, 95% confidence interval). Make clear which confounders were adjusted for and why they were included<br>**Pages 8-12**
		(*b*) Report category boundaries when continuous variables were categorized
		(*c*) If relevant, consider translating estimates of relative risk into absolute risk for a meaningful time period
Other analyses	17	Report other analyses done—eg analyses of subgroups and interactions, and sensitivity analyses
**Discussion**		
Key results	18	Summarise key results with reference to study objectives<br>**Page 13**
Limitations	19	Discuss limitations of the study, taking into account sources of potential bias or imprecision. Discuss both direction and magnitude of any potential bias<br>**Page 14**
Interpretation	20	Give a cautious overall interpretation of results considering objectives, limitations, multiplicity of analyses, results from similar studies, and other relevant evidence<br>**Pages 15, 16**
Generalisability	21	Discuss the generalisability (external validity) of the study results<br>**Pages 15, 16**
**Other information**		
Funding	22	Give the source of funding and the role of the funders for the present study and, if applicable, for the original study on which the present article is based<br>**Page 17**

For peer review only - http://bmjopen.bmj.com/site/about/guidelines.xhtml

*Give information separately for cases and controls in case-control studies and, if applicable, for exposed and unexposed groups in cohort and cross-sectional studies.

Note: An Explanation and Elaboration article discusses each checklist item and gives methodological background and published examples of transparent reporting. The STROBE checklist is best used in conjunction with this article (freely available on the Web sites of PLoS Medicine at http://www.plosmedicine.org/, Annals of Internal Medicine at http://www.annals.org/, and Epidemiology at http://www.epidem.com/). Information on the STROBE Initiative is available at www.strobe-statement.org.

For peer review only

BMJ Open

An evaluation of the impact of the NICE head injury guidelines on inpatient mortality from traumatic brain injury: an interrupted time series analysis

Journal:	*BMJ Open*
Manuscript ID	bmjopen-2019-028912.R1
Article Type:	Research
Date Submitted by the Author:	09-Mar-2019
Complete List of Authors:	Marincowitz, Carl; University of Hull, Hull York Medical School<br>Lecky, Fiona; University of Sheffield, School of Health and Related Research<br>Allgar, Victoria; York University, HYMS/Health Sciences<br>Sheldon, Trevor; University of York, health Sciences
**Primary Subject Heading**:	Emergency medicine
Secondary Subject Heading:	Health policy, Neurology, Health services research
Keywords:	ACCIDENT & EMERGENCY MEDICINE, Health policy < HEALTH SERVICES ADMINISTRATION & MANAGEMENT, Traumatic Brain Injury, Head Injury

SCHOLARONE™
Manuscripts

An evaluation of the impact of the NICE head injury guidelines on inpatient mortality from traumatic brain injury: an interrupted time series analysis

Carl Marincowitz[1] NIHR Doctoral Research Fellow, MB BChir, MSc, BA

Fiona E. Lecky[2] Professor, MB Ch B, FRCS, DA, MSc, PhD, FCEM

Victoria Allgar[3] Reader, BSc (Hons), PhD, C.Stat CSi

Trevor A. Sheldon[4] Professor, MSc, MSc, DSc, FMedSci

1. **Corresponding Author.** Hull York Medical School, Allam Medical Building, University of Hull, Hull HU6, 7RX, Fax: +44 (0) 1482 464705 Tel +44 (0) 870 1245500
Email: Carl.Marincowitz@hyms.ac.uk

2. School of Health and Related Research, University of Sheffield, Regent Court, 30 Regent Street, Sheffield, S1 4DA, UK, Fax: +44 (0)114 222 0749 Tel: (+44) (0)114 222 4345,
Email: f.e.lecky@sheffield.ac.uk

3 Hull York Medical School, John Hughlings Jackson Building, University of York, Heslington, York YO10 5DD Tel: 0870 124 5500, Email: Victoria.Allgar@hyms.ac.uk

4. Department of Health Sciences, University of York, Alcuin Research Resource Centre, Heslington, York, YO10 5DD, Tel +44 (0) 1904 321344, Fax: +44 (0) 1904 32 3433,
E-mail: Trevor.Sheldon@york.ac.uk

For peer review only

For peer review only - http://bmjopen.bmj.com/site/about/guidelines.xhtml

Abstract

Objective

To evaluate the impact of National Institute of Health and Care Excellence (NICE) head injury guidelines on deaths and hospital admissions caused by traumatic brain injury (TBI).

Setting

All hospitals in England between 1998-2017.

Participants

Patients admitted to hospital or who died up to 30 days following hospital admission with ICD coding indicating the reason for admission or death was TBI.

Intervention

An interrupted time series analysis was conducted with intervention points when each of the three guidelines was introduced. Analysis was stratified by guideline recommendation specific age groups (0-15, 16-64 and 65+).

Outcome Measures

The monthly population mortality and admission rates for TBI.

Study Design

An interrupted time series analysis using complete Office of National Statistics (ONS) cause of death data linked to Hospital Episode Statistics for inpatient admissions in England.

Results

The monthly TBI mortality and admission rates in the 65+ age group increased from 0.5 to 1.5 and 10 to 30 per 100 000 population respectively. The increasing mortality rate was unaffected by the introduction of any of the guidelines.

The introduction of the 2nd NICE Head Injury guideline was associated with a significant reduction in the monthly TBI mortality rate in the 16-64 age group (-0.005; 95% CI:-0.002 to -0.007).

For peer review only - http://bmjopen.bmj.com/site/about/guidelines.xhtml

In the 0-15 age group the TBI mortality rate fell from around 0.05 to 0.01 per 100 000 population, the trend was unaffected by the guidelines.

Conclusion

The introduction of NICE head injury guidelines was associated with reduced admitted TBI mortality rate after specialist care was recommended for severe TBI. The improvement was solely observed in 16-64 year olds.

The cause of the observed increased admission and mortality rates in those 65+ and potential treatments for TBI in this age group require further investigation.

Strengths and Limitations of this study:

This study is the first to use complete national data and the robust quasi-experimental method of interrupted time series analysis to evaluate the impact of the NICE head injury guidelines.

We adjusted our analysis for seasonality, autocorrelation and demographic changes using standard statistical techniques.

Inpatient mortality was assessed at a population level as national data on ED attendance for TBI was unavailable and the guidelines acted to change the admission threshold for TBI identified by CT imaging.

Keywords:

Traumatic Brain Injury, Head Injury, NICE Guidelines, Health Service Evaluation.

For peer review only

Background

There are approximately 2.5 million cases of Traumatic Brain Injury (TBI) (injury to the brain/ functional impairment due to external force) annually in the European Union and TBI is a leading cause of death and disability.[1] In higher income countries the epidemiology of TBI has changed from a condition predominantly of younger males resulting from high energy trauma, to older people caused by falls.[2]

One of the important health service challenges is identifying the small proportion of patients with life threatening TBI amongst the large number of patients who attend Emergency Departments (EDs) following head injury (blunt trauma to the head) and then ensure they receive specialist care, including neurosurgery, within a time critical period.[3] Previous research demonstrated correctly configured emergency health care systems are required to deliver optimal outcomes for patients with severe TBI.[1 4]

In England, since 2003, three NICE head injury guidelines have been introduced in order to improve the ED identification and subsequent management of TBI (Supplementary Material 1).[3 5-7]These would be expected to reduce TBI deaths and unnecessary hospital admissions. All three guidelines advocated increased CT imaging of head injured patients that present with a minimally impaired conscious level equivalent to a Glasgow Coma Scale (GCS) of 13-15. Increased costs from imaging were intended to be offset through reduced hospital admissions.[8] The 2007 guideline additionally recommended that patients with severe TBI should be managed in specialist neuroscience centres. At the time of implementation, concerns were raised that guideline recommendations were based on studies in subgroups and lacked supporting level 1 evidence .[4 9 10] Evaluation of the impact of these guidelines on national rates of TBI admissions and patient outcomes, is therefore needed.

We describe the first study to use complete national data and interrupted time series analysis to evaluate the impact of early TBI management guidelines on patient outcomes and admission rates for all severities of TBI.

For peer review only - http://bmjopen.bmj.com/site/about/guidelines.xhtml

Methods

Data set:

Hospital Episode Statistics (HES) are collected on all inpatients in England. The Office for National Statistics (ONS) has computerised ICD coding of cause of death information recorded on death certificates.

We used individual patient level HES data provided by NHS Digital on all emergency inpatient hospital admissions in England from April 1998 to April 2017. Reason for admission is recorded using ICD10 coding. For patients with ICD10 diagnostic codes: S00-S09 (indicating TBI) or T04.0 and T06.0 (crushing injury to the head) who died up to 30 days from discharge ONS cause of death was also provided.[11] ONS coding changed from ICD9 to ICD10 in 2001.

Deaths attributable to TBI:

Supplementary Material 2 summarises how deaths attributable to TBI over the study period were identified. 852646 deaths linked to admissions for head injury were identified by NHS Digital. We searched all cause of death fields for ICD9 and ICD10 codes defined by the CDC as indicating a death attributable to TBI (Table 1).[12] When any were present the death was coded as attributable to TBI. 34659 deaths attributable to TBI were identified and these were linked to their last recorded admission date as a proxy for when the injury and death occurred. This was not possible for 2862 patients. Neonatal deaths were excluded from analysis due to differences in cause of death coding.

For peer review only - http://bmjopen.bmj.com/site/about/guidelines.xhtml

Table 1: Annual numbers of deaths and admissions from TBI in England (source NHS digital)

Year	Admissions all age groups	Admissions 0-15	Admissions 16-64	Admissions 65+	Death all age groups	Deaths 0-15	Deaths 16-64	Deaths 65+
*1998	47820	17739	22348	7631	677	45	307	331
1999	63599	23848	29088	10553	964	71	446	453
2000	60001	21774	27793	10280	1076	69	492	525
2001	58497	21065	26553	10774	1105	62	519	532
2002	55941	19579	25808	10424	1178	46	508	634
2003	60336	19630	28405	12239	1294	51	521	729
2004	68662	20361	33298	14937	1342	49	568	734
2005	75391	20417	36832	18093	1484	43	606	840
2006	77333	19696	38005	19566	1570	49	610	917
2007	75219	18128	36473	20566	1665	39	624	1012
2008	74158	17481	34657	21938	1621	26	564	1036
2009	81218	18111	37178	25848	1739	35	603	1105
2010	81032	18008	35064	27856	1817	29	530	1260
2011	82093	18604	33989	29390	1879	35	500	1354
2012	76925	16453	30475	29901	2025	27	525	1474
2013	76429	15966	28983	31379	2204	27	497	1687
2014	79372	15535	28833	34890	2361	15	462	1886
2015	76648	13630	27517	35357	2610	18	493	2102
2016	74242	13120	25228	35488	2682	30	511	2145
*2017	16247	2619	5483	8037	504		79	420

*Data are from April 1998-March 2017, so 1998 and 2017 are part years and small number have been suppressed in accordance with NHS Digital guidance ICD9 definition TBI: 800, 801, 803, 804, 850, 851, 852, 853, 854, 905.0, 907.0 and 873 ICD10 definition TBI: S01.0−S01.9, S02.0, S02.1, S02.3, S02.7-S02.9, S04.0, S06.0−S06.9, S07.0, S07.1, S07.8, S07.9, S09.7−S09.9, T01.0, T02.0, T04.0, T06.0, T90.1, T90.2, T90.4, T90.5, T90.8 andT90.

For peer review only - http://bmjopen.bmj.com/site/about/guidelines.xhtml

Admissions attributable to TBI:

The same ICD10 codes used were used to identify patients admitted with TBI (Table 1).[12] We searched the primary diagnostic field in the inpatient HES data set for these codes and when present the reason for admission was coded as due to TBI. Data were cleaned and continuous inpatient spells (CIPS) were created for patients admitted with TBI using the approach outlined by Castelli, Laudicella and Street as this includes transfers within CIPS.[13] 1361537 CIPS for TBI were identified for 1245720 patients. Following cleaning, 402 CIPs were found to have admission dates prior to April 1998 and were excluded. Demographic and comorbidity information was calculated from the first consultant episode of a CIP. This included the monthly proportion of TBI admissions for males, monthly median age of admissions and mean monthly admission Charlson Comorbidity Index Score (using ICD10 code definitions and weights used to calculate the Summary Hospital-level Mortality Indicator (SHMI)).[14] This was compared to adjustment using a modified Charlson Comorbidity Index derived from the national (Trauma Audit and Research Network - TARN) trauma registry.[15]

Outcomes:

The monthly number of patients with deaths and admissions attributable to TBI between April 1998 and March 2017 was calculated. These were stratified into guideline specific age groups: 0-15, 16-64 and65+. Monthly mortality and admission rates were calculated per 100 000 population using Nomis ONS mid-year population estimates for England for each age group.[16]

Statistical Analysis:

A monthly time series of the mortality rate for TBI was plotted for the study period. Interrupted times series analysis (ITS) was conducted assessing the impact of the NICE guidelines using the ITSA package in STATA 14.[17] ITS analysis is a robust and increasingly used quasi-experimental method for the evaluation of health policies and allows causality to be attributed to an intervention introduced at a specific time point.[18]

The ITS model included three intervention time points corresponding to the introduction of each guidelines in: June 2003, September 2007 and January 2014. Analysis was conducted

For peer review only - http://bmjopen.bmj.com/site/about/guidelines.xhtml

separately for the 0-15, 16-64 and 65+ age groups. A segmented regression model predicting the mortality rate and hospital admission rate for TBI per 100 000 population in each age group per month was estimated.[18] A discontinuity in the gradient (level) or intercept (trend) of the fitted model was tested for at the time point when each guideline was introduced, and discontinuities in the model were measured in the monthly rate of the outcome per 100 000 population.

To adjust for potential changes in the composition of the TBI population that could possibly affect the risk of mortality a further ITS model predicting the TBI mortality rate adjusted for % male, median age and mean Charlson Comorbidity Index Score of patients admitted with TBI was fitted. Stratification by age group and intervention points were identical to the previous analysis.

In all analyses, autocorrelation of the residuals was assessed using the Durbin-Watson and Rho statistic. Throughout we used the Prais-Winsten transformation adjustment for auto-correlation due to improved fit of the model, deviation from a Durbin Watson statistic of 2 and a non-statistically significant Rho statistic.[18] Seasonality was assessed by introducing a dummy variable to the model in which winter months (December, January and February) were coded 1 and was included in the model when statistically significant.[19] To assess for possible implementation lags a sensitivity analysis was performed for all models in which the 12 months immediately following the introduction of a guideline were removed.[18]

Ethics

This study involved the analysis of anonymised routinely collected data and therefore NHS Research Ethics Committee review was not required. Data were stored and processed in accordance with NHS Digital guidance and data sharing agreement.

Patient and Public Involvement

The Hull and East Yorkshire NHS Trust Trans-Humber Consumer Research Panel and Hull branch of the Headway charity were consulted in the initial stages of developing the research questions addressed in this study. These patient groups highlighted that although national head injury guidelines seemed evidence based, there appeared to be little evidence to show they had achieved their aims.

For peer review only - http://bmjopen.bmj.com/site/about/guidelines.xhtml

Results

Mortality rate:

Table 1 shows the annual number and Supplementary Material 3 shows the annual rates of deaths and hospital admissions for TBI. The proportion of all TBI annual admissions for patients 65+ increased from 17% in 1998 to 48% in 2016 and the proportion of all TBI deaths in this age group increased from 49% to 78% over the same period. Figure 1 shows the monthly mortality rate per 100 000 population in each age group. Table 2 shows the results of the unadjusted interrupted time series assessing the impact of the NICE head injury guidelines. Deaths were more likely to occur in non-winter months in all age groups and so the figures are seasonally adjusted.

The trends in mortality rate and impact of the guidelines varied between age groups. In the 65+ age group the monthly TBI mortality rate increased from around 0.5 to over 1.5 per 100 000 population over the time period (Figure 1a). This was accompanied by an increase in the Charlson score of patients 65+ admitted with TBI (Supplementary Material 4). The NICE head injury guidelines were not associated with statistically significant changes in the level or trend in the mortality rate (Table 2). Subgroup analysis of patients aged 65-84 and 85+ showed that the increase in the mortality rate was greater in those 85+, from around 1 to over 6 per 100 000 population but similar changes were associated with the introduction of the guidelines to the whole 65+ population (Supplementary Material 5).

The 2nd guideline was found to be associated with a large reduction in mortality in the 16-64 age group (Figure 1b). Before the guideline, the monthly mortality rate was increasing but the introduction of the 2nd NICE guideline is associated with a reversal of this trend (-0.005; 95% CI:-0.002 to -0.007) (Table 2). The reduction in mortality appears to slow at the time of the introduction of the 3rd NICE guideline but this was not statistically significant. There was an increase in age of patients in the 16-64 age group admitted with TBI but no change in the Charlson comorbidity score over the period (Supplementary Material 4).

In the 0-15 age group the mortality rate fell continuously over the time period from around 0.05 to 0.01 per 100 000 population (Figure 1c). There were fewer monthly numbers of deaths and so more random variability in rates. None of the guidelines were associated with a statistically significant change in the level or trend in the mortality rate (Table 2),

For peer review only - http://bmjopen.bmj.com/site/about/guidelines.xhtml

though the high random variability meant we had lower statistical power to detect such changes as statistically significant.

Adjustment for the monthly median age, mean Charlson Score and proportion of male admissions for TBI did not materially alter the estimates associated with the introduction of guidelines in any of the age groups (Supplementary Material 6). In the 16-64 age group the estimate of the reversal in trend in mortality rate associated with the 2nd Guideline, -0.006 (95% CI:-0.008 to -0.003), was similar to the unadjusted analysis. The levelling off in the rate of reduction in mortality in the 16-64 age group associated with the 3rd NICE guideline became marginally statistically significant, although the estimate is similar, 0.003 (95% CI: 0.00005 to 0.007). No adjustment was made for the standard Charlson score in the paediatric and 16-64 age groups as it did not change over time. The monthly mean trauma modified Charlson score in the 16-64 age group increased slightly from 0 to 1 and adjustment for this increased the estimated size of reversal in mortality trend associated with the 2nd NICE guideline , -0.008 (95% CI:-0.01 to -0.005), (Supplementary Material 4). The sensitivity analysis for the effect of implementation lags did not affect the estimates associated with the introduction of any guideline (Supplementary Material 7).

Admission Rate:

Figure 2 shows the trends in monthly TBI admissions stratified by age group and Table 3 presents estimates of the change in admission rate associated with the introduction of each Head Injury guideline iteration. The admission rate increased threefold (from around 10 per 100 000 to 30 per 100 000) in the 65+ age group. The introduction of the 1st NICE guideline is associated with large increasing trends in monthly TBI admissions per 100 000 population in both the 65+ age group (0.17: 95% CI: 0.11 to 0.22) and the 16-64 age group (0.25: 95% CI: 0.16 to 0.34) (Table 3).[20] The subsequent 2 guidelines are associated with significant reductions in this trend and admission rates level off following the 3rd guideline in the 65+ age group (Table 3 and Figure 2a). In the 16-64 age group, the TBI admissions trend reverses and declines after the 2nd NICE guideline (-0.33: 95% CI: -0.42 to -0.25) (Table 3 and Figure 2b).

For peer review only - http://bmjopen.bmj.com/site/about/guidelines.xhtml

In the 0-15 age group TBI admissions steadily fall over the study period from around 20 per 100 000 to 10 per 100 000 (Figure 2c), and is unaffected by the introduction of the guidelines (Table 3).

A sensitivity analysis for implementation lags in which the 12 months following the introduction of a guideline were removed from the analysis did not materially change the estimates associated with the introduction of the guidelines in any age group (Supplementary Material 8).

For peer review only

For peer review only - http://bmjopen.bmj.com/site/about/guidelines.xhtml

Table 2: The impact of the NICE head injury guidelines on monthly TBI mortality rate per 100 000 population

Age Band	Winter Effect	Initial Trend	1st NICE Guideline	2nd NICE Guideline	3rd NICE Guideline	Durbin-Watson Statistic
**65+**	-0.1<br>(95% CI: -0.16 to -0.04)<br>P<0.01	0.005<br>(95% CI: 0.002 to 0.008)<br>P<0.01	**Change level:**<br>-0.034<br>(95% CI:-0.21 to 0.14)<br>P=0.71	**Change level:**<br>-0.1<br>(95% CI: -0.27 to 0.07)<br>P=0.24	**Change level:**<br>0.13<br>(95% CI:-0.04 to 0.32)<br>P=0.14	Untransformed<br>1.57<br>Prais-Winsten<br>1.86
			**Change trend:**<br>0.002<br>(95% CI:-0.003 to 0.008)<br>P=0.43	**Change trend:**<br>0.0004<br>(95% CI: -0.005 to 0.006)<br>P=0.89	**Change trend:**<br>-0.005<br>(95% CI:-0.01 to 0.002)<br>P=0.14	
**16-64**	-0.1<br>(95% CI: -0.13 to -0.06)<br>P<0.01	0.002<br>(95% CI:0.001 to 0.004)<br>P<0.01	**Change level:**<br>-0.03<br>(95% CI: -0.11 to 0.06)<br>P=0.57	**Change level:**<br>-0.06<br>(95% CI:-0.15 to 0.003)<br>P=0.17	**Change level:**<br>0.005<br>(95% CI:-0.087 to 0.096)<br>P=0.92	Untransformed<br>1.79<br>Prais-Winsten<br>1.95
			**Change trend:**<br>-0.00002<br>(95% CI: -0.003 to 0.003)<br>P=0.99	**Change trend:**<br>*-0.005*<br>*(95% CI:-0.007 to -0.002)*<br>*P<0.01*	**Change trend:**<br>0.002<br>(95% CI:-0.002 to 0.005)<br>P=0.38	
**0-15**	-0.01<br>(95% CI:-0.01 to -0.003)<br>P<0.01	-0.0003<br>(95% CI: -0.0005 to -0.00001)<br>P=0.04	**Change level:**<br>0.001<br>(95% CI: -0.01 to 0.01)<br>P= 0.18	**Change level:**<br>-0.0021<br>(95% CI: -0.01 to 0.01)<br>P=0.74	**Change level:**<br>-0.01<br>(95% CI:-0.03 to 0.002)<br>P=0.09	Untransformed<br>2.12<br>Prais-Winsten<br>1.99
			**Change trend:**<br>0.00004<br>(95% CI:-0.0004 to 0.0004)<br>P=0.17	**Change trend**<br>0.0001<br>(95% CI:-0.0003 to 0.0005)<br>P=0.58	**Change trend:**<br>0.0005<br>(95% CI: -0.00005 to 0.001)<br>P=0.08	

For peer review only

For peer review only - http://bmjopen.bmj.com/site/about/guidelines.xhtml

Table 3: The impact of the NICE head injury guidelines on monthly TBI hospital admission rate per 100 000 population

Age Band	Winter Effect	Initial Trend	1st NICE Guideline	2nd NICE Guideline	3rd NICE Guideline	Durbin-Watson Statistic
**65+**	-0.44<br>(95% CI: -0.94 to 0.06)<br>P=0.08	0.01<br>(95% CI: -0.02 to 0.05)<br>P=0.42	***Change level:***<br>*1.71*<br>*(95% CI:-0.01 to 3.44)*<br>*P=0.05*	**Change level:**<br>-0.4<br>(95% CI: -2.08 to 1.27)<br>P=0.64	**Change level:**<br>0.04<br>(95% CI:-1.73 to 1.82)<br>P=0.96	Untransformed 1.1<br>Prais-Winsten 2.09
			Change trend:<br>*0.17*<br>*(95% CI: 0.11 to 0.23)*<br>*P<0.01*	***Change trend:***<br>*-0.08*<br>*(95% CI: -0.13 to -0.03)*<br>*P<0.01*	***Change trend:***<br>*-0.13*<br>*(95% CI:-0.2 to -0.05)*<br>*P<0.01*	
**16-64**	-1.92<br>(95% CI: -2.77 to -1.07)<br>P<0.01	-0.08<br>(95% CI: -0.13 to -0.02)<br>P<0.01	***Change level:***<br>*5.21*<br>*(95% CI: 2.53 to 7.89)*<br>*P<0.01*	***Change level:***<br>*-2.76*<br>*(95% CI:-5.35 to -0.16)*<br>*P=0.04*	**Change level:**<br>-0.72<br>(95% CI: -3.49 to 2.03)<br>P=0.61	Untransformed 1.35<br>Prais-Winsten 2.11
			Change trend:<br>*0.25*<br>*(95% CI: 0.16 to 0.34)*<br>*P<0.01*	***Change trend:***<br>*-0.33*<br>*(95% CI: -0.42 to -0.25)*<br>*P<0.01*	**Change trend:**<br>0.02<br>(95% CI:-0.09 to 0.13)<br>P=0.73	
**0-15**	-2.87<br>(95% CI: -3.40 to -2.34)<br>P<0.01	-0.06<br>(95% CI:-0.11 to -0.01)<br>P=0.03	**Change level:**<br>1.3<br>(95% CI: -1.03 to 3.63)<br>P= 0.27	**Change level:**<br>0.19<br>(95% CI: -2.09 to 2.47)<br>P=0.87	**Change level:**<br>0.34<br>(95% CI:-2.03 to 2.72)<br>P=0.78	Untransformed 1.07<br>Prais-Winsten 1.70
			**Change trend:**<br>0.02<br>(95% CI: -0.07 to 0.11)<br>P=0.61	**Change trend**<br>-0.005<br>(95% CI: -0.08 to 0.08)<br>P=0.91	**Change trend:**<br>-0.08<br>(95% CI: -0.19 to 0.03)<br>P=0.17	

For peer review only

For peer review only - http://bmjopen.bmj.com/site/about/guidelines.xhtml

Discussion:

Summary:

To our knowledge this is the first study to use national population based data and interrupted time series analysis to evaluate the impact of the NICE head injury guidelines in England. The 2nd NICE guideline was associated with a reduction in the admitted TBI mortality rate in the 16-64 age group at a population level (Table 2). We found no other impact on mortality associated with the three guideline iterations.

There was a continual and significant increase in TBI mortality and admission rates in the 65+ age group and a contrasting falling trend in mortality and admission rates in children. (Figure 1 and Figure 2). Both trends began before the introduction of the NICE guidelines and were not significantly affected by any of the three iterations. In both the 16-64 and 65+ age groups there was a large increase in hospital admissions for TBI at the time the 1st NICE guideline was introduced (Figure 2).

Increased imaging was intended to reduce hospital admissions by reducing diagnostic uncertainty but the 1st NICE guideline coincided with the introduction of the 4-hour target.[8 20] We have shown, using Scottish data assessing the impact of similar (SIGN) guidelines (introduced at a different time to the 4-hour target), that the 4-hour target acted to undermine this reduction and cause a large increase in hospital admissions.[21] No mortality benefit was found at the time of the introduction of the 4-hour target in England.

Later guidelines were associated with a reduction in hospital admissions rates in both adult populations assessed (Figure 2). Further increases in CT imaging may have reduced hospital admissions, as intended, by reducing diagnostic uncertainty in the ED, without the distorting effect of the 4-hour target introduction.

Strengths:

We used complete national data for England to assess the impact of the NICE head injury guidelines on mortality after admission for TBI at a population level. We have used individual level patient data to define TBI deaths and admissions. We controlled for seasonal factors and auto-correlation using established techniques.[18] We used mid-year population estimates to adjust for changes in the demography of England’s population.

For peer review only - http://bmjopen.bmj.com/site/about/guidelines.xhtml

Weaknesses

Ideally, we would have estimated the impact of the guidelines on case fatality, as this better measures the impact on the population at risk. The impact on case fatality of those attending ED with TBI could not be estimated because ED data were not collected until 2007. The impact on case fatality of those admitted with TBI could be estimated but because the guidelines resulted in changes in admissions policies and rates, the rate of deaths per admission is difficult to interpret. Instead we analysed the impact on the population TBI mortality rate, as this represents the best available unbiased measure of the guidelines' impact. This outcome may be affected by changes in the underlying population TBI rate that we are unable to account for, although annual attendances to the ED for head injury gradually smoothly increased over the study period (Supplementary Material 9). We were unable to assess possible impact on disability or other patient reported outcomes, as they are not routinely collected.

ONS linked HES data is based on routinely collected administrative data; these can suffer from poor accuracy of injury coding.[22] This is particularly likely in older patients with multimorbidity (TARN – personal communication). Random poor coding, as opposed to a discrete and systematic change in coding practice, however, is unlikely to account for discontinuities observed at the specific time points of interest but may make a discontinuity harder to detect. ONS changed from ICD9 to ICD10 coding of cause of death in 2001. A sensitivity analysis excluding the period that used ICD9 coding did not materially alter the estimate of the reversal in mortality trend associated with the 2nd guideline in the 16-64 age group. We are unaware of other significant changes to coding practice in the HES or ONS data during the study period. The limitations of HES data mean that mortality rates could not be adjusted for anatomical severity of brain injury and presenting physiology. However, adjustment for other known predictors of TBI mortality did not materially change estimates associated with the introduction of the guidelines and we are unaware of evidence that the prevalence of these factors changed at the point individual guidelines were introduced.

The impact of guidelines is limited by how well they are implemented. The NICE head injury guidelines have been found to be well implemented,[23] albeit with less compliance to CT imaging recommendations in the paediatric population.[23 24] There is evidence that each

For peer review only - http://bmjopen.bmj.com/site/about/guidelines.xhtml

guideline caused step increases in CT head scanning in other age groups, particularly in those 65+.[10 25]

The reconfiguration of the trauma network in England in 2012 is a co-intervention which could affect the TBI mortality rate.[26] However, we found no impact on mortality associated with the 2014 NICE guideline introduced around this time. Apart from the introduction of the 4-hour ED admissions target in 2004, we are unaware of any other co-interventions that occurred around the time the NICE guidelines were introduced which could account for the observed discontinuities in mortality and hospital admissions.

Comparison to previous literature:

Few previous studies assess the impact of the NICE head injury guidelines (see Table 4).[9] A cohort study using Trauma Audit and Research Network (TARN) national registry data suggested the increased rate of transfer of severe TBI patients to neuroscience centres between 2003-2009 was associated with a halving of severe TBI case fatality.[4] TARN data were only collected at approximately half of hospitals in England until 2012 and on a TBI patient subset. Our study, using complete national data and interrupted time series analysis, found that guideline recommended management of patients with severe injuries in specialist centres only reduced the mortality rate in the 16-64 age group.

A paediatric study analysing English HES data from 2000-2011 found a reduction in annual mortality during admissions for head injury after the introduction of 2007 NICE guideline.[24] We found a fall in the mortality rate over the study period in the 0-16 age group which was unaffected by any guideline. This may reflect the greater number of data points we used to estimate the time dependent model and use of interrupted time series analysis to assess for discontinuities. We also used ONS linked HES data to identify deaths directly attributable to TBI up to 30 days following discharge. The observed decreasing mortality and admission rates may reflect improving clinical management or a reduction in TBI in this age group due to improving road traffic safety during the study period. [24]

An economic evaluation of the NICE guidelines found them to be cost effective due to a reduction in hospital admissions predicted from early single centre studies and improved outcomes.[8 10] A subsequent study using HES data found hospital admissions for head injury increased after the introduction of the 1st NICE guideline.[11] The similar increase in adult TBI

For peer review only - http://bmjopen.bmj.com/site/about/guidelines.xhtml

admissions we found associated with the 1st NICE guideline probably is due the 4-hour target.[21] We found subsequent NICE guidelines improved outcomes and reduced hospital admissions in the 16-64 but not the 65+ age group, implying the guidelines were less cost-effective in older patients.

For peer review only

For peer review only - http://bmjopen.bmj.com/site/about/guidelines.xhtml

Table 4: Comparison to previous literature

	Previous Study		Current study
	Study population	Findings	Findings
Fuller et al 2009[4]	TARN eligible patients at TARN submitting hospitals (approx. 50% England) between 2003-2009	From the period 2004 onwards as the proportion of patients with TBI transferred and managed in neuroscience centres increased and the risk adjusted mortality rate for TBI fell.	Complete national data for all hospital in England.<br><br>A reversal in trend in the mortality rate in the 16-64 age group when the 2nd NICE guideline recommending management of patients with severe injuries in specialist centres was introduced.
Marlow et al 2015[24]	Patients aged <16 with ICD10 codes indicating head injury admitted to hospitals in England between 2000 and 2011.	Assessed the annual rate of inpatient deaths (all-cause mortality) for patients admitted with ICD10 codes indicating head injury, Found the death rate fell across the time period, but there was only a statistically significant reduction in the death rate after the 2007 NICE head injury guideline.	The inpatient TBI mortality rate (as indicated by coding of death certificates) for patients aged<16 fell from 1998-2017 and was unaffected by the introduction of the NICE guidelines.
The Trauma and Audit Research Network Report: Major Trauma in Older People[25]	TARN eligible patients at TARN submitting hospitals between 2005 and 2014 (all hospitals in England by 2014)	A large increase in major trauma, including TBI, in patients 65+, disproportionate to UK population demographic changes.<br><br>Hypothesised due to increased case ascertainment due to more liberal CT imaging.	We found a large increase in the admission rate for TBI in those 65+ from 10 per 100 000 population to 30 per 100 000 population between 2002 and the point the 3rd NICE guideline was introduced in 2014.

For peer review only

For peer review only - http://bmjopen.bmj.com/site/about/guidelines.xhtml

Other studies using TARN data have found increases in TBI in patients 65+ disproportionate to population changes and it has been suggested that better case ascertainment due to increased CT imaging in older patients may account for this.[2 25] The large increase in admissions for TBI for those 65+ we found at the point the first guideline was introduced, although boosted by the 4-hour target, supports this (Fig 2a and Table 3). The lack of improvement in admitted TBI mortality in older patients following the 2nd NICE guideline could either result from unequal access to treatment in specialist centres or such treatment appearing to be less effective in this group. The TARN older persons audit found patients aged over 60 to be less likely to be manged in Major Trauma Centres (where neurosurgical units are located in England) and more likely to experience delays in investigation and be treated by junior staff.[25] However, other studies have found age to be an independent predictor of mortality that is unaffected by early treatment in neuroscience centres.[27 28]

We are unaware of comparable national evaluations of the impact of head injury guidelines. Evaluations of international Brain Trauma Foundation guidelines, particularly in the USA, have utilised evidence from single centre studies or subsets of patients.[23 29 30] Evaluation of their national impact has not been possible due to their variable implementation.[23 30]

Implications:

We found evidence that only the second NICE head injury guideline was associated with a change in population based TBI mortality. This guideline contained a recommendation for increased management of severe TBI in specialist centres. Much research has focused on determining which head injured patients require CT imaging.[3 31] Increased diagnosis by itself, however, without a change in subsequent patient management was not associated with improved outcomes in our analysis. Even if apparent increases in TBI rates in older patients reflect the identification of previously unmet need, this still represents a significant health service challenge. Routine ICD coding of TBI is particularly problematic in this group and robust evaluation of treatment in specialist neuroscience centres and other interventions may be required to improve outcomes in older TBI patients. The UK, however, has one of the lowest numbers of ICU beds per population in Europe and when the 2007 guideline recommendation was made concerns were raised about the system meeting demand.[9 32] Research needs to focus on how to best configure and ration specialist services for TBI in a transparent and evidenced based way.

For peer review only - http://bmjopen.bmj.com/site/about/guidelines.xhtml

Conclusion

This first national evaluation suggests that the introduction of the second NICE head injury guideline was associated with a reduction in the admitted TBI mortality rate in the 16-64 age group and a reduction in TBI admissions in England. The guidelines were not associated with significant changes in the secular trend for TBI admissions and subsequent mortality in children and those aged 65+. Research is needed to identify clinically and cost-effective management approaches for TBI in older patients.

Acknowledgements:

The Hull and East Yorkshire NHS Trust Trans-Humber Consumer Research Panel and Hull branch of the Headway charity helped develop the research questions addressed in this study.

Authors' contributions:

This idea for the study was conceived by Carl Marincowitz with help from Trevor Sheldon, Fiona Lecky and Victoria Allgar. The analysis was completed by Carl Marincowitz with specialist advice regarding interrupted time series analysis from Trevor Sheldon and Victoria Allgar. Fiona Lecky provided specialist advice regarding the clinical context and interpretation of the results. All authors read and approved the final manuscript.

Data Sharing

Access to the individual level Office of National Statistics linked Hospital Episode Statistics is subject to a data sharing agreement with NHS Digital that limits access to the data to named members of the research team.

Competing interests:

The authors declare that they have no competing interests.

Funding:

Carl Marincowitz is funded by a National Institute for Health Research Doctoral Fellowship (DRF-2016-09-086). This study presents independent research funded by the National Institute for Health Research (NIHR). The views expressed are those of the author(s) and not necessarily those of the NHS, the NIHR or the Department of Health.

For peer review only - http://bmjopen.bmj.com/site/about/guidelines.xhtml

For peer review only

Figures:

Figure 1: The impact of the NICE Head Injury Guidelines on monthly TBI mortality rate per 100 000 population

Figure 2: The impact of the NICE Head Injury Guidelines on monthly TBI hospital admissions per 100 000 population

For peer review only

For peer review only - http://bmjopen.bmj.com/site/about/guidelines.xhtml

1. Maas AIR, Menon DK, Adelson PD, et al. Traumatic brain injury: integrated approaches to improve prevention, clinical care, and research. *Lancet Neurol* 2017;16(12):987-1048. doi: 10.1016/S1474-4422(17)30371-X [published Online First: 2017/11/11]
2. Lawrence T, Helmy A, Bouamra O, et al. Traumatic brain injury in England and Wales: prospective audit of epidemiology, complications and standardised mortality. *BMJ Open* 2016;6(11):e012197. doi: 10.1136/bmjopen-2016-012197
3. NICE. National Clinical Guidance Centre. (2014). CG 176 Head Injury Triage, assessment, investigation and early management of head injury in children, young people and adults. National Institute for Health and Care Excellence. In: NICE, ed. UK: DOH, 2014.
4. Fuller G, Bouamra O, Woodford M, et al. Temporal trends in head injury outcomes from 2003 to 2009 in England and Wales. *Br J Neurosurg* 2011;25(3):414-21. doi: 10.3109/02688697.2011.570882
5. NICE. National Clinical Guidance Centre. (2007). Guideline CG56: Head injury: Triage, assessment, investigation and early management of head injury in infants, children and adults. National Institute for Health and Care Excellence. UK: DOH; 2007.
6. NICE. National Clinical Guidance Centre. (2003). Clinical guideline 4: Head injury: triage, assessment, investigation and early management of head injury in infants, children and adults. National Institute for Health and Care Excellence. UK: DOH; 2003.
7. Hodgkinson S, Pollit V, Sharpin C, et al. Early management of head injury: summary of updated NICE guidance. *BMJ* 2014;348:g104. doi: 10.1136/bmj.g104 [published Online First: 2014/01/24]
8. Pandor A, Goodacre S, Harnan S, et al. Diagnostic management strategies for adults and children with minor head injury: a systematic review and an economic evaluation. *Health Technol Assess* 2011;15(27):1-202. doi: 10.3310/hta15270
9. Barratt H, Wilson M, Moore F, et al. The implications of the NICE guidelines on neurosurgical management for all severe head injuries: systematic review. *Emerg Med J* 2010;27(3):173-8. doi: 10.1136/emj.2009.075382 [published Online First: 2010/03/23]
10. Hassan Z, Smith M, Littlewood S, et al. Head injuries: a study evaluating the impact of the NICE head injury guidelines. *Emerg Med J* 2005;22(12):845-9. doi: 10.1136/emj.2004.021717
11. Goodacre S. Hospital admissions with head injury following publication of NICE guidance. *Emerg Med J* 2008;25(9):556-7. doi: 10.1136/emj.2007.055723
12. Coronado VG, Xu L, Basavaraju SV, et al. Surveillance for traumatic brain injury-related deaths--United States, 1997-2007. *MMWR Surveill Summ* 2011;60(5):1-32. [published Online First: 2011/05/06]
13. Castelli A, Laudicella M, Street A. Measuring NHS Output growth. *CHE research paper* 2008; **043**: University of York. http://www.york.ac.uk/inst/che/pdf/rp43.pdf.
14. Campbell MJ, Jacques RM, Fotheringham J, et al. Developing a summary hospital mortality index: retrospective analysis in English hospitals over five years. *BMJ* 2012;344:e1001. doi: 10.1136/bmj.e1001 [published Online First: 2012/03/03]
15. Bouamra O, Jacques R, Edwards A, et al. Prediction modelling for trauma using comorbidity and 'true' 30-day outcome. *Emerg Med J* 2015;32(12):933-8. doi: 10.1136/emermed-2015-205176
16. Office of National Statistics https://www.nomisweb.co.uk/articles/924.aspx.
17. Linden A. Conducting interrupted time-series analysis for single- and multiple-group comparisons. *The Stata Journal* 2015;15(2):480-500.
18. Lopez Bernal J, Cummins S, Gasparrini A. Interrupted time series regression for the evaluation of public health interventions: a tutorial. *Int J Epidemiol* 2016 doi: 10.1093/ije/dyw098 [published Online First: 2016/06/11]
19. Hamilton I, Lloyd C, Hewitt C, et al. Effect of reclassification of cannabis on hospital admissions for cannabis psychosis: a time series analysis. *Int J Drug Policy* 2014;25(1):151-6. doi: 10.1016/j.drugpo.2013.05.016 [published Online First: 2013/07/23]

For peer review only - http://bmjopen.bmj.com/site/about/guidelines.xhtml

20. Comptroller General. Improving Emergency Care in England. *National Audit Office* 2004; HC 1075 Session 2003-2004. https://www.nao.org.uk/wp-content/uploads/2004/10/03041075.pdf
21. Marincowitz C. The impact of the SIGN head injury guidelines and NHS 4-hour Emergency Target on hospital admissions for head injury in Scotland: An Interrupted Times Series. *BMJ Open* 2018 In Press
22. Hand DJ. Statistical challenges of administrative and transaction data. *J R Statist Soc A* 2018 (pre-publication presented at The Royal Statistical Society);181(Part 3):1-24.
23. Cnossen MC, Scholten AC, Lingsma HF, et al. Adherence to Guidelines in Adult Patients with Traumatic Brain Injury: A Living Systematic Review. *J Neurotrauma* 2016 doi: 10.1089/neu.2015.4121 [published Online First: 2015/10/04]
24. Marlow R, Mytton J, Maconochie IK, et al. Trends in admission and death rates due to paediatric head injury in England, 2000-2011. *Arch Dis Child* 2015;100(12):1136-40. doi: 10.1136/archdischild-2015-308615
25. *The Trauma and Audit Research Network Report* 2017. Major Trauma in Older People. https://www.tarn.ac.uk/content/downloads/3793/Major%20Trauma%20in%20Older%20People%202017.pdf
26. Yiannoullou P, Hall C, Newton K, et al. A review of the management of blunt splenic trauma in England and Wales: have regional trauma networks influenced management strategies and outcomes? *Ann R Coll Surg Engl* 2017;99(1):63-69. doi: 10.1308/rcsann.2016.0325
27. Fountain DM, Kolias AG, Lecky FE, et al. Survival Trends After Surgery for Acute Subdural Hematoma in Adults Over a 20-year Period. *Ann Surg* 2017;265(3):590-96. doi: 10.1097/SLA.0000000000001682
28. Utomo WK, Gabbe BJ, Simpson PM, et al. Predictors of in-hospital mortality and 6-month functional outcomes in older adults after moderate to severe traumatic brain injury. *Injury* 2009;40(9):973-7. doi: 10.1016/j.injury.2009.05.034 [published Online First: 2009/06/23]
29. Faul M, Wald MM, Rutland-Brown W, et al. Using a cost-benefit analysis to estimate outcomes of a clinical treatment guideline: testing theBrain Trauma Foundation guidelines for the treatment of severe traumatic brain injury. *J Trauma* 2007;63(6):1271-8. doi: 10.1097/TA.0b013e3181493080 [published Online First: 2008/01/24]
30. Lee JC, Rittenhouse K, Bupp K, et al. An analysis of Brain Trauma Foundation traumatic brain injury guideline compliance and patient outcome. *Injury* 2015;46(5):854-8. doi: 10.1016/j.injury.2014.12.023 [published Online First: 2015/02/11]
31. Foks KA, van den Brand CL, Lingsma HF, et al. External validation of computed tomography decision rules for minor head injury: prospective, multicentre cohort study in the Netherlands. *BMJ* 2018;362:k3527. doi: 10.1136/bmj.k3527 [published Online First: 2018/08/26]
32. Murthy S, Wunsch H. Clinical review: International comparisons in critical care - lessons learned. *Crit Care* 2012;16(2):218. doi: 10.1186/cc11140 [published Online First: 2012/05/02]

For peer review only

For peer review only - http://bmjopen.bmj.com/site/about/guidelines.xhtml

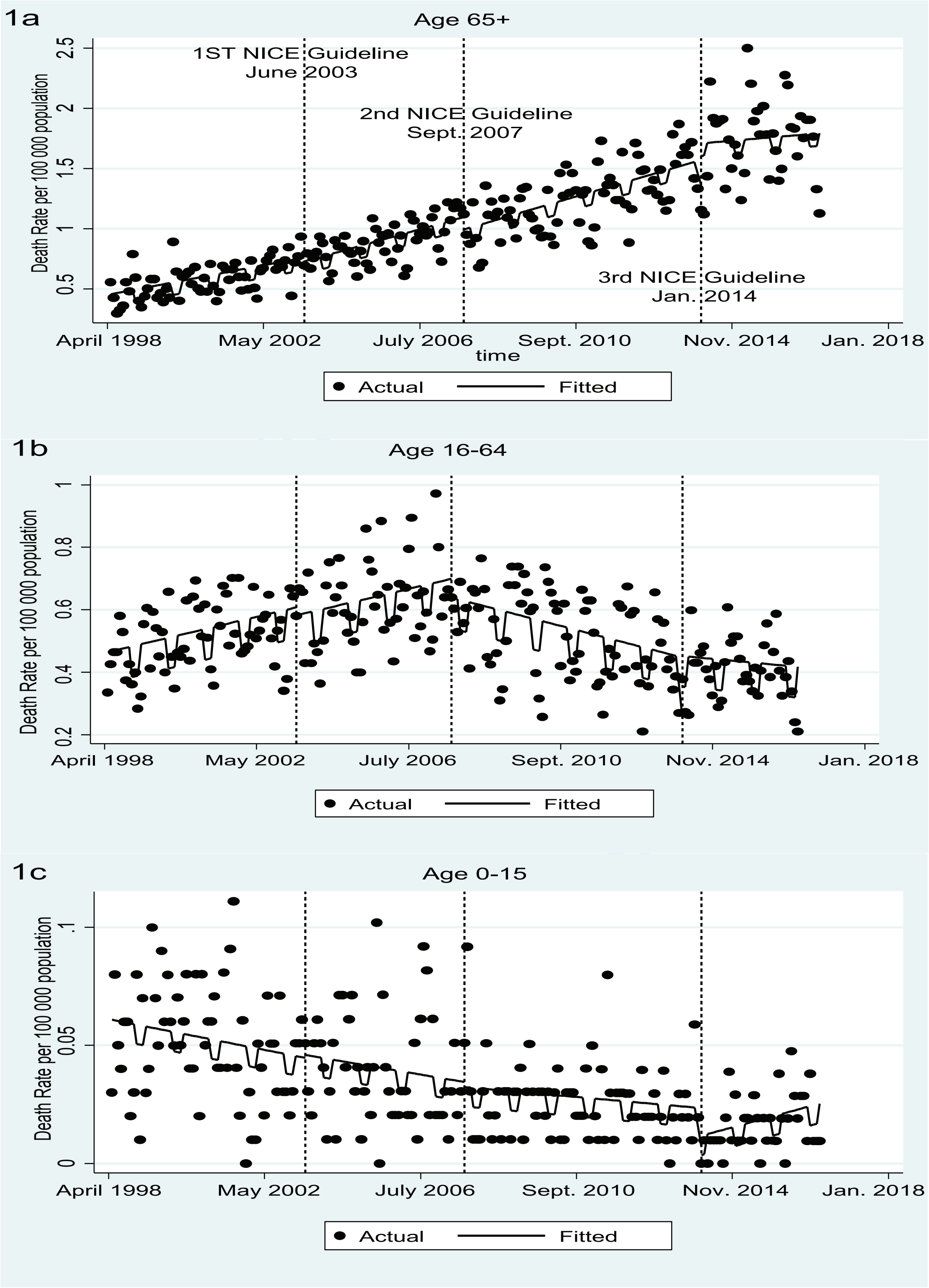
1a
Age 65+
1ST NICE Guideline
June 2003
2nd NICE Guideline
Sept. 2007
3rd NICE Guideline
Jan. 2014
Death Rate per 100 000 population
2.5
2
1.5
1
0.5
April 1998
May 2002
July 2006
Sept. 2010
Nov. 2014
Jan. 2018
time
Actual
Fitted
1b
Age 16-64
Death Rate per 100 000 population
1
0.8
0.6
0.4
0.2
April 1998
May 2002
July 2006
Sept. 2010
Nov. 2014
Jan. 2018
Actual
Fitted
1c
Age 0-15
Death Rate per 100 000 population
.1
0.05
0
April 1998
May 2002
July 2006
Sept. 2010
Nov. 2014
Jan. 2018
Actual
Fitted

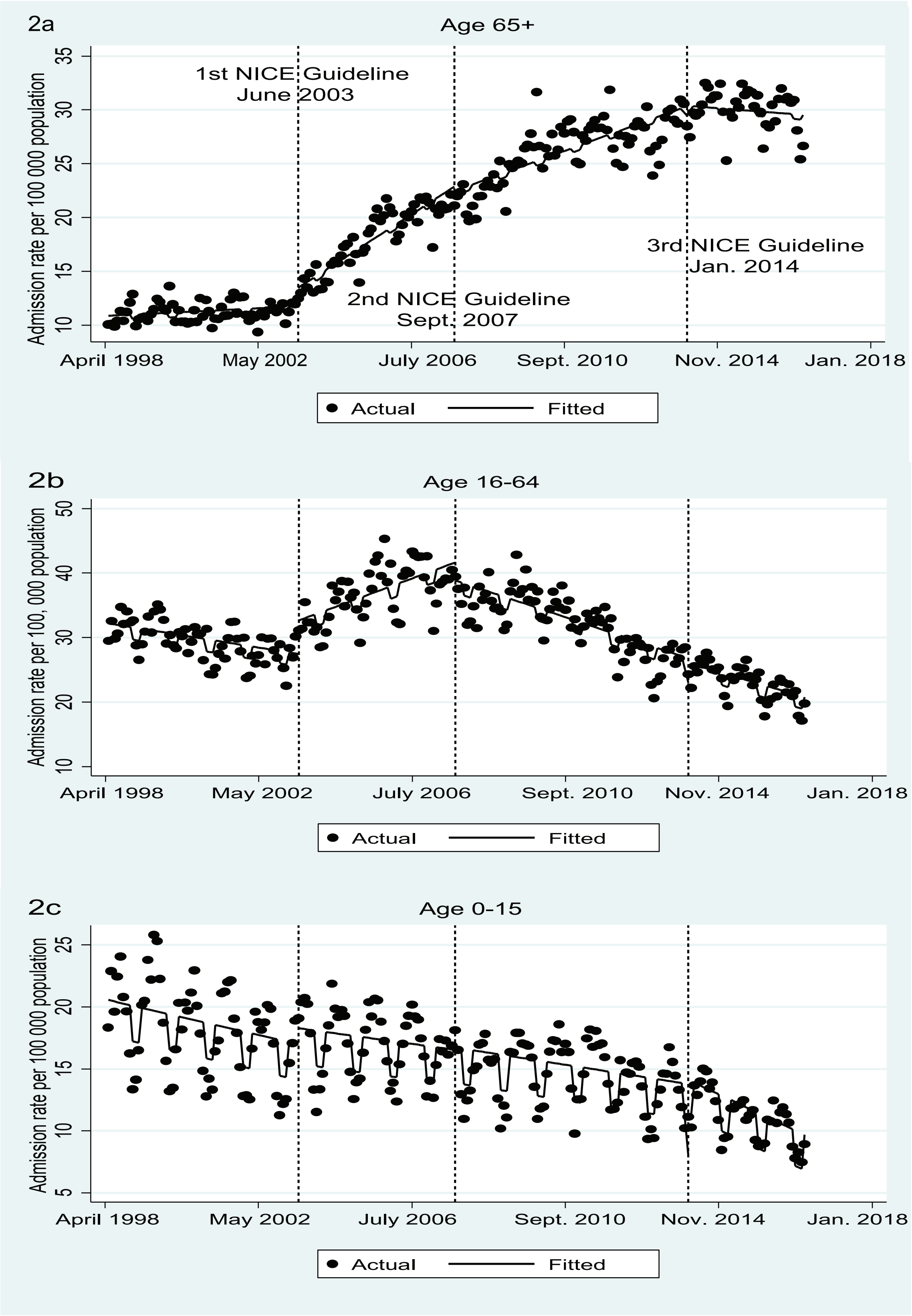
2a
Age 65+
Admission rate per 100 000 population
1st NICE Guideline
June 2003
2nd NICE Guideline
Sept. 2007
3rd NICE Guideline
Jan. 2014
April 1998
May 2002
July 2006
Sept. 2010
Nov. 2014
Jan. 2018
Actual
Fitted
2b
Age 16-64
Admission rate per 100, 000 population
2c
Age 0-15
Admission rate per 100 000 population

Supplementary Material 1: Key Features of the NICE Head Injury Guidelines

Policy	Time of Introduction	Key Features
1st NICE Head Injury Guideline	June 2003	Indication for CT imaging (referenced directly from 2003 Guideline):<br><br>- GCS less than 13 on initial assessment in the emergency department.<br>- GCS equal to 13 or 14 at 2 hours after the injury on assessment in the emergency department.<br>- Suspected open or depressed skull fracture.<br>- Any sign of basal skull fracture (haemotympanum, 'panda' eyes, cerebrospinal fluid otorrhoea, Battle's sign).<br>- Post-traumatic seizure.<br>- Focal neurological deficit.<br>- More than one episode of vomiting.<br>- Amnesia for greater than 30 minutes of events before impact.<br><br>CT should also recommended in patients with any of the following risk factors, provided they have experienced some loss of consciousness or amnesia since the injury:<br>-Age greater than or equal to 65 years.<br>-Coagulopathy (history of bleeding, clotting disorder, current treatment with warfarin).<br>-Dangerous mechanism of injury (a pedestrian struck by a motor vehicle, an occupant ejected from a motor vehicle or a fall from a height of greater than 1 metre or five stairs).
2nd NICE Head Injury Guideline	September 2007	Specialist management (referenced directly from 2007 Guideline):<br><br>Local guidelines on the transfer of patients with head injuries should be drawn up between the referring hospital trusts, the neuroscience unit and the local ambulance service, and should recognise that:<br>-Transfer would benefit all patients with serious head injuries (GCS<9), irrespective of the need for neurosurgery

For peer review only

		-If transfer of those who do not require neurosurgery is not possible, ongoing liaison with the neuroscience unit over clinical management is essential.<br><br>Indications Immediate CT scanning (adult):<br>- Glasgow coma score <13 on initial assessment in the emergency department<br>- Glasgow coma score <15 two hours after the injury on assessment in the emergency department<br>- Suspected open or depressed skull fracture<br>- Any sign of basal skull fracture<br>- Post-traumatic seizure<br>- Focal neurological deficit<br>- One or more episodes of vomiting<br>- Amnesia for events more than 30 minutes before impact.<br><br>Indications Immediate CT scanning (<16 years):<br>- Age over 1 year: Glasgow coma score <14 on assessment in the emergency department<br>- Age under 1 year: Glasgow coma score paediatric <15 on assessment in the emergency department<br>- Age under 1 year and presence of bruise, swelling, or laceration (>5 cm) on the head<br>- Clinical suspicion of non-accidental injury<br>- Post-traumatic seizure but no history of epilepsy<br>- Abnormal drowsiness<br>- Suspected open or depressed skull injury, or tense fontanelle<br>- Any sign of basal skull fracture<br>- Focal neurological deficit<br>- Three or more discrete episodes of vomiting<br>- Amnesia (antegrade or retrograde) lasting more than five minutes.
3rd NICE Head Injury Guideline	January 2014	Referenced directly from 3rd NICE Guidelines<br><br>Indications CT scanning < 1 hour (adult):<br>- GCS<13/15<br>- GCS <15 after2 hours from injury<br>- Suspected open or depressed skull fracture<br>- Any sign of basal skull fracture

		- Post-traumatic seizure<br>- Focal neurological deficit<br>- One or more episodes of vomiting<br>Indications CT scanning < 8 hours (adult):<br>- Patient taking warfarin<br>- LOC or amnesia + dangerous mechanism/age 65+/history of bleeding/clotting disorder<br>- Amnesia for events more than 30 minutes before impact.<br><br>Indications CT scanning < 1 hour (<16 years) if 1 of:<br>- Suspicion of non-accidental injury<br>- Post-traumatic seizure but no history of epilepsy.<br>- On initial emergency department assessment, GCS less than 14, or for children under 1 year GCS (paediatric) less than 15.<br>- At 2 hours after the injury, GCS less than 15.<br>- Suspected open or depressed skull fracture or tense fontanelle.<br>- Any sign of basal skull fracture (haemotympanum, 'panda' eyes, cerebrospinal fluid leakage from the ear or nose, Battle's sign).<br>- Focal neurological deficit.<br>- For children under 1 year, presence of bruise, swelling or laceration of more than 5 cm on the head.<br>Indications CT scanning < 1 hour (<16 years) if 2 or more of:<br>- Loss of consciousness lasting more than 5 minutes (witnessed).<br>- Abnormal drowsiness.<br>- Three or more discrete episodes of vomiting.<br>- Dangerous mechanism of injury<br>- Amnesia (antegrade or retrograde) lasting more than 5 minutes[4].<br>If only 1 above risk factor observe for 4 hours post injury if during observation develop any risk factor below for CT within 1 hour<br>- GCS less than 15.<br>- Further vomiting.<br>- A further episode of abnormal drowsiness.<br>If taking warfarin for CT within 8 hours.

For peer review only

Supplementary Material 2: Flow diagram of identification of deaths attributable to TBI used in analysis

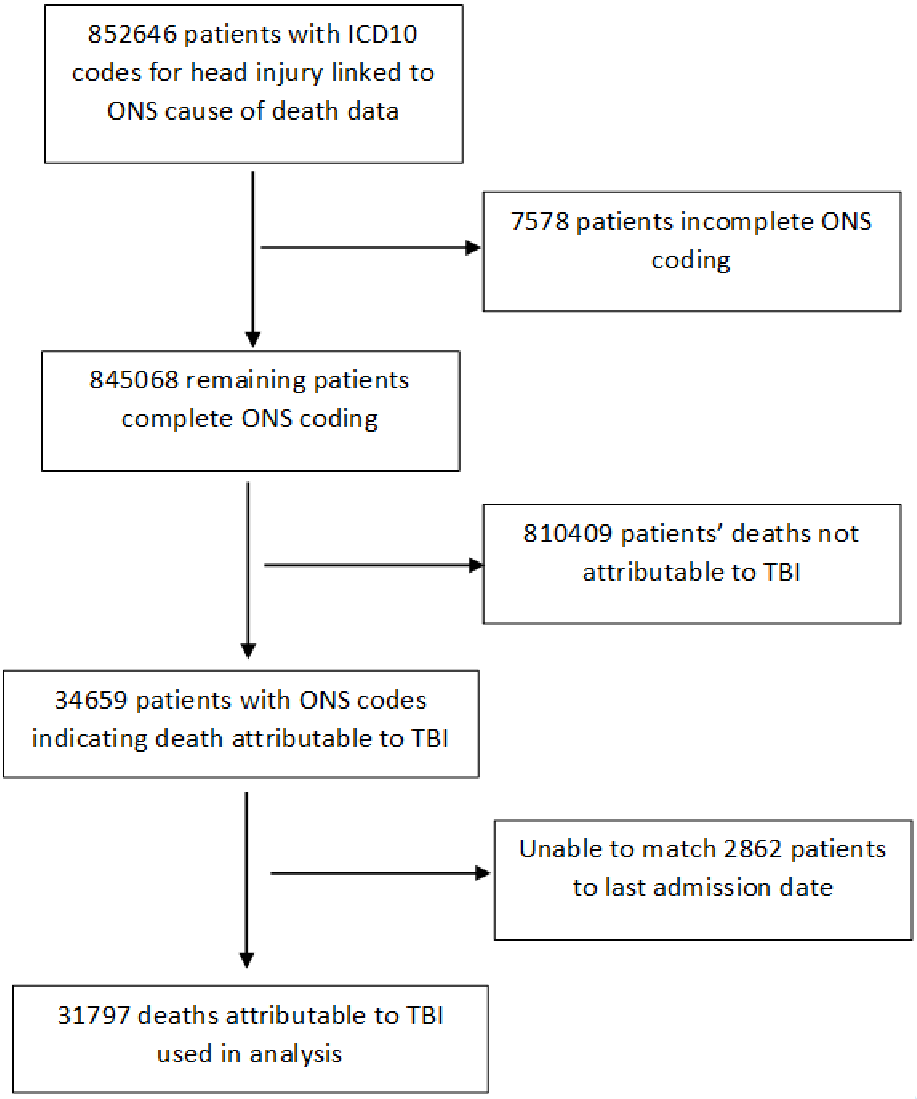

Supplementary Material 3: Annual rate of deaths and admissions for TBI per 100 000 population in England (source NHS digital)

Year	Admissions 0-15	Admissions 16-64	Admissions 65+	Deaths 0-15	Deaths 16-64	Deaths 65+
*1998	177	288	98	0.45	3.96	4.27
1999	238	375	136	0.71	5.75	5.84
2000	218	357	132	0.69	6.32	6.75
2001	213	339	137	0.63	6.62	6.79
2002	198	327	132	0.47	6.44	8.04
2003	199	358	154	0.52	6.57	9.19
2004	207	417	187	0.50	7.12	9.20
2005	208	459	225	0.44	7.55	10.46
2006	201	472	242	0.50	7.57	11.38
2007	185	449	253	0.40	7.68	12.46
2008	177	420	266	0.26	6.84	12.56
2009	183	443	308	0.35	7.18	13.15
2010	181	409	325	0.29	6.19	14.71
2011	185	389	337	0.35	5.73	15.51
2012	162	336	330	0.27	5.80	16.28
2013	156	311	337	0.26	5.34	18.13
2014	151	302	366	0.15	4.84	19.77
2015	131	283	364	0.17	5.08	21.64
2016	125	255	359	0.28	5.17	21.70

*Available data are from April 1998-March 2017, so 1998 is a part year and 2017 is not reported

For peer review only

Supplementary Material 4: Monthly admission characteristics of patients with TBI:

Median age (65 and over)

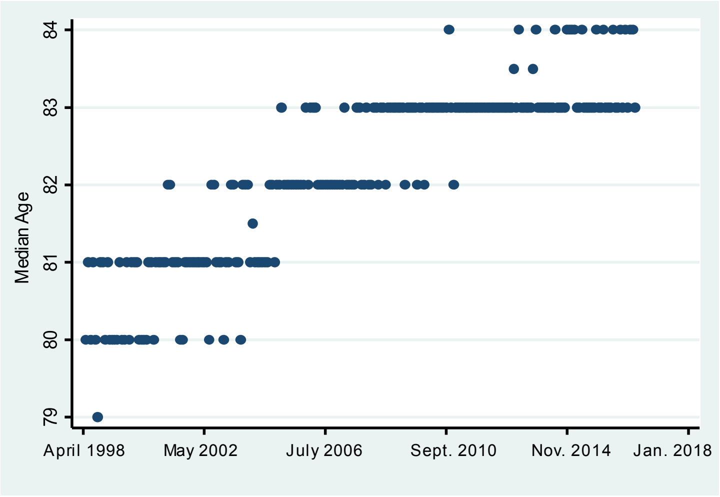

Proportion Male (65 and over)

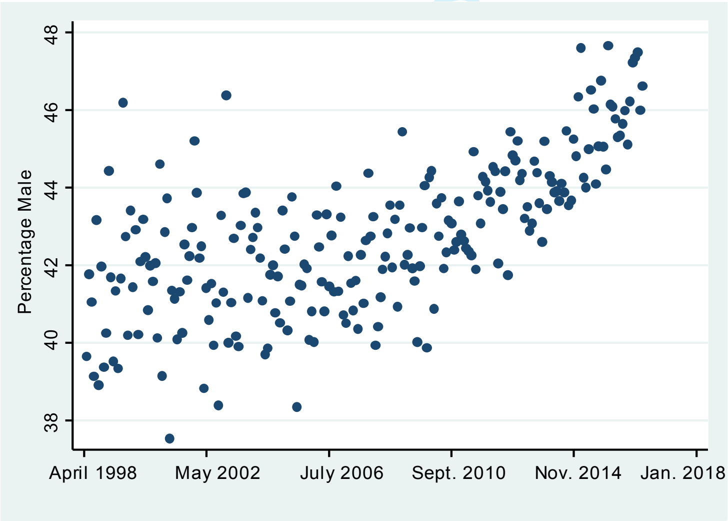

Mean Monthly Charlson Score (65 and over)

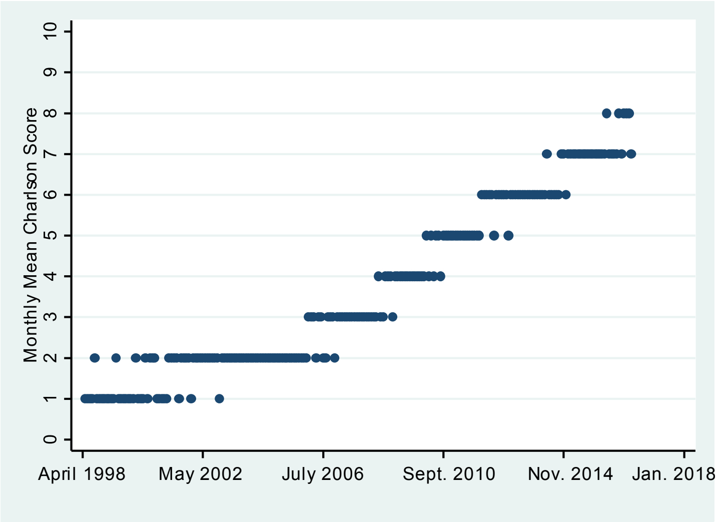

Mean Monthly Trauma Modified Charlson Score (65 and over)

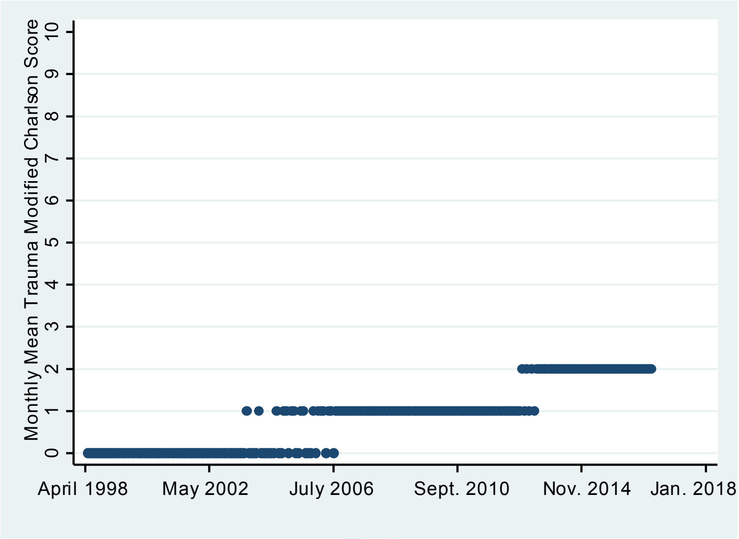

Median age (16-64)

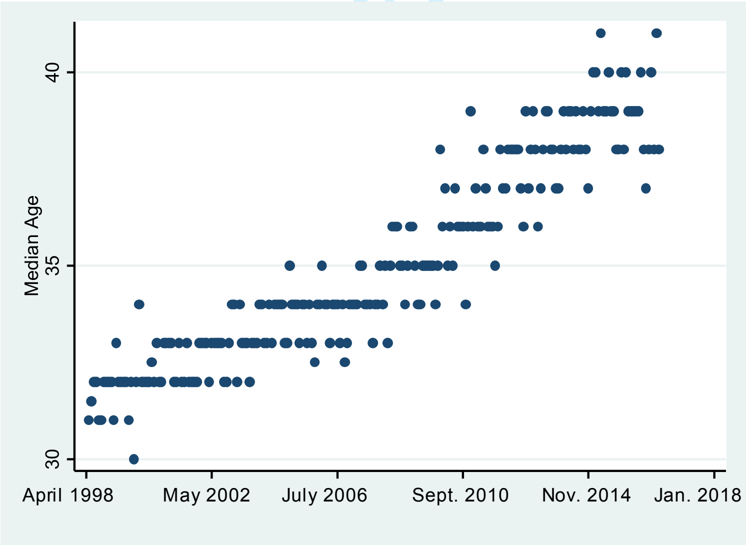

Proportion Male (16-64)

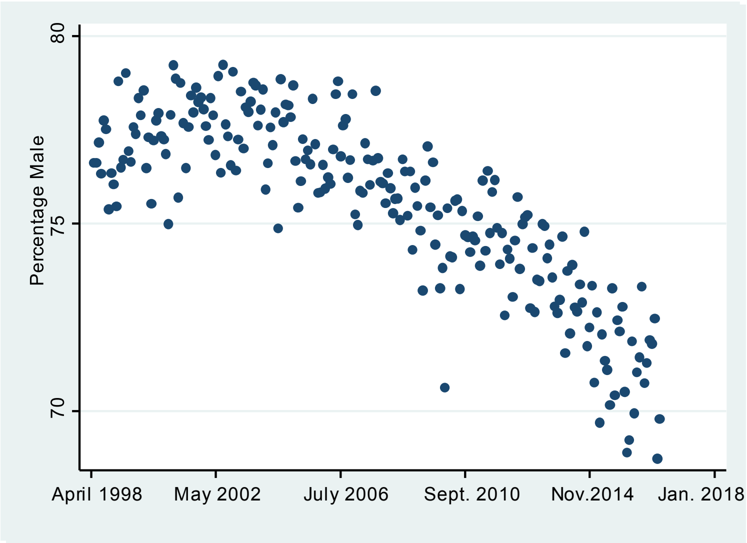

Mean Monthly Standard Charlson Score (16-64)

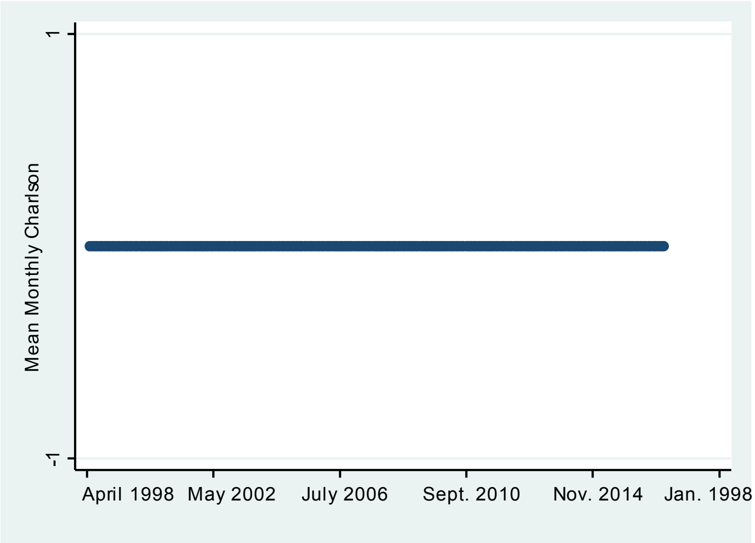

Mean Monthly Trauma Modified Charlson Score (16-64)

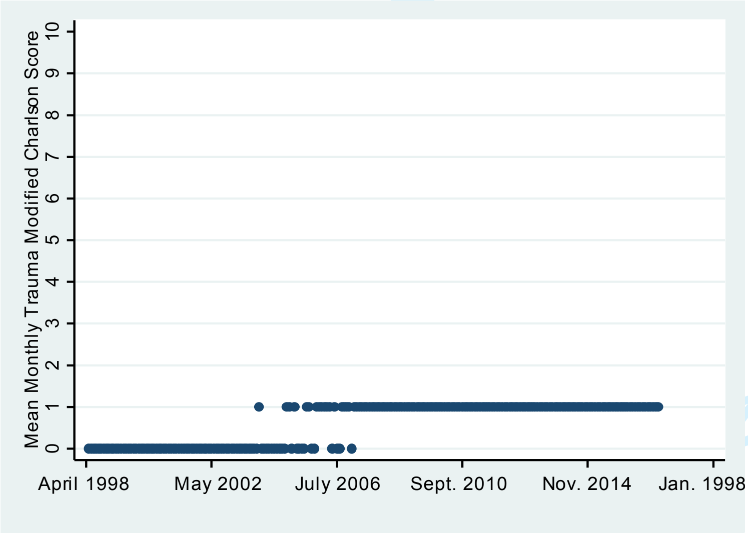

Median age (0-15)

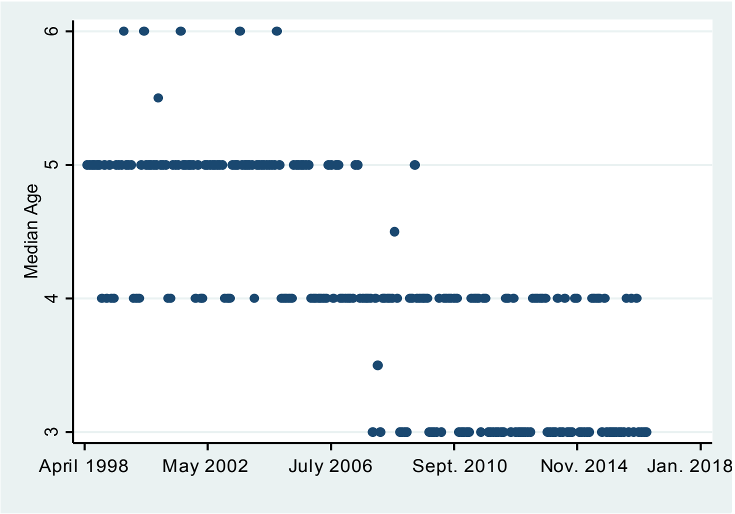

Proportion Male (0-15)

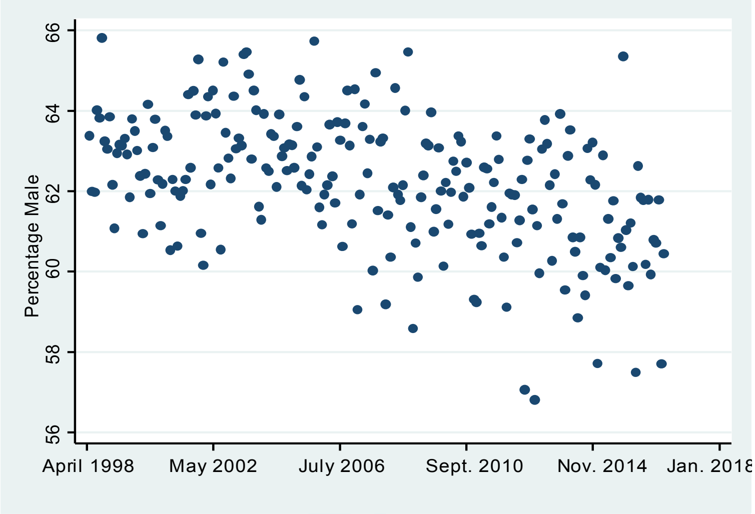

Mean Monthly Standard Charlson Score (0-15)

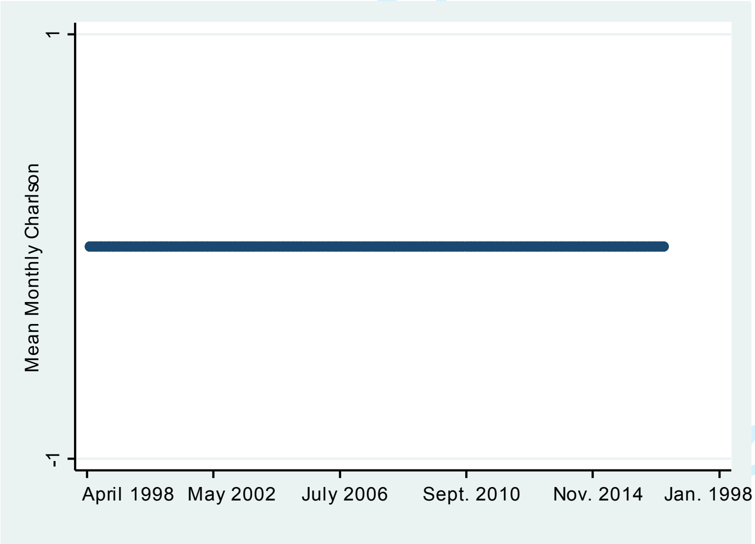

Mean Monthly Trauma Modified Charlson Score (0-15)

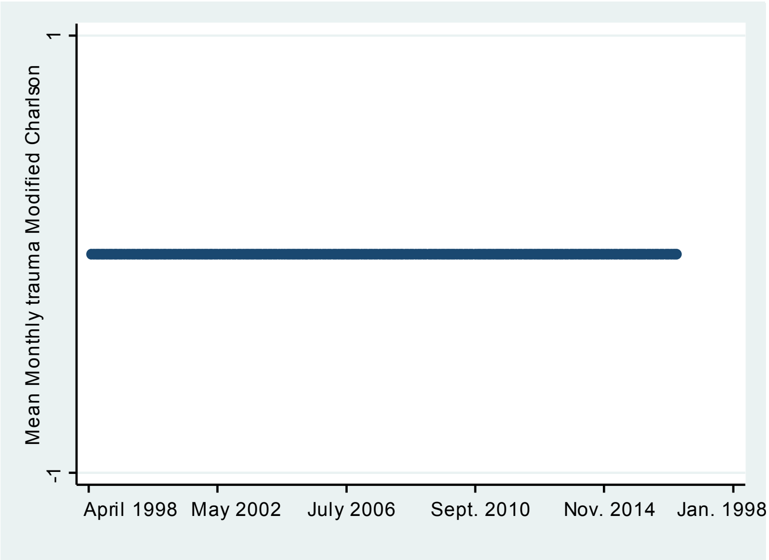

Supplementary Material 5: Subgroup analysis of effect of the NICE guidelines on patients aged 65 deaths per 100, 000 population

**Age Band**	**Winter Effect**	**Initial Trend**	**1st NICE Guideline**	**2nd NICE Guideline**	**3rd NICE Guideline**	**Durbin-Watson Statistic**
**65-84**	-0.06<br>(95% CI: -0.1 to -0.02)<br>P=0.01	0.003<br>(95% CI: 0.001 to 0.005)<br>P=0.006	**Change level:**<br>-0.02<br>(95% CI:-0.13 to 0.1)<br>P=0.78	**Change level:**<br>-0.07<br>(95% CI: -0.19 to 0.04)<br>P=0.21	**Change level:**<br>0.09<br>(95% CI:-0.03 to 0.21)<br>P=0.15	Untransformed 1.62<br>Prais-Winsten 1.89
			**Change trend:**<br>0.001<br>(95% CI:-0.002 to 0.005)<br>P=0.51	**Change trend:**<br>-0.001<br>(95% CI: -0.005 to 0.002)<br>P=0.44	**Change trend:**<br>-0.003<br>(95% CI:-0.008 to 0.001)<br>P=0.16	
**85+**	-0.46<br>(95% CI: -0.73 to -0.2)<br>P<0.01	0.02<br>(95% CI: 0.01 to 0.03)<br>P=0.01	**Change level:**<br>-0.03<br>(95% CI:-0.7 to 0.7)<br>P=0.92	**Change level:**<br>-0.38<br>(95% CI: -1.05 to 0.29)<br>P=0.27	**Change level:**<br>0.54<br>(95% CI:-0.18 to 1.26)<br>P=0.14	Untransformed 1.68<br>Prais-Winsten 1.91
			**Change trend:**<br>0.001<br>(95% CI:-0.02 to 0.02)<br>P=0.9	**Change trend:**<br>0.02<br>(95% CI: -0.001 to 0.04)<br>P=0.65	**Change trend:**<br>-0.02<br>(95% CI:-0.05 to 0.01)<br>P=0.15	

For peer review only

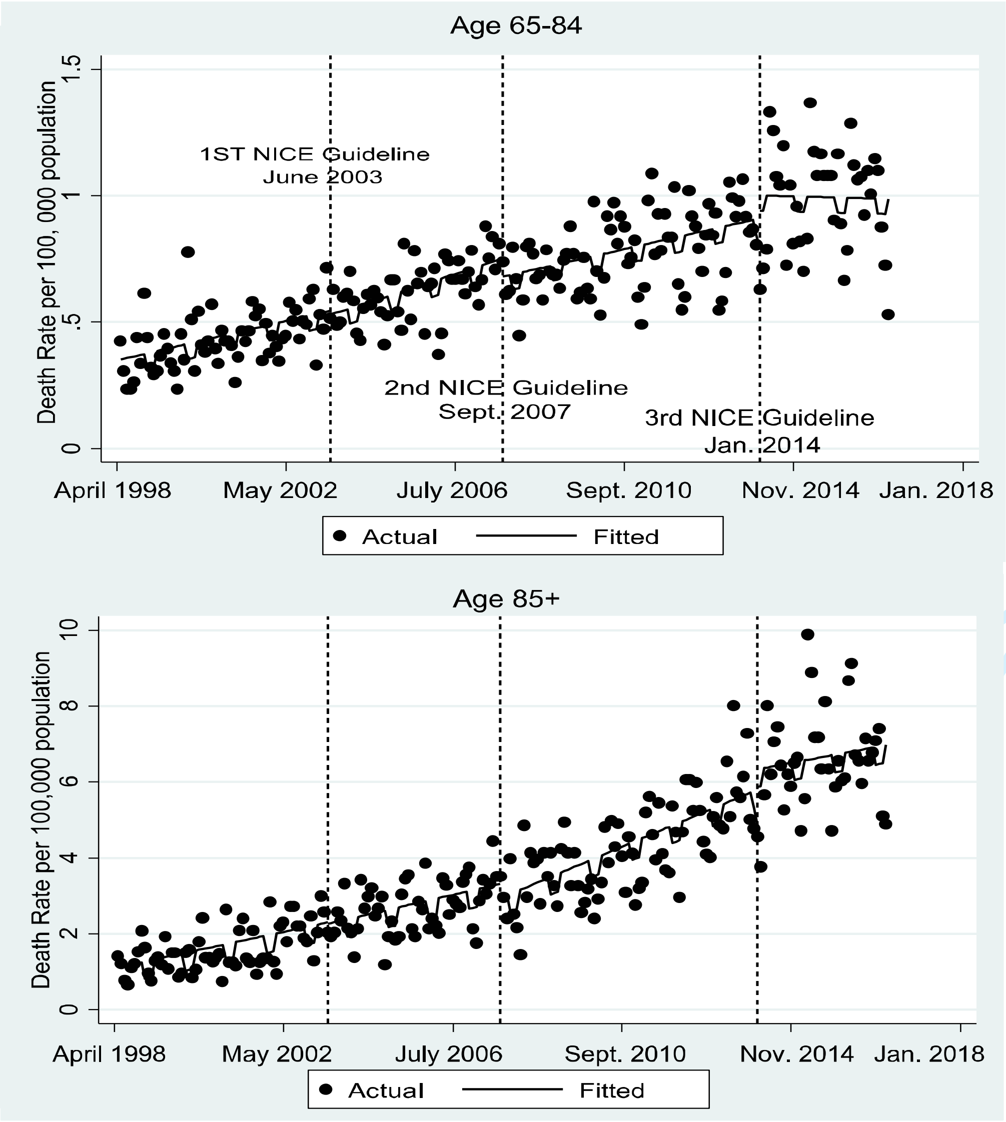
Age 65-84
Death Rate per 100, 000 population
0
.5
1
1.5
1ST NICE Guideline
June 2003
2nd NICE Guideline
Sept. 2007
3rd NICE Guideline
Jan. 2014
April 1998
May 2002
July 2006
Sept. 2010
Nov. 2014
Jan. 2018
Actual
Fitted
Age 85+
Death Rate per 100,000 population
0
2
4
6
8
10
April 1998
May 2002
July 2006
Sept. 2010
Nov. 2014
Jan. 2018
Actual
Fitted

Supplementary Material 6: The impact of the NICE head injury guidelines on monthly TBI mortality rate per 100 000 population adjusted for age, sex and comorbidity

**Age Band**	**Winter Effect**	**Initial Trend**	**Median Age**	**Proportion Male**	**Charlson Score**	**1st NICE Guideline**	**2nd NICE Guideline**	**3rd NICE Guideline**	**Durbin-Watson Statistic**
**65+**	-0.1<br>(95% CI: -0.17 to -0.04)<br>P<0.01	0.006<br>(95% CI: 0.002 to 0.009)<br>P<0.01	-0.03<br>(95% CI: -0.09 to 0.02)<br>P=0.25	0.03<br>(95% CI: -1.80 to 1.87)<br>P=0.97	0.00003<br>(95% CI: -0.07 to 0.07)<br>P>0.99	**Change level:**<br>-0.04<br>(95% CI:-0.22 to 0.14)<br>P=0.69<br>**Change trend:**<br>0.003<br>(95% CI: -0.003 to 0.008)<br>P=0.39	**Change level:**<br>-0.1<br>(95% CI: -0.28 to 0.07)<br>P=0.25<br>**Change trend:**<br>-0.0002<br>(95% CI:-0.006 to 0.005)<br>P=0.95	**Change level:**<br>0.14<br>(95% CI:-0.05 to 0.32)<br>P=0.15<br>**Change trend:**<br>-0.005<br>(95% CI:-0.01 to 0.002)<br>P=0.14	Untransformed 1.56<br>Prais-Winsten 1.86
**16-64**	-0.12<br>(95% CI: -0.15 to -0.09)<br>P<0.01	0.001<br>(95% CI: -0.0003 to 0.003)<br>P=0.1	0.03<br>(95% CI: 0.01 to 0.05)<br>P<0.01	1.40<br>(95% CI:0.1 to 2.69)<br>P=0.04	Not adjusted for as no change over time period.	**Change level:**<br>-0.03<br>(95% CI:-0.11 to 0.06)<br>P=0.52<br>**Change trend:**<br>0.0001<br>(95% CI: -0.002 to 0.004)<br>P=0.52	**Change level:**<br>0.06<br>(95% CI:-0.14 to 0.02)<br>P=0.15<br>**Change trend:**<br>*-0.006*<br>*(95% CI: -0.008 to -0.003)*<br>*P<0.01*	**Change level:**<br>-0.0004<br>(95% CI: -0.085 to 0.085)<br>P=0.99<br>**Change trend:**<br>0.003<br>(95% CI: 0.00005 to 0.007)<br>P=0.047	Untransformed 1.89<br>Prais-Winsten 1.98
**0-15**	-0.01<br>(95% CI: -0.01 to 0.001)<br>P=0.09	-0.0002<br>(95% CI: -0.0005 to -0.00002)<br>P=0.04	0.006<br>(95% CI: 0.00002 to 0.01) P=0.049	-0.09<br>(95% CI:-0.28 to 0.09)<br>P=0.32	Not adjusted for as no change over time period.	**Change level:**<br>0.0001<br>(95% CI: -0.01 to 0.01) P= 0.99<br>**Change trend:**<br>0.0001<br>(95% CI:-0.0003 to 0.0005) P=0.58	**Change level:**<br>-0.0004<br>(95% CI: -0.01 to 0.01) P=0.95<br>**Change trend**<br>0.00005<br>(95% CI:-0.0003 to 0.0004)<br>P=0.81	**Change level:**<br>-0.01<br>(95% CI:-0.03 to 0.001) P=0.08<br>**Change trend:**<br>0.0004<br>(95% CI: -0.00007 to 0.001) P=0.09	Untransformed 2.19<br>Prais-Winsten 1.99

For peer review only

Supplementary Material 7: Sensitivity analysis of implementation lags on the impact of the NICE head injury guidelines on deaths per 100 000 population

Age Band	Winter Effect	Initial Trend	1st NICE Guideline	2nd NICE Guideline	3rd NICE Guideline	Durbin-Watson Statistic
**65+**	-0.11<br>(95% CI: -0.18 to-0.04)<br>P<0.01	0.005<br>(95% CI: 0.001 to 0.008)<br>P<0.01	**Change level:**<br>-0.007<br>(95% CI:-0.2 to 0.19)<br>P=0.95	**Change level:**<br>-0.05<br>(95% CI: -0.25 to 0.14)<br>P=0.60	**Change level:**<br>0.13<br>(95% CI:-0.06 to 0.33)<br>P=0.18	Untransformed 1.56<br>Prais-Winsten 1.86
			**Change trend:**<br>0.005<br>(95% CI:-0.003 to 0.012)<br>P=0.24	**Change trend:**<br>-0.0018<br>(95% CI: -0.01 to 0.006)<br>P=0.65	**Change trend:**<br>-0.006<br>(95% CI:-0.01 to 0.002)<br>P=0.16	
**16-64**	-0.1<br>(95% CI: -0.14 to -0.06)<br>P<0.01	0.002<br>(95% CI:0.001 to 0.004)<br>P<0.01	**Change level:**<br>0.01<br>(95% CI: -0.08 to 0.11)<br>P=0.78	**Change level:**<br>0.06<br>(95% CI:-0.15 to 0.003)<br>P=0.11	**Change level:**<br>0.006<br>(95% CI: -0.09 to 0.1)<br>P=0.91	Untransformed 1.75<br>Prais-Winsten 1.94
			**Change trend:**<br>-0.001<br>(95% CI: -0.004 to 0.003)<br>P=0.77	**Change trend:**<br>-0.004<br>(95% CI:-0.008 to -0.001)<br>P=0.03	**Change trend:**<br>0.002<br>(95% CI:-0.002 to 0.005)<br>P=0.41	
**0-15**	-0.01<br>(95%CI:-0.01 to -0.001)<br>P=0.02	-0.0003<br>(95% CI: -0.0005 to -0.00001)<br>P=0.03	**Change level:**<br>0.001<br>(95% CI: -0.01 to 0.01)<br>P= 0.88	**Change level:**<br>-0.001<br>(95% CI: -0.01 to 0.01)<br>P=0.93	**Change level:**<br>-0.01<br>(95% CI:-0.03 to 0.002)<br>P=0.097	Untransformed 2.18<br>Prais-Winsten 1.98
			**Change trend:**<br>0.00007<br>(95% CI: -0.0006 to 0.0005)<br>P=0.80	**Change trend**<br>0.0002<br>(95% CI: -0.0003 to 0.0007)<br>P=0.47	**Change trend:**<br>0.0005<br>(95% CI: -0.00003 to 0.001)<br>P=0.07	

For peer review only

Supplementary Material 8: Sensitivity analysis of implementation lags on the impact of the NICE head injury guidelines on admissions per 100 000 population

Age Band	Winter Effect	Initial Trend	1st NICE Guideline	2nd NICE Guideline	3rd NICE Guideline	Durbin-Watson Statistic
**65+**	-0.51<br>(95% CI: -1.05 to 0.04)<br>P=0.07	0.02<br>(95% CI -0.02 to 0.05)<br>P=0.31	**Change level:**<br>3.88<br>(95% CI: 2.11 to 5.66)<br>P<0.01<br>**Change trend:**<br>0.17<br>(95% CI: 0.09 to 0.24)<br>P<0.01	**Change level:**<br>1.71<br>(95% CI: -0.08 to 3.5)<br>P=0.06<br>**Change trend:**<br>-0.1<br>(95% CI: -0.17 to -0.03)<br>P=0.01	**Change level:**<br>0.6<br>(95% CI:-1.17 to 2.36)<br>P=0.51<br>**Change trend:**<br>-0.1<br>(95% CI:-0.18 to -0.03)<br>P=0.01	Untransformed 1.24<br>Prais-Winsten 2.05
**16-64**	-2.16<br>(95% CI: -3.03 to -1.28)<br>P<0.01	-0.08<br>(95% CI:-0.12 to -0.03)<br>P<0.01	**Change level:**<br>8.6<br>(95% CI: 6 to 11.2)<br>P<0.01<br>**Change trend:**<br>0.2<br>(95% CI: 0.09 to 0.3)<br>P<0.01	**Change level:**<br>-2.22<br>(95% CI:-4.84 to 0.4)<br>P=0.1<br>**Change trend:**<br>-0.32<br>(95% CI: -0.42 to -0.21)<br>P<0.01	**Change level:**<br>0.25<br>(95% CI:-2.33 to 2.84)<br>P=0.85<br>**Change trend:**<br>0.06<br>(95% CI:-0.05 to 0.16)<br>P=0.29	Untransformed 1.49<br>Prais-Winsten 2.06
**0-15**	-2.93<br>(95% CI: -3.49 to -2.38)<br>P<0.01	-0.06<br>(95%CI:-0.11 to -0.01)<br>P=0.02	**Change level:**<br>1.16<br>(95% CI: -1.22 to 3.54)<br>P= 0.34<br>**Change trend:**<br>0.02<br>(95% CI: -0.1 to 0.13)<br>P=0.8	**Change level:**<br>0.4<br>(95% CI: -1.99 to 2.8)<br>P=0.74<br>**Change trend**<br>-0.01<br>(95% CI: -0.12 to 0.1)<br>P=0.9	**Change level:**<br>0.5<br>(95% CI:-1.87 to 2.88)<br>P=0.68<br>**Change trend:**<br>-0.06<br>(95% CI: -0.17 to 0.05)<br>P=0.28	Untransformed 1.06<br>Prais-Winsten 1.71

For peer review only

For peer review only - http://bmjopen.bmj.com/site/about/guidelines.xhtml

Supplementary Material 9: Annual attendance to the ED in England for head injury

Year	Number head injury primary diagnosis for ED attendance	Proportion attendances primary diagnosis head injury (all attendances)	Proportion attendances primary diagnosis head injury (where primary diagnosis known)
2007/2008	238,099	1.90%	
2008/2009	272,485	2.00%	
2009/2010	336,396	2.2%	3.7%
2010/2011	363,187	2.2%	3.8%
2011/2012	421,221	2.4%	3.8%
2012/2013	423,413	*2.3%*	*3.7%*
2013/2014	449,397	*2.4%*	3.8%
2014/2015	395, 401	*2%*	3.1%
2015/2016	430, 725	*2.1%*	3.2%
2016/2017	449, 584	*2.2%*	3.3%
2017/2018	443, 758	*2.1%*	3.0%

*data obtained from NHS Digital Annual ED reports https://digital.nhs.uk/data-and-information/publications/statistical/hospital-accident--emergency-activity (data was submitted by all hospitals in England from 2012 onwards, prior to this data was only submitted by a variable proportion of hospitals)

For peer review only

STROBE Statement—checklist of items that should be included in reports of observational studies

	Item No	Recommendation
**Title and abstract**	1	(a) Indicate the study's design with a commonly used term in the title or the abstract<br>**Page 1**
		(*b*) Provide in the abstract an informative and balanced summary of what was done and what was found<br>**Page 2**
**Introduction**		
Background/rationale	2	Explain the scientific background and rationale for the investigation being reported<br>**Page 4**
Objectives	3	State specific objectives, including any prespecified hypotheses<br>**Page 4**
**Methods**		
Study design	4	Present key elements of study design early in the paper<br>**Page 5**
Setting	5	Describe the setting, locations, and relevant dates, including periods of recruitment, exposure, follow-up, and data collection<br>**Pages 5,7,8**
Participants	6	(*a*) *Cohort study*—Give the eligibility criteria, and the sources and methods of selection of participants. Describe methods of follow-up<br>**Pages 7,8**
		(*b*) *Cohort study*—For matched studies, give matching criteria and number of exposed and unexposed<br>**N/A**
Variables	7	Clearly define all outcomes, exposures, predictors, potential confounders, and effect modifiers. Give diagnostic criteria, if applicable<br>**Pages 7**
Data sources/ measurement	8*	For each variable of interest, give sources of data and details of methods of assessment (measurement). Describe comparability of assessment methods if there is more than one group<br>**Pages 5,7,8**
Bias	9	Describe any efforts to address potential sources of bias<br>**Pages 7,8**
Study size	10	Explain how the study size was arrived at<br>**N/A**
Quantitative variables	11	Explain how quantitative variables were handled in the analyses. If applicable, describe which groupings were chosen and why<br>**Pages 7,8**
Statistical methods	12	(*a*) Describe all statistical methods, including those used to control for confounding<br>**Pages 7,8**
		(*b*) Describe any methods used to examine subgroups and interactions<br>**Pages 7,8**
		(*c*) Explain how missing data were addressed
		(*d*) *Cohort study*—If applicable, explain how loss to follow-up was addressed<br>*Case-control study*—If applicable, explain how matching of cases and controls was

For peer review only

		addressed<br>*Cross-sectional study*—If applicable, describe analytical methods taking account of sampling strategy
		(*e*) Describe any sensitivity analyses<br>**Page 7,8**
**Results**		
Participants	13*	(a) Report numbers of individuals at each stage of study—eg numbers potentially eligible, examined for eligibility, confirmed eligible, included in the study, completing follow-up, and analysed<br>**Page 9-13**
		(b) Give reasons for non-participation at each stage
		(c) Consider use of a flow diagram
Descriptive data	14*	(a) Give characteristics of study participants (eg demographic, clinical, social) and information on exposures and potential confounders<br>**Page 9-13**
		(b) Indicate number of participants with missing data for each variable of interest
		(c) *Cohort study*—Summarise follow-up time (eg, average and total amount)
Outcome data	15*	*Cohort study*—Report numbers of outcome events or summary measures over time
		Case-control study—Report numbers in each exposure category, or summary measures of exposure
		Cross-sectional study—Report numbers of outcome events or summary measures
Main results	16	(*a*) Give unadjusted estimates and, if applicable, confounder-adjusted estimates and their precision (eg, 95% confidence interval). Make clear which confounders were adjusted for and why they were included<br>**Pages 9-13**
		(*b*) Report category boundaries when continuous variables were categorized
		(*c*) If relevant, consider translating estimates of relative risk into absolute risk for a meaningful time period
Other analyses	17	Report other analyses done—eg analyses of subgroups and interactions, and sensitivity analyses
**Discussion**		
Key results	18	Summarise key results with reference to study objectives<br>**Page 14**
Limitations	19	Discuss limitations of the study, taking into account sources of potential bias or imprecision. Discuss both direction and magnitude of any potential bias<br>**Page 15, 16**
Interpretation	20	Give a cautious overall interpretation of results considering objectives, limitations, multiplicity of analyses, results from similar studies, and other relevant evidence<br>**Pages 16-18**
Generalisability	21	Discuss the generalisability (external validity) of the study results<br>**Pages 16-18**
**Other information**		
Funding	22	Give the source of funding and the role of the funders for the present study and, if applicable, for the original study on which the present article is based<br>**Page 20**

For peer review only - http://bmjopen.bmj.com/site/about/guidelines.xhtml

*Give information separately for cases and controls in case-control studies and, if applicable, for exposed and unexposed groups in cohort and cross-sectional studies.

Note: An Explanation and Elaboration article discusses each checklist item and gives methodological background and published examples of transparent reporting. The STROBE checklist is best used in conjunction with this article (freely available on the Web sites of PLoS Medicine at http://www.plosmedicine.org/, Annals of Internal Medicine at http://www.annals.org/, and Epidemiology at http://www.epidem.com/). Information on the STROBE Initiative is available at www.strobe-statement.org.

For peer review only

For peer review only - http://bmjopen.bmj.com/site/about/guidelines.xhtml
